# Contrasting single-molecule magnet behaviour in dysprosium and terbium bis(stannolediide) complexes

**DOI:** 10.1038/s41557-026-02114-9

**Published:** 2026-04-13

**Authors:** Xiaofei Sun, Alexander Hinz, Stefanie Maier, Sebastian Gillhuber, Da Jin, Masaichi Saito, Florian Weigend, Sören Schlittenhardt, Eufemio Moreno-Pineda, Wolfgang Wernsdorfer, Mario Ruben, Peter W. Roesky

**Affiliations:** 1 Institute for Inorganic Chemistry, Karlsruhe Institute of Technology, Karlsruhe, Germany; 2 Department of Chemistry, Graduate School of Science and Engineering, Saitama University, Shimo-okubo, Sakura-ku, Saitama, Japan; 3 Institute for Quantum Materials and Technologies (IQMT), Karlsruhe Institute of Technology, Eggenstein-Leopoldshafen, Germany; 4 Institute of Nanotechnology, Karlsruhe Institute of Technology, Eggenstein-Leopoldshafen, Germany; 5 Institute of Physical and Theoretical Chemistry, Goethe University Frankfurt, Frankfurt, Germany; 6 Departamento de Química-Física, Universidad de Panamá, Facultad de Ciencias Naturales, Exactas y Tecnología, Panama, Panama; 7 Grupo de Investigación de Materiales, Universidad de Panamá, Facultad de Ciencias Naturales, Exactas y Tecnología, Panama, Panama; 8 Physics Institute, Karlsruhe Institute of Technology, Karlsruhe, Germany; 9 Centre Européen de Science Quantique (CESQ), Institut de Science et d’Ingénierie Supramoléculaires (ISIS, UMR 7006), CNRS-Université de Strasbourg, Strasbourg, France

**Keywords:** Organometallic chemistry, Chemical synthesis, Magnetic materials, Coordination chemistry

## Abstract

The development of single-molecule magnets for high-density data storage has advanced from poly(metallic) cages to lanthanide complexes. Since 2017, highly axial dysprosium bis(cyclopentadienide) complexes have demonstrated exceptional energy barriers to magnetic reversal (*U*_eff_) and high hysteresis temperatures (*T*_H_), with further enhancement achieved in 2025 using bulky amide ligands. Recently, dianionic heavy group 14 cyclopentadienides have emerged as promising ligands due to their higher charge density. Here we report the synthesis of two isostructural homoleptic bis(stannolediide) complexes [Ln(η^5^-L^Sn^)_2_K(thf)_4_] (Ln = Tb(III) or Dy(III)). The Dy(III) complex exhibits single-molecule magnet behaviour with a *U*_eff_ of 1,502(4) K and a blocking temperature of 55 K, whereas the Tb(III) analogue shows Raman-dominated relaxation <~6 K. Removal of the potassium cation yields [Tb(η^5^-L^Sn^)_2_]^−^ or a divalent complex [Dy(η^5^-L^Sn^)_2_]^2−^. The Dy(II) complex displays weak magnetic anisotropy. These results highlight bis(stannolediide) ligands as a promising new class for high‑barrier lanthanide single-molecule magnets.

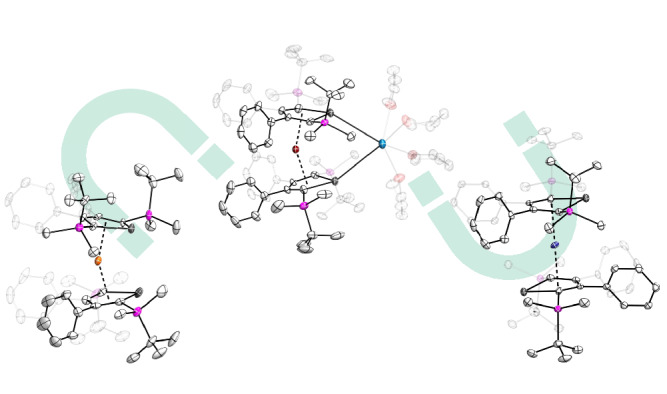

## Abstract (web summary)

Highly axial lanthanide complexes with strong magnetic anisotropy are attractive candidates for high-performance single-molecule magnets. Now dysprosium(III) and terbium(III) homoleptic bis(stannolediide) complexes have been synthesized, with the dysprosium compound exhibiting a high energy barrier to relaxation and a high blocking temperature. Access to a rare divalent Dy(II) analogue displaying magnetic anisotropy has also been demonstrated.

## Main

The observation of a barrier to the relaxation of the magnetization in the archetypal [Mn_12_(µ_3_-O_12_)(CH_3_COO)_16_(H_2_O)_4_]·[2CH_3_COOH]·2H_2_O (or Mn_12_) cage^1^ and, a decade later, in lanthanide phthalocyaninato double-decker [LnPc_2_]^0/−^ complexes^2^, ushered in a new era for inorganic chemists. These molecules exhibit slow relaxation of magnetization due to an energy barrier separating spin-up and spin-down states—giving rise to the concept of single-molecule magnets (SMMs), defined as molecular entities that retain magnetic memory below a characteristic blocking temperature in the absence of an external magnetic field. The potential of exploiting such systems for high-density data storage^3,4^, or as the building blocks of quantum computers^3,5^, triggered a great deal of subsequent research. Later, the realization of the Grover quantum algorithm in a single TbPc_2_ molecule^6^ and surpassing the liquid nitrogen threshold temperature in dysprosocenium and amido complexes^7,8^ became landmark examples of the incorporation of SMMs in advanced quantum technologies.

The progress of high-density storage has evolved gradually from poly(metallic) cages in the 1990s^1,9,10^ to single^2,11–13^ and poly(metallic)^14–16^ lanthanide counterparts. Organometallic lanthanide systems with low coordination numbers show great promise for achieving larger energy barriers^7,17–23^. SMMs are characterized by four key experimental parameters: the effective energy barrier (*U*_eff_) to magnetic reversal, the hysteresis temperature (*T*_H_) at which open magnetic hysteresis loops are observed, the temperature at which opening is observed in the zero-field-cooled (ZFC) field-cooled (FC) traces and the blocking temperature (*T*_B_) at which the magnetization remains observable for 100 s. In 2011, the first organometallic lanthanide SMM [(Cp*)Er(Cot)] (Cp* = C_5_Me_5_ and Cot = C_8_H_8_^2−^) was reported to exhibit a *U*_eff_ of 323 K (ref. ^18^). The enhancement of the magnetic properties of lanthanide SMMs is closely related to their molecular architectures^24–29^. These great leaps were achieved with Dy(III) Cp and Cp-like complexes, thanks to the ligand axiality and their rigid characters. Representative examples include [Dy(Cp^ttt^)_2_]^+^ (Cp^ttt^ = C_5_H_2_*t*Bu_3_-1,2,4) with *T*_H_ of 60 K (refs. ^19,20^), [Dy(Cp^iPr5^)(Cp*)]^+^ (Cp^iPr5^ = C_5_*i*Pr_5_) and [(Cp^iPr5^)_2_Dy_2_I_3_] with *T*_H_ of 80 K (refs. ^7,22^). Very recently, a Dy(III) bis(amide)-alkene complex set a record *T*_H_ of 100 K (ref. ^8^), whereas a mixed Cp*–amide reached *T*_H_ = 73 K (ref. ^23^). Isolobal replacement of a C–R group by a heteroatom fragment has produced further high-performance Dy(III) SMMs^30,31^, including the bis(phospholyl)^32^ and bis(borolediide)^33,34^ Dy cations and anions, respectively, with performance comparable to [Dy(Cp^R^)_2_]^+^ complexes. Analogous Tb(III) species with these heterocyclic ligands remain elusive, whereas carboranyl-type ligands have been used as alternative scaffolds for Dy(III) and Tb(III) SMMs^31,35–37^.

**Fig. 1:**
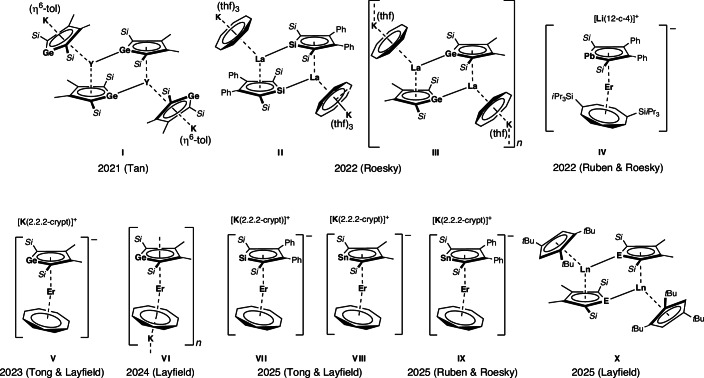
Selected examples of rare-earth metal compounds with dianionic heavy group 14 cyclopentadienides. Si = SiMe_3_ in **I–III**, **V–VIII** and **X**; Si = Si*t*BuMe_2_ in **IV** and **IX**; Si = SiMe_3_, E = Ge or Sn and Ln = Y, Gd or Dy in **X**. thf = tetrahydrofuran^39,41,46,47,49,58,61,62,87^.

In the search for highly axial ligands for Ln SMMs, heavy group 14 cyclopentadienides (silole^38–41^, germole^39,41–47^, stannole^48–55^ and plumbole^40,56–60^) have attracted interest due to their strong donor ability arising from the dianionic charge. Since 2021, these ligands have been introduced into the coordination sphere of rare-earth metals (Fig. 1)^46^. In addition to the π-donor/η^5^-coordination, the silole and germole ligands often also act as σ-donating/η^1^-coordinating ligands (**I–III** and **X**; Fig. 1)^39,46,47,49^, preventing purely axial coordination. Utilizing the heaviest plumbole dianion^58^ (**IV**; Fig. 1) or specific ligand combinations (**V–IX**; Fig. 1)^41,61,62^, some monomeric or polymeric lanthanide complexes without σ-coordination have been accessed. The magnetic properties of all the erbium SMMs (**IV**–**IX**) are primarily influenced by the equatorial crystal field provided by [Cot]^2−^ ligands and heteroatom variation does not substantially impact the SMM properties. Monomeric group 14 dysprosocenate anions [Dy(L)_2_]^−^ (L = dianionic metallole ligand) have remained elusive^50–55^. Here we target monomeric homoleptic Ln bis(stannolediide) complexes to access promising SMM properties.

## Results and discussion

### Synthesis of the dipotassio stannole 1

**Fig. 2:**
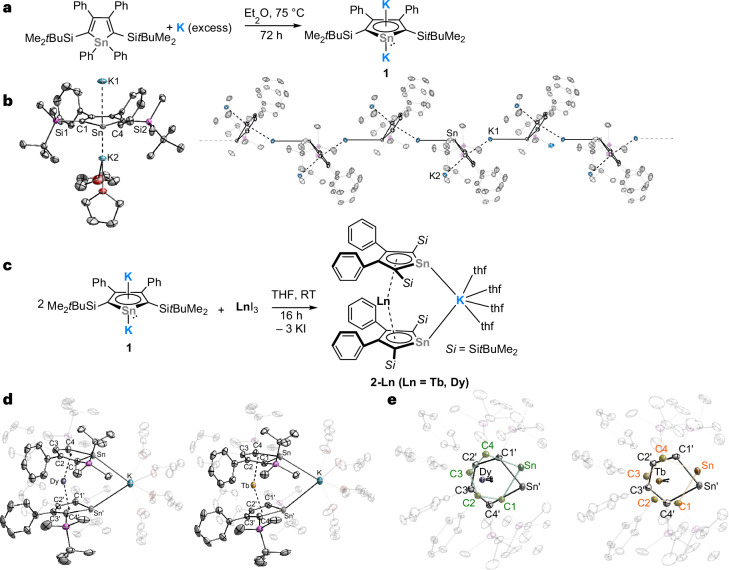
Synthesis of dipotassio stannole **1** and bis(stannolediide) complexes **2-Ln** (Ln = Dy(III) and Tb(III)). **a**, Synthesis of **1** by reduction of 1,1,3,4-tetraphenyl-2,5-bis-(*tert*-butyl-dimethylsilyl)-stannole with potassium. **b**, The asymmetrical unit of complex **1** (left) and a section of the polymeric chain of **1** (right). **c**, Synthesis of **2-Ln** (Ln = Dy(III) and Tb(III)) by reaction of **1** with LnI_3_. **d**, Molecular structures of complexes **2-Dy** (left) and **2-Tb** (right) in the solid state. **e**, Top view of complexes **2-Ln**. The [K(thf)_4_]^+^ fragment is omitted for clarity. All compounds are shown with 50% probability thermal ellipsoids. Hydrogen atoms are omitted for clarity. Selected bond distances and angles are summarized in Supplementary Information. RT, room temperature.

The dilithiostannole [Li_2_(η^5^-L^Sn^)] (L^Sn^ = [1,4-bis-(*tert*-butyl-dimethylsilyl)-2,3-bis-phenyl-stannolediide]) was previously reported by Saito^48,63^. However, attempts to perform salt metathesis reactions of the dilithiostannole with the dysprosium (pseudo)trihalides proved unsuccessful. Consequently, the dipotassio stannole was prepared by reduction of 1,1,3,4-tetraphenyl-2,5-bis-(*tert*-butyl-dimethylsilyl)-stannole with an excess amount of elemental potassium in an analogous manner to the dilithio salt (Fig. 2a)^48,63^. After filtration and evaporation of all volatiles, [K_2_(Et_2_O)_0.45_(η^5^-L^Sn^)] (**1**) was obtained in 69% yield. Single crystals of **1** were obtained from a tetrahydrofuran (THF)/*n*-hexane mixture and the solid-state structure consists of a one-dimensional coordination polymer where each stannole ring is η^5^-coordinated by two K^+^ cations (Fig. 2b). In addition, one K^+^ cation is η^1^-coordinated to the Sn lone pair of the neighbouring stannole ring, forming the polymer chain. The sum of the internal angles in the SnC_4_ ring is 540.0° and all torsion angles are close to 0° (0.03–0.2°), confirming the ring planarity. Together with the similar C–C bond lengths within the SnC_4_ ring, these observations suggest the aromatic nature of the stannole dianion, comparable to its lithium analogue^63^. Quantum chemical calculations indicated that the aromatic character of the stannole ring is retained on coordination (Supplementary Information). The ^119^Sn nuclear magnetic resonance (NMR) spectrum reveals a singlet at 615 ppm, which is shifted to higher frequencies compared to its dilithium analogue (473 ppm)^63^.

### Synthesis of the bis(stannolediide) complexes 2-Ln, 3-Tb and 4-Dy

The bis(stannolediide) compounds [Ln(η^5^-L^Sn^)_2_K(thf)_4_] (**2-Ln**, Ln = Tb(III) or Dy(III)) were synthesized via salt metathesis from LnI_3_ (Ln = Tb or Dy) and [K_2_(Et_2_O)_0.45_(η^5^-L^Sn^)] in a 1:2 molar ratio in THF (Fig. 2c). Dark-red block-shaped crystals were obtained by layering THF with *n*-pentane and the isolated crystals were dried under vacuum for 30 min, yielding 47% **2-Dy** and 57% **2-Tb**. Single-crystal X-ray diffraction (scXRD) analysis revealed the formation of the bis(stannolediide) complexes, in which the potassium cation is η^1^-coordinated to the Sn lone pairs of both stannolediide rings (Fig. 2d,e). To complete the coordination sphere, four additional THF molecules are coordinated to the K^+^ ion. Both complexes crystallized in the monoclinic space group *C*2 with half of the molecule in the asymmetrical unit, with the Ln···K located at the crystallographic *C*_2_ axis. The Ln···K distances of 5.3223(13) Å (**2-Dy**) and 5.3427(8) Å (**2-Tb**) are too long to represent any bonding interaction. As expected, the Ln(III) ion is η^5^-coordinated to both stannole rings and the torsion angle between the two stannole rings is about 31.0° in both complexes (Fig. 2d,e). The distance between the Ln(III) and the centroid of the stannole ring is 2.3039 Å for **2-Dy** and 2.3232 Å for **2-Tb**. The lanthanide–centroid distance in **2-Dy** is even shorter than the respective distances in the bis(phospholide) complex (2.354(3) Å)^32^ and [Dy(Cp^ttt^)_2_]^+^ (2.316(3) Å)^19,20^, but comparable to some other Dy(III) metallocenium ions (2.259(6)–2.340(7) Å)^7,21,33,34^. The lanthanide–centroid distance in **2-Tb** is similar to that in [Tb(Cp^ttt^)_2_]^+^ (2.325(4) Å)^64^ but slightly shorter than in [Tb(Cp^iPr5^)_2_]^+^ (2.356(6) Å)^65^. This highlights the strong electronic effect of the dianionic stannole ring in axial positions, which remains close to the metal despite the steric bulk. The anionic [Ln(η^5^-L^Sn^)_2_]^−^ motif is slightly bent with a Ct–Ln–Ct angle of 154.3° (**2-Dy**) or 153.5° (**2-Tb**). These values lie in the range of previously reported metallocene-type Dy(III) and Tb(III) cations. The **2-Ln** molecules are slightly more linear than the Ln(III) cations [Dy(Cp^ttt^)_2_]^+^ (152.845(2)°)^19,20^, [Tb(Cp^ttt^)_2_]^+^ (152.2(2)°) and [Dy(Cp^iPr4^)_2_]^+^ (147.2(8)°)^21^, but more bent compared to the bis(phospholide) Dy(III) cation (157.94(4)°)^32^ and the bis(borolediide) Dy(III) anions (156.5°, 158.6(3)° and 161.4(3)°)^33,34^.

**Fig. 3:**
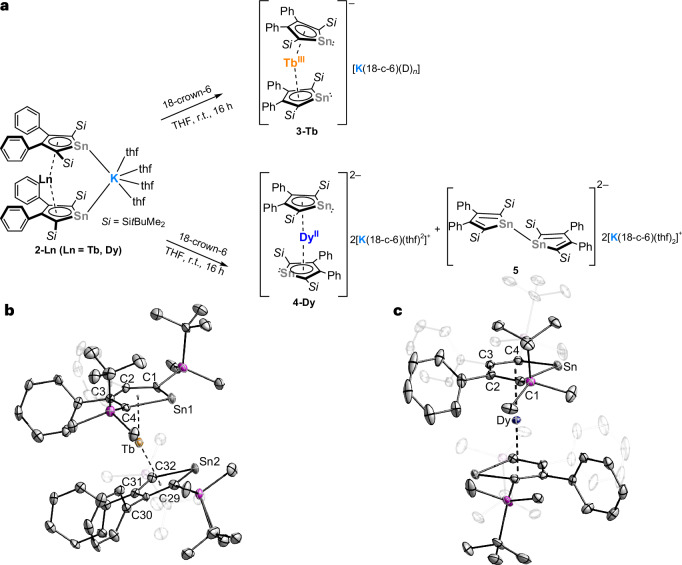
Synthesis of **3-Tb** and **4-Dy**. **a**, Synthesis of **3-Tb** and **4-Dy** by reaction of **2-Ln** with 18-crown-6. Note: the reaction shown below is not balanced, because additional byproducts may be formed: D_*n*_ = (18-c-6)_0.5_ or (thf)_2_. **b**, Molecular structure of the anion of **3-Tb. c**, Molecular structure of the anion of **4-Dy**. All compounds are shown with 50% probability thermal ellipsoids. Hydrogen atoms, cationic fragments and non-coordinating solvent molecules, as well as the cations, are omitted for clarity. Selected bond distances and angles are in Supplementary Information.

The reactions of **2-Ln** with 18-crown-6 allowed the abstraction of the K^+^ ion and enabled the formation of the respective anionic sandwich complexes **3-Tb** and **4**-**Dy** (Fig. 3a). The scXRD analysis revealed that the molecular structures of **3-Tb** and **4-Dy** are charge separated ion pairs: **3-Tb** was isolated as red single crystals from benzene/THF or THF/*n*-hexane solution. Both structures differ by the solvent molecules included in the unit cell (benzene versus THF) and the uncoordinated cationic fragment ({K(18-crown-6)_1.5_} versus {K(18-crown-6)(thf)_2_}). The bond lengths and angles in the anionic part remained similar. Only the THF solvate is discussed below. For the subsequent magnetic investigations, the THF solvate was utilized, whereas dried samples were employed for all other analytical methods.

The molecular structure of **3-Tb** features the expected monoanionic homoleptic Tb(III) sandwich compound (Fig. 3b). Removal of the bridging K^+^ cation slightly linearizes the Ct1–Tb–Ct2 angle (156.3°) and elongates the Tb–Ct distances (Tb–Ct1 2.3439 Å, Tb–Ct2 2.3490 Å) in comparison to **2-Tb**. In contrast, **2-Dy** underwent an unexpected redox reaction and gave the dianionic Dy(II) sandwich complex **4-Dy**. Single crystals of **4**-**Dy** were formed from concentrated THF solution or THF/benzene mixture and the structure is depicted in Fig. 3c. The Dy(II) atom is located at the centre of inversion, which gives a Ct–Dy–Ct’ angle of 180.0° and a *C*_2h_ symmetry in the first proximity. Such linear geometry is rare among lanthanides but is mostly obtained in divalent lanthanide metallocenes^66,67^. This geometry difference between the divalent **4-Dy** and the trivalent **2-Ln** and **3-Tb** is consistent with the geometries of the [(Cp^iPr5^)_2_Dy] (180°)^65^ and [(Cp^iPr5^)_2_Dy]^+^ (162°)^21^. The Dy–Ct distance of 2.2969 Å is almost unchanged compared to **2-Dy** (deviation <0.01 Å). This aligns with the additional bonding contributions in **4-Dy** due to the additional d orbital population (Supplementary Information), as observed in other Dy(II) complexes^65,68,69^. The divalent complex **4-Dy** is the only one where the two Sn atoms are pointing in opposite directions. DFT studies indicated that there is no substantial electronic reason (Supplementary Information). Thus, we expect this to be determined by subtle crystal-packing effects. One of the byproducts in this redox event is the 1,1’-bistannole-dianion [(L^Sn^)_2_]^2−^ (**5**) (Supplementary Fig. 17).

### Magnetic properties

Direct current (DC) magnetic measurements show that both **2-Tb** and **3-Tb** exhibit room temperature *χ*_M_*T*(*T*) values consistent with expectations for single-ion lanthanide systems (Supplementary Fig. 24a) (where *χ*_M_ is the molar magnetic susceptibility). On cooling, the *χ*_M_*T*(*T*) profile of **2-Tb** decreases steadily to 14 K, where a subtle upturn appears, followed by a pronounced drop at lower temperatures. In contrast, **3-Tb** displays a continuous decline in *χ*_M_*T*(*T*) across the full temperature range (Supplementary Fig. 24b). The downturn in *χ*_M_*T*(*T*) reflects thermal depopulation of Stark sublevels, whereas the low-temperature upturn in **2-Tb** indicates substantial magnetic anisotropy.

**Fig. 4:**
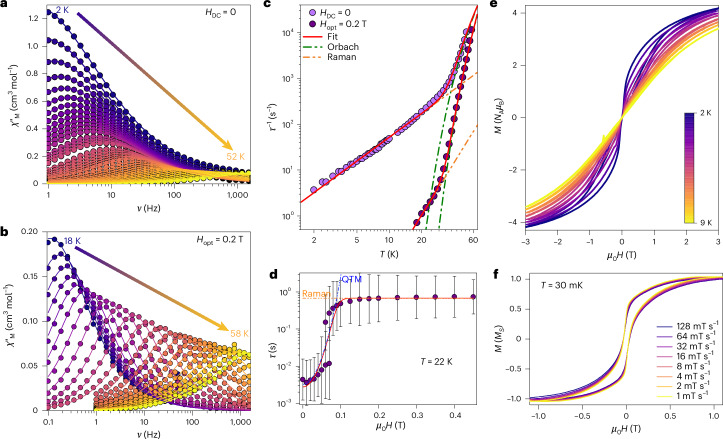
Magnetism of **2-Tb**. **a**,**b**, Frequency-dependent and temperature-dependent *χ*_M_’(*ν*;*T*) at the zero field (**a**) and with *H*_opt_ = 0.2 T (**b**). Solid lines in **a** and **b** are the fits to a generalized Debye model. **c**, *τ*(*T*) for **2-Tb** at zero field (*H*_DC_ = 0) (pink symbols) and *H*_opt_ = 0.2 T (purple symbols). **d**, *τ*(*H*) at a fixed temperature of 22 K. The error bars represent 1 estimated s.d. of the distribution of rates. The solid red lines in **c** and **d** are the best fit utilizing equations (1) and (2), respectively, and parameters in the main text, whereas the green, yellow and blue dashed lines are the Orbach, Raman and QTM contributions to the whole fit, respectively. **e**, *M*(*H*) loops in between 2 K and 10 K and for a field range of ±3 T. **f**, The µSQUID hysteresis loops for a single crystal of **2-Tb** with the field along the easy axis for different sweep rates. The *M*(*H*) curves show an opening between ~±40 mT arising from hyperfine-driven QTM away from the zero field.

To further examine the anisotropic behaviour, alternating current (AC) magnetic susceptibility measurements were conducted. Both Tb(III) complexes exhibit slow magnetic relaxation under zero applied DC field, confirming their SMM character. For **2-Tb**, a frequency-dependent and temperature-dependent maximum in the molar AC magnetic susceptibility (*χ*_M_ʹ(*ν*;*T*)) appears at 1 Hz and 2 K (Fig. 4a and Supplementary Fig. 31), shifting to higher frequencies as the temperature increases. Above 52 K, the relaxation maximum moves beyond the experimental window.

Quantification of the relaxation parameters of **2-Tb** is possible by fitting the temperature-dependent relaxation times (*τ*(*T*)) to equation (1):

1 $${\tau }^{-1}(T\,)={\tau }_{0}^{-1}\exp \left(-\frac{{U}_{\mathrm{eff}}}{{k}_{{\rm{B}}}T}\right)+C{T}^{n}+{\tau }_{\mathrm{QTM}}^{-1}$$

In equation (1), each term represents the Orbach, Raman and quantum tunnelling of the magnetization (QTM) processes, respectively. Fitting the experimental *τ*(*T*) data of **2-Tb** to equation (1) gives: *U*_eff_/*k*_B_ = 422(20) K, *τ*_0_ = 4(2) × 10^−8^ s, *C* = 5.3(6) × 10^−1^ s^−1^ K^−*n*^, *n* = 1.95(4) and *τ*_QTM_ = 0.43(1) s (pink symbols in Fig. 4c). Although QTM is not readily visible in the *τ*(*T*) data, it is expected to be operative, because it is essentially ubiquitous in lanthanide SMMs.

To elucidate the relaxation barrier—often masked by fast relaxation pathways—a static DC field, the optimal field (*H*_opt_), was applied during dynamic measurements. Relaxation dynamics were examined via *τ*(*H*) at 22 K. The resulting *τ*(*H*) profile shows two regimens (Fig. 4d): (1) in the low-field region (0 < *H*_opt_ < 0.07 T), relaxation times remain nearly constant at 10^−2^ s; and (2) beyond 0.1 T, *τ* increases sharply, becoming two orders of magnitude slower. AC susceptibility measurements were then performed on **2-Tb** under *H*_opt_ = 0.2 T (Fig. 4b). At the zero field, the *χ*_M_ʹ(*ν*) maximum at 2 K is centred at ~1 Hz. Under a 0.2-T field, the maximum shifts beyond the experimental window. With the optimal field applied, the lowest temperature at which the maximum is observable is 18 K, with the maximum at ~0.14 Hz, and it moves toward higher frequencies with increasing temperature, up to 58 K. The relaxation parameters extracted under *H*_opt_ = 0.2 T are: *U*_eff_/*k*_B_ = 553(6) K, *τ*_0_ = 9(1) × 10^−9^ s, *C* = 1.2(4) × 10^−6^ s^−1^ K^−*n*^ and *n* = 3.8(1) (purple symbols in Fig. 4c). Notably, the observable maximum shifts from 1 Hz at 2 K (zero field) to 0.14 Hz at 18 K under *H*_opt_ = 0.2 T—corresponding to a temperature shift of 16 K (a 28-K shift when compared to the same frequency (1 Hz)) (compare Fig. 4a–c).

Comparison of the fitted parameters under zero-field and applied-field conditions reveals a striking half-million-fold reduction in the Raman coefficient *C*, likely explaining the pronounced change in AC magnetic dynamics on field application. This highlights the effectiveness of applying an optimal DC field to suppress fast relaxation pathways and reveal the intrinsic relaxation barrier.

To further quantify the relaxation behaviour, we simultaneously fitted the temperature-dependent relaxation times *τ*(*T*) under *H*_opt_ = 0.2 T and the field-dependent relaxation *τ*(*H*) (at 22 K) using equation (2):

2 $${\tau }^{-1}(T;H)={\tau }_{0}^{-1}\exp \left(-\frac{{U}_{\mathrm{eff}}}{{k}_{{\rm{B}}}T}\right)+C{T}^{n}+A{H}^{2}T+{\gamma }_{0}{\exp }^{{\left(-\frac{{\mu }_{0}H}{{H}_{{\rm{c}}}}\right)}^{2}}$$

In equation (2), the first and second terms retain the same meaning as in equation (1), whereas the third and fourth terms represent the field-dependent direct process and the Gaussian dependence of QTM. For the QTM term, *γ*_0_ represents the tunnelling rate, whereas *H*_c_ is the width of the tunnelling process^70–72^. Simultaneous fitting of the *τ*(*T*;*H*) data indicates no direct contribution, yielding: *U*_eff_/*k*_B_ = 552(10) K, *τ*_0_ = 9(2) × 10^−9^ s, *C* = 1.0(8) × 10^−5^ s^−1^ K^−*n*^, *n* = 3.8(2), *γ*_0_ = 4(1) × 10^2^ s^−1^ and *B*_c_ = 3.8(4) T. Notably, no satisfactory fit is obtained using the conventional, Lorentzian field-dependent QTM term (*b*_1_/(1 + *b*_2_*H*^2^))^72,73^, indicating a broader process.

Although QTM is not prominently evident in the temperature-dependent AC susceptibility data for **2-Tb**, hysteresis measurements reveal waist-restricted open magnetic loops between 2 K and 9 K (Fig. 4e). At 2 K, a distinct inflection point is observed at approximately 50 mT, with an S-shaped loop. For a purely electronic transition and given the shortest intermolecular distance of ~13 Å, the expected dipolar field would shift the zero-field QTM crossing to ~4 mT—far below the experimental observation. However, Tb(III) ions (100% natural abundance, *I* = 3/2) introduce substantial hyperfine interactions (*A*_hyp_ ≈ 0.017 cm^−1^, *P* = 0.01 cm^−1^)^74^, which can drive QTM at higher fields (~±80 mT). Most level crossings occur around ~±50 mT (Supplementary Fig. 38), consistent with the feature in the 2-K hysteresis loop, especially for poly(crystalline) samples. Further single-crystal µSQUID^75^ magnetization studies down to 30 mK show open hysteresis loops with an opening of ~40 mT, again matching with nuclear-spin-driven behaviour (Fig. 4f). The presence of hyperfine-split states and the resulting field-driven QTM transitions >±80 mT support employing a Gaussian profile for the field-dependent QTM process.

**Fig. 5:**
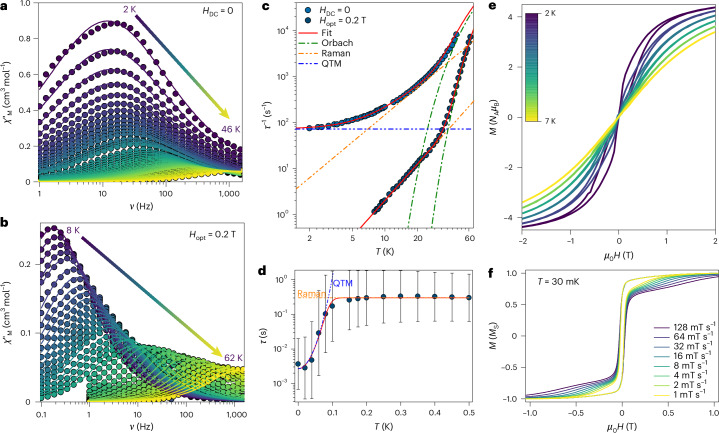
Magnetism of **3-Tb**. **a**,**b**, Frequency-dependent and temperature-dependent *χ*_M_ʹ(*ν*;*T*) at zero field (**a**) and with *H*_opt_ = 0.2 T (**b**). Solid lines in **a** and **b** are the fits to a generalized Debye model. **c**, *τ*(*T*) for **3-Tb** for *H*_DC_ = 0 (dark-blue symbols) and *H*_opt_ = 0.2 T (dark-green symbols). **d**, *τ*(*H*) at a fixed temperature of 22 K. The error bars represent 1 estimated s.d. of the distribution of rates. The solid red lines in **c** and **d** are the best fit utilizing equations (1) and (2), respectively, and parameters in the text, whereas the green, yellow and blue dashed lines are the Orbach, Raman and QTM contributions to the whole fit, respectively. **e**, *M*(*H*) loops in between 2 K and 7 K and for a field range of ±3 T. **f**, The µSQUID hysteresis loops for a single crystal of **3-Tb** with the field along the easy axis for different sweep rates. The *M*(*H*) curves show an opening between ~±30 mT arising from hyperfine-driven QTM away from the zero field.

A notably different behaviour is observed for **3-Tb**, with a maximum of 14 Hz at 2 K (Fig. 5a). The maximum shifts steadily with increasing temperature, reaching the edge of the experimental window at 46 K. The presence of a maximum at such low temperatures, and over a wider range than **2-Tb**, indicates a faster relaxation dynamic. Quantification of these dynamics is also possible. For **3-Tb** at zero field, we obtain: *U*_eff_/*k*_B_ = 236(28) K, *τ*_0_ = 1(1) × 10^−6^ s, *C* = 1.6(2) s^−1^ K^−*n*^, *n* = 1.96(5) and *τ*_QTM_ = 0.01(1) s (pale-blue symbols in Fig. 5c). The *χ*_M_ʹ(*ν*;*T*) data for **3-Tb** are less affected by an applied field (*H*_opt_ = 0.2 T), with the maximum shifting from 14 Hz at 2 K to 0.18 Hz at the same temperature (12-K shift for the maximum centred at 1 Hz) (compare Fig. 5b,c).

For *H*_opt_ = 0.2 T, the *χ*_M_ʹ(*ν*;*T*) maximum shifts from 8 K up to 62 K (Fig. 5b). Fitting *τ*(*T*) yields *U*_eff_/*k*_B_ = 455(2) K, *τ*_0_ = 9.9(4) × 10^−8 ^s, *C* = 4.7(3) × 10^−3^ s^−1^ K^−*n*^ and *n* = 2.64(2) (blue symbols in Fig. 5c). Compared to **2-Tb**, **3-Tb** shows a thousand-fold reduction in the *C* parameter. Simultaneous fitting of the *τ*(*T*;*H*) data (at 0.2 T and 12 K, respectively) to equations (1) and (2) also indicates no contribution of the direct process, giving: *U*_eff_/*k*_B_ = 454(4) K, *τ*_0_ = 1.1(1) × 10^−7^ s, *C* = 4.8(3) × 10^−3^ s^−1^ K^−*n*^, *n* = 2.6(3), *γ*_0_ = 4(1) × 10^2^ s^−1^ and *B*_c_ = 4.0(6) T.

Open hysteresis loops were likewise observed for **3-Tb**, in the temperature range of 2–7 K, with a similar inflection as observed in **2-Tb**. Sub-kelvin µSQUID investigations also revealed open loops of ~30 mT, consistent with nuclear-spin-driven effects in *M*(*H*).

Complete Active Space Self-Consistent Field (CASSCF) calculations show that both **2-Tb** and **3-Tb** possess highly axial electronic structures, with ground-state wavefunctions composed entirely of *m*_*J*_ = ±6 components (that is, 100% ±6; Supplementary Tables 12 and 13). The energy gap between the ground and first excited states is calculated to be 316 cm^−1^ (455 K) for both complexes. Under zero-field conditions, the experimentally determined *U*_eff_ values for both systems are smaller than this separation, suggesting the presence of under-barrier processes^27^. In contrast, the in-field AC data for **3-Tb** shows that the effective barrier matches the ground to first excited state separation, indicating relaxation through this state. For **2-Tb**, however, the experimental barrier exceeds this separation, but remains below the energy of the second excited state. This implies either that the CASSCF calculations do not fully reproduce the energy separation^76,77^ or a more complex relaxation mechanism is operating.

Although the ground and first excited states are nearly colinear in both complexes, a slightly larger deviation is observed in **3-Tb** (Supplementary Tables 12 and 13), which may contribute to the differing relaxation dynamics. Both systems also display a small tunnel splitting, consistent with non-Kramer ions. These results confirm that the stannole ligand field provides sufficient axiality to support slow magnetic relaxation in Tb(III) complexes. Nevertheless, the AC susceptibility data reveal clear differences in relaxation behaviour between **2-Tb** and **3-Tb**. Notably, the experimentally determined Raman exponent *n* is lower than typically expected for non-Kramer ions^78–80^, a deviation often attributed to vibrational contributions to the relaxation mechanism^27,28,81^. To capture this behaviour, the second term in equation (1)—describing the Raman process—can be modified as follows:

3 $${\tau }_{\mathrm{Raman}}^{-1}={C}_{v}\frac{\exp \left(\hslash \omega /{k}_{{\rm{B}}}T\right)}{{\left(\exp \left(\frac{\hslash \omega }{{k}_{{\rm{B}}}T}\right)-1\right)}^{2}}$$

where ω denotes the phonon energy involved in the relaxation pathway. Equation (3) allows us to investigate the anomalously low Raman exponent *n* observed in both **2-Tb** and **3-Tb**, providing a more accurate description of the relaxation dynamics in systems where lattice vibrations are substantial. Fitting the *τ*(*T*) using this vibrationally assisted Raman model reveals a larger *τ*_QTM_ for **2-Tb** compared with **3-Tb** (0.38 s versus 1.37(2) × 10^−2^ s), indicating slower QTM in the former (Supplementary Fig. 37 and Supplementary Table 8). The result agrees well with that from the conventional Raman power law. The extracted ω values fall between ~0.6 cm^−1^ and 2 cm^−1^ under zero-field conditions, consistent with low-energy lattice vibrations. When *H*_opt_ = 0.2 T is applied, QTM is effectively quenched and thus is absent from the in-field data. Under these conditions, we obtain *ω* = 55(12) cm^−1^ for **2-Tb** and *ω* = 18(1) cm^−1^ for **3-Tb**, values characteristic of optical phonon modes^82^, further supporting the role of vibrational contributions in modulating Tb(III) relaxation dynamics.

These results provide a deeper understanding of the magnetic relaxation behaviour in **2-Tb** and **3-Tb**. Under zero and near-zero-field conditions, relaxation is strongly influenced by QTM and thermally assisted QTM,^15^ spanning a field range of roughly ±0.07 T. The broad relaxation window arises from the combined effects of hyperfine interactions and weak intermolecular dipolar coupling. Based on the extracted QTM times, a DC field is expected to suppress QTM more effectively in **3-Tb** than in **2-Tb**. Applying the optimal field *H*_opt_ = 0.2 T shifts both systems out of the QTM-dominated region, leading to a relaxation governed primarily by Raman and Orbach processes, leading processes to >±0.1 T.

A key question arising from this comparison is why **2-Tb** displays superior SMM performance relative to **3-Tb**, despite their similar electronic structures. Analysis of the in-field vibrational Raman phonon energies (Supplementary Table 8) shows that applying a DC field results in higher phonon energies for **2-Tb** than for **3-Tb**. The field lifts the degeneracy of ±*m*_*J*_ states, suppressing low-energy virtual transitions that couple spins to lattice vibrations. By shifting the spin energy levels, the field modifies the phonon modes participating in the two-phonon Raman process, effectively detuning the spin–vibration resonance. This reduces the efficiency of spin–phonon coupling, making vibrationally assisted relaxation less probable and decreasing the overall Raman relaxation rate. Consequently, the diminished phonon-assisted relaxation is likely to contribute to the enhanced SMM behaviour observed in **2-Tb** compared with **3-Tb**. Further investigation of **2-Tb** will be explored in further work.

**Fig. 6:**
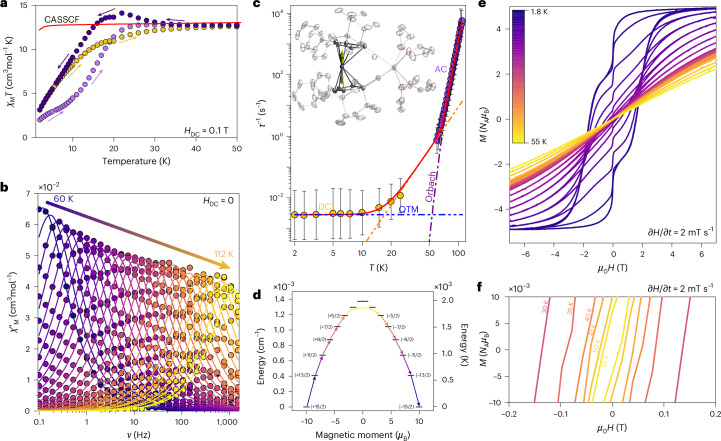
Magnetism of **2-Dy**. **a**, ZFC–FC traces showing the divergent behaviour of the *χ*_M_*T*(*T*) loops, consistent with magnetic blocking in the system for *H*_DC_ = 0.1 T. **b**, Frequency-dependent and temperature-dependent *χ*_M_ʹ(*ν*;*T*) at zero field and zero DC field. The solid lines are the fits to a generalized Debye model. **c**, The *τ*(*T*) data (solid symbols) composed of magnetization decay data (yellow symbols) and AC data (purple symbols). The error bars represent 1 estimated s.d. of the distribution of rates. Dotted lines show the decomposition for each process comprising the overall fit. **d**, CASSCF-obtained energy ladder highlighting an over-the-top relaxation process for **2-Dy**. **e**, *M*(*H*) loops in between 1.8 K and 55 K and for a field range of ±7 T at a field sweep rate of 2 mT s^−1^. **f**, Zoomed region of the hysteresis loops shown in **e**, highlighting the opening of the *M*(*H*) curves up to 55 K.

After the investigation of the Tb(III) systems, we examined the magnetic properties of **2-Dy**. The axial ligand field imposed by the stannole groups, together with the Kramer nature of Dy(III), should ideally produce even larger anisotropy in this analogue. At room temperature, the *χ*_M_*T*(*T*) value matches that expected for an isolated Dy(III) ion, confirming the presence of a single paramagnetic centre (Supplementary Fig. 24c). On cooling, the *χ*_M_*T*(*T*) product shows a slight decrease down to ~50 K, followed by a pronounced rise peaking at 26 K. This low-temperature upturn is a clear indication of magnetic blocking, further supported by the ZFC and FC magnetization traces (Fig. 6a).

Further investigations of the magnetic anisotropy of **2-Dy** was conducted via AC studies. The zero-field magnetic behaviour of **2-Dy** is markedly improved compared with the Tb(III) analogues, showing a larger temperature range at which the *χ*_M_ʹ(*ν*;*T*) displays a maximum—from 60 K to 112 K for frequencies between 0.1 Hz and 1,512 Hz (Fig. 6b). The *τ*(*T*) parameters are extracted by fitting the *χ*_M_ʹ(*ν*;*T*) traces likewise to a Debye model. To probe the *τ*(*T*) <60 K, magnetization decay studies were performed between 2 K and 30 K (see below). Combined fitting of the DC and AC *τ*(*T*) data yields: *U*_eff_/*k*_B_ = 1,502(4) K, *τ*_0_ = 2.3(1) × 10^−10^ s, *C* = 6.7(7) × 10^−9^ s^−1^ K^−*n*^, *n* = 4.56(5) and *τ*_QTM_ = 349(2) s (Fig. 5c). For these parameters and using the temperature at which *τ* = 100 s, an AC *T*_B_ = 58 K is obtained. Hysteresis measurements at a sweep rate of 2 mT s^−1^ confirm these relaxation characteristics, revealing open loops from 1.8 K to 55 K (Fig. 6e). The blocking temperature extracted from the hysteresis loops (*T*_H_) closely matches *T*_B_, indicating that both methods probe similar relaxation timescales. The loops show a rapid drop in *M*(*H*) near zero field, consistent with QTM as commonly observed in highly anisotropic lanthanide systems^8,19,20^. The µSQUID *M*(*H*) loops also display a sharp zero-field process, consistent with QTM; however, no saturation is reached even after 4.5 h (Supplementary Fig. 29b), demonstrating the strong anisotropy of this complex.

Electronic structure calculations align with experimental observations, showing a ground state that is nearly pure (±15/2), with the highest excited state lying at ~1,975 K (Supplementary Table 14). The excited states are found to be rather colinear with the ground state, reducing the relaxation pathway through these states. The experimentally determined *U*_eff_/*k*_B_ corresponds to the fifth excited state, indicating that relaxation proceeds via this level. Notably, the Ct–Ln–Ct angle is 154.3°, suggesting that an even larger anisotropy might be achievable in a more linear Dy(III) analogue. Such an ideal system could, in principle, allow further enhancement of the anisotropic character of this complex.

In contrast, previously reported Dy-SMMs with group 13 boron heterocyclic ligands—namely [(TPhBN)_2_Dy]^−^ (TPhBN = 1-piperidino-2,3,4,5-tetraphenylborolid) and [Dy(BC_4_Ph_5_)_2_]^−^—exhibited higher *U*_eff_ of 2,302 K or 1,871 K and 2,159 K, respectively, together with blocking temperatures of 67 K or 60 K and 65 K^33,34^. Similarly, the phosphorus-containing homoleptic complex [Dy(Dtp)_2_]⁺ (Dtp = 2,5-di-*tert*-butyl-3,4-dimethylphospholyl) showed a *U*_eff_ of 1,760 K—approximately 260 K higher than that of **2-Dy**—yet displayed a markedly lower blocking temperature of 23 K (ref. ^32^).

In contrast to Ln(III) SMMs, Ln(II) SMMs remain rare due to synthetic and stability challenges^65,68,83–85^. To date, only a few divalent lanthanide SMMs have been reported, underscoring the scarcity of these species. Examples include the bis(cyclo-octatetraenediide) Tm(II) complex [Tm(η^8^-COT)_2_{K(18-crown-6)}_2_]^83^, the bis(silylamido) Eu(II) complex [Eu(N{SiMePh_2_}_2_)_2_]^84^ and some divalent Dy and Tb complexes [Ln(Cp^iPr5^)_2_] (Ln = Dy, Tb),^65^ [Ln(NDipp)_2_C*t*Bu_2_] (Ln = Tb, Dy; Dipp = C_6_H_3_*i*Pr_2_-2,6)^85^ and [(C_5_^*i*^Pr_5_)Dy(Cp*)]^68^. Given the strong anisotropy of the trivalent analogues herein investigated, we likewise studied the magnetic anisotropy of **4-Dy**. The gradual decrease of the *χ*_M_*T*(*T*) on cooling hints at a smaller anisotropy than the Dy(III) counterpart (Supplementary Fig. 24d); however, AC studies provide a more quantitative assessment. In sharp contrast to other divalent molecules with linear motifs, this system is not an SMM at zero field and requires the application of a DC field to reveal its SMM behaviour (Supplementary Fig. 40). Fitting the *τ*(*T*) collected for a *H*_opt_ = 90 mT employing equation (1) and considering the Orbach process solely leads to: *U*_eff_/*k*_B_ = 4.5(3) K and *τ*_0_ = 2.0(2) × 10^−5^ s. The results are unexpected, given that structurally similar linear Dy(II) systems exhibit substantial anisotropy^65,68,85,86^.

To probe into the electronic characteristics of this system, we carried out CASSCF investigations. For the divalent nature of **4-Dy**, we defined the active space including 11 roots for a CAS(10,8)SCF of the *S* = 3 state, corresponding to the ground *L* = 5 (H) term, assuming a 4f^9^5d^1^ configuration with a singly occupied, non-degenerate 5d orbital. After convergence, the active space was expanded by adding the nine lowest unoccupied orbitals into a restricted active space framework. This allowed for a single excitation from the complete active space, enabling a configuration interaction calculation across 50 roots of the *S* = 3 spin state. The results show the system to be consistent with a 4f^9^(6s/5d)^1^ configuration, as observed in other systems^65,68,85,86^. Furthermore, a large tunnelling gap is observed for the pseudo-doublet ground state (Supplementary Table 18), hence leading to a substantially reduced anisotropy. Thus, behaviour arises from the bonding interaction between the occupied 5d orbital and the dominant *p* character of the tin atoms of both stannole ligands, as revealed by DFT studies (Supplementary Information). These interactions are maximized in the linear motif with the Ct–Ln–Ctʹ bending angle of 180°. Under these circumstances, the *p* character of the tin ions and the close contact with the 5d orbital of the Dy(II) ion eventually induce an equatorial ligand field, hence diminishing the axial character of the system.

## Conclusion

We have shown the synthesis and promising magnetic properties of homoleptic bis(stannolediide) lanthanide complexes. **2-Tb** and **3-Tb** exhibit SMM behaviour, enabling detailed comparison of magnetic dynamics on potassium decoordination. Magnetic investigations reveal that QTM, driven by hyperfine interactions, dominates relaxation <~0.1 T, whereas Raman and Orbach dominate at higher fields. Application of an optimal 0.2-T field leads to a half-million-fold suppression of the Raman contribution in **2-Tb** and a three-order-of-magnitude reduction in **3-Tb**. The observed differences in effective energy barriers (*U*_eff_/*k*_B_) and Raman relaxation rates probably stem from distinct vibrational modes and their coupling to the phonon bath. **2-Dy** displays strong magnetic anisotropy with a 1,502(4)-K barrier and open hysteresis up to 55 K. Removal of the potassium cation using 18-crown-6 induced an Dy(III)-to-Dy(II) reduction, yielding **4-Dy** exhibiting substantially diminished anisotropy, attributed to its linear geometry and the electronic interplay between Dy(II) and Sn centres. Overall, these findings highlight the magnetic tunability of homoleptic bis(stannolediide) complexes as compelling candidates for the development of next-generation SMMs.

## Methods

All air-sensitive and moisture-sensitive manipulations were performed under dry N_2_ or Ar atmosphere using standard Schlenk techniques or in an Ar-filled MBraun glovebox, unless otherwise stated. *n*-Pentane and toluene were dried using an MBraun solvent purification system (SPS-800) and degassed. THF, n-hexane and benzene were distilled under nitrogen from potassium benzophenone ketyl. THF-*d*_8_ was dried over Na–K alloy and degassed by freeze–pump–thaw cycles. 1,1,3,4-Tetraphenyl-2,5-bis(*tert*-butyldimethylsilyl)stannole^63^ was prepared according to the literature procedures and DyI_3_ was synthesized using an analogue route as the synthesis for the rare-earth trichlorides^88^. All other chemicals were obtained from commercial sources and used without further purification. Elemental analyses were carried out with an Elementar vario MICRO cube. NMR spectra were recorded on Bruker spectrometers (Avance Neo 300 MHz, Avance Neo 400 MHz or Avance III 400 MHz). Chemical shifts were referenced internally using signals of the residual protio solvent (^1^H) or the solvent (^13^C{^1^H}) and are reported relative to tetramethylsilane (^1^H, ^13^C{^1^H}) or externally relative to tetramethylsilane (^29^Si) or tetramethyltin (^119^Sn). All NMR spectra were measured at 298 K, unless otherwise specified. The multiplicity of the signals is indicated as: s for singlet, d for doublet, dd for doublet of doublets, t for triplet, q for quartet, m for multiplet and br for broad. Assignments were determined based on unambiguous chemical shifts, coupling patterns and [^13^C] distortionless enhancement by polarization transfer (DEPT) experiments or two-dimensional correlations ([^1^H–^1^H] correlation spectroscopy (COSY), [^1^H–^13^C] heteronuclear multiple quantum correlation (HMQC) and [^1^H–^13^C] heteronuclear multiple bond correlation (HMBC)). Infrared (IR) spectra were recorded in the region of 4,000 cm^−1^ to 400 cm^−1^ on a Bruker Tensor 37 FTIR spectrometer equipped with a room temperature DLaTGS detector, a diamond attenuated total reflection (ATR) unit and a nitrogen-flushed chamber. In terms of their intensity, the signals were classified into different categories (vs = very strong, s = strong, m = medium, w = weak and sh = shoulder). All experimental magnetometry was carried out on poly(crystalline) samples of the respective compounds.

The samples were placed in a glass tube alongside eicosane using a glovebox. Outside the glovebox, using standard Schlenk techniques, the samples were flame sealed in the glass tubes and the eicosane was gently melted at 40 °C to prevent sample movement. Temperature-dependent susceptibility data were collected in a range 300 K to 2 K using a Quantum Design MPMS 3 SQUID magnetometer on either cooling or heating after cooling the sample to 2 K at zero field or 1,000 Oersted (Oe). The external field during the measurement was 1,000 Oe. All data were corrected for diamagnetic contributions of the sample holder and eicosane based on reference measurements and for the samples’ intrinsic diamagnetism using Pascal’s constants. Field-dependent magnetization data were collected in a maximum range of −7 K to 7 K using a Quantum Design MPMS 3 SQUID magnetometer. Single quadrant *M*(*H*) curves were obtained using the ‘stable at each field’ mode, whereas hysteresis measurements were performed in ‘continuous sweep’ mode with a sweep rate of 50 Oe s^−1^. Due to its long relaxation time at low *T*, the relaxation times for **2-Dy** <30 K were determined using magnetization decay data. The measurements were performed on a Quantum Design MPMS 3 SQUID magnetometer by increasing the field to the maximum of 7 T and a waiting step of 10 min to magnetically saturate the sample. The field was then switched off as fast as possible (700 Oe s^−1^, linear mode) and the magnetization was recorded during this whole procedure. AC susceptibility data were recorded on a Quantum Design MPMS XL SQUID magnetometer in the frequency window of 0.1–1,500 Hz.

Measurements have been performed either with or without an additional external DC field, for every dataset that the DC field is given. The amplitude of the alternating field was 5 Oe. For **2-Dy** ab initio CASSCF, restricted active space state interaction (RASSI) and SINGLE_ANISO calculations were performed using OpenMolcas^89^. The input structure was taken from the X-ray crystallographic structure refinement without further optimization. All basis sets were taken from the internal ANO-RCC library and the size of the basis sets was assigned as follows: Valence Triple Zeta Plus Polarization (VTZP; for Dy), Valence Double Zeta Plus Polarization (VDZP; for Sn, K, O and the 8 Cs of the stannole rings) and Valence Double Zeta (VDZ; all others). CASSCF was performed individually including the highest possible number of configuration interaction roots (21, 224 and 490 for sextet, quartet and doublet, respectively) and, of those, 21, 128 and 130 were employed in the RASSI routine. DFT calculations were done with TURBOMOLE^90^; for details see Supplementary Information.

### Synthesis of complex 1

1,1,3,4-Tetraphenyl-2,5-bis(*tert*-butyldimethylsilyl)stannole (0.700 g, 0.992 mmol) and potassium (0.660 g, 16.8 mmol) were placed in a J. Young Schlenk flask, Et_2_O (about 10 ml) was condensed at −88 °C. The solution was allowed to warm up to room temperature and stirred for 10 min. After freeze–pump–thaw cycles, the mixture was heated at 75 °C for 72 h. Note that heating Et_2_O to 75 °C may potentially lead to explosions. Proper equipment is essential for this process. The deep-red suspension was cooled down to room temperature and extracted with 60 ml of Et_2_O to remove unreacted potassium and insoluble materials. After removal of the solvent, the red solid was washed with 20 ml of *n*-pentane to give the crude product, which is analytically pure and can be used for further reactions without further purification steps. Note that compound **1** is pyrophoric and the yield is 0.450 g, 69% (calculated using 0.45 coordinated Et_2_O, as proven by NMR spectroscopy and elemental analysis): ^1^H NMR (400.3 MHz, THF-*d*_8_): *δ* (ppm) = 6.78–6.74 (m, 8H, *H*_Ph_), 6.68–6.65 (m, 2H, *H*_Ph_), 3.38 (q, ^1^*J* = 7.0 Hz, *C*H_2_ (Et_2_O)), 1.12 (t, ^1^*J* = 7.0 Hz, C*H*_3_ (Et_2_O)), 0.99 (s, 18H, Si*t*BuMe_2_), −0.16 (s, 12H, Si*t*BuMe_2_). ^13^C{^1^H} NMR (100.67 MHz, THF-*d*_8_): *δ* (ppm) = 176.1 (*C*_α_), 155.8 (*C*_β_), 145.9 (*C*_Ph_, q), 132.0 (*C*_Ph_), 126.6 (*C*_Ph_), 122.5 (*C*_Ph_), 66.7 (*C*H_2_, Et_2_O), 30.6 (Si*t*BuMe_2_), 19.1 (Si*t*BuMe_2_), 16.1 (*C*H_3_, Et_2_O), 4.1 (Si*t*Bu*Me*_2_). ^29^Si{^1^H} NMR (79.52 MHz, THF-*d*_8_): *δ* (ppm) = -5.9. ^119^Sn NMR (101.0 MHz, THF-*d*_8_): *δ* (ppm) = 615.5. Anal. Calcd. for C_28_H_49_K_2_Si_2_Sn·0.45 (Et_2_O) (663.06): C 53.98; H 6.76. Found: C 53.86, H 6.29. IR (ATR): wavenumber ($\mathop{v}\limits^{ \sim }$; cm^−1^) = 412 (vw), 448 (w), 494 (w), 559 (w), 594 (m), 633 (m), 653 (m), 698 (s), 762 (s), 797 (s), 815 (s), 952 (s), 1,003 (w), 1,022 (w), 1,071 (m), 1,098 (w), 1,139 (w), 1,153 (w), 1,178 (w), 1,196 (m), 1,237 (s), 1,329 (m), 1382 (m), 1403 (w), 1,438 (m), 1,466 (m), 1,487 (m), 1,587 (m), 2,845 (vs), 2,881 (s), 2,922 (vs), 29,43 (vs), 3,014 (w), 3,051 (w), 3,068 (w).

### Synthesis of complex 2-Tb

The dipotassium stannole **1** (0.144 g, 0.217 mmol) and anhydrous TbI_3_ (0.058 g, 0.108 mmol) were placed together in a J. Young Schlenk flask. THF (about 5 ml) was condensed to the flask at −88 °C and the solution was allowed to warm up to room temperature and stirred for 16 h at room temperature. During this time, colourless insoluble materials (KI) formed. After filtration, the solution was concentrated to about 2 ml and layered with *n*-pentane. After 2 weeks, block-shaped dark-red crystals formed. The solution was carefully decanted and the crystals were isolated. Crystalline yield: 0.088 g, 54%. Anal. Calcd. for C_56_H_80_TbKSi_4_Sn_2_·3 (C_4_H_8_O) (1,517.36): C 53.83; H 6.91. Found: C 54.34, H 6.94. IR (ATR): $\mathop{v}\limits^{ \sim }$ (cm^−1^) = 386 (s), 395 (s), 447 (s), 472 (m), 486 (s), 515 (m), 532 (m), 539 (m), 564 (m), 590 (m), 622 (m), 629 (m), 659 (s), 676 (m), 699 (vs), 729 (m), 764 (vs), 804 (vs), 821 (s), 863 (w), 875 (w), 913 (w), 955 (m), 982 (w), 1,005 (m), 1,021 (w), 1,050 (w), 1,069 (w), 1,154 (w), 1,182 (w), 1,191 (m), 1,243(s), 1,313 (w), 1,331 (w), 1,357 (w), 1,385 (w), 1,405 (w), 1,439 (w), 1,461 (m), 1,469 (m), 1,488 (w), 1,593 (w), 2,849 (m), 2,880 (m), 2,923 (m), 2,948 (m), 3,053 (vw).

### Synthesis of complex 2-Dy

Dipotassium stannole **1** (0.120 g, 0.180 mmol) and anhydrous DyI_3_ (0.049 g, 0.090 mmol) were placed together in a J. Young Schlenk flask. THF (about 5 ml) was condensed to the flask at −88 °C and the solution was allowed to warm up to room temperature and stirred for 16 h at room temperature. During this time, colourless insoluble materials (KI) formed. After filtration, the solution was concentrated to about 2 ml and layered with *n*-pentane. After 2 weeks, block-shaped dark-red crystals formed. The solution was carefully decanted and the crystals were isolated. Crystalline yield: 0.064 g, 47%. Anal. Calcd. for C_56_H_80_DyKSi_4_Sn_2_·3 (C_4_H_8_O) (1,520.94): C 53.70; H 6.89. Found: C 53.53; H 6.58. IR (ATR): $\mathop{v}\limits^{ \sim }$ (cm^−1^) = 408 (vw), 448 (vw), 485 (w), 567 (w), 575 (w), 590 (w), 625 (w), 657 (m), 676 (m), 690 (m), 704 (m), 764 (s), 797 (s), 821 (s), 864 (w), 895 (w), 913 (w), 956 (m), 1,003 (w), 1,024 (w), 1,053 (m), 1,069 (w), 1,155 (w), 1,190 (m), 1,241 (s), 1,317 (w), 1,356 (w), 1,384 (w), 1,405 (w), 1,440 (m), 1,466 (m), 1,489 (m), 1,573 (w), 1,593 (w), 2,697 (w), 2,847 (vs), 2,875 (s), 2,920 (vs), 2,947 (s), 3,053 (w).

### Synthesis of complex 3-Tb

Complex **2-Tb** (0.051 g, 0.033 mmol) and 18-crown-6 (0.018 g, 0.068 mmol) were placed together in a J. Young Schlenk flask. THF (about 2 ml) was added to the flask and the solution was evaporated under reduced pressure until only a tiny amount of THF was left. About 3 ml of benzene was added to the flask. After 1 d, red crystals were formed. Crystalline yield: 0.023 g, 38%. Alternatively, complex **3-Tb** can be crystallized from a THF–*n*-hexane mixture. Crystalline yield: 0.026 g, 44%. Anal. Calcd. for C_74_H_116_TbKO_9_Si_4_Sn_2_·2(C_6_H_6_) (1,853.74): C 55.72; H 6.96. Found: C 55.83, H 6.70. IR (ATR): $\mathop{v}\limits^{ \sim }$ (cm^−1^) = 386 (m), 395 (m), 408 (m), 434 (w), 447 (w), 472 (w), 492 (m), 530 (m), 567 (m), 591 (m), 622 (w), 629 (w), 656 (m), 678 (m), 699 (s), 765 (s), 804 (s), 822 (s), 855 (m), 863 (m), 945 (s), 957 (s), 985 (m), 1,005 (m), 1,022 (m), 1,072 (m), 1,106 (vs), 1,185 (w), 1,243 (s), 1,286 (w), 1,297 (w), 1,352 (m), 1,384 (w), 1,442 (w), 1,469 (m), 1,488 (w), 1,593 (w), 2,848 (m), 2,917 (m), 2,946 (m), 3,049 (vw).

### Synthesis of complex 4-Dy

Complex **2-Dy** (0.050 g, 0.033 mmol) and 18-crown-6 (0.019 g, 0.072 mmol) were placed together in a J. Young Schlenk flask. THF (about 2 ml) was added to the flask and the solution was concentrated and layered with benzene. After 3 d, red crystals were formed. Crystalline yield: 0.025 g, 33%. Alternatively, complex **4-Dy** can be crystallized from a concentrated THF solution. Crystalline yield: 0.023 g, 30%. The yields were calculated relative to the amount of starting material **2-Dy**. Anal. Calcd. for C_96_H_160_DyKO_16_Si_4_Sn_2_·2(C_6_H_6_) (2,317.00): C 55.99; H 7.48. Found: C 56.06, H 7.23. IR (ATR): $\mathop{v}\limits^{ \sim }$ (cm^−1^) = 378 (m), 387 (m), 404 (m), 447 (m), 466 (w), 485 (m), 502 (w), 513 (w), 530 (m), 542 (w), 556 (m), 567 (m), 591 (m), 621 (m), 628 (m), 653 (m), 669 (m), 699 (s), 730 (m), 760 (s), 802 (s), 819 (s), 860 (w), 909 (w), 945 (m), 960 (s), 1,006 (w), 1,022 (w), 1,055 (m), 1,069 (m), 1,103 (vs), 1,182 (w), 1,236 (m), 1,284 (w), 1,351 (m), 1,381 (w), 1,437 (w), 1,454 (w), 1,469 (w), 1,488 (w), 1,590 (w), 2,690 (vw), 2,838 (m), 2,885 (m), 2,913 (m), 2,944 (w), 3,045 (vw).

## Online content

Any methods, additional references, Nature Portfolio reporting summaries, source data, extended data, supplementary information, acknowledgements, peer review information; details of author contributions and competing interests; and statements of data and code availability are available at 10.1038/s41557-026-02114-9.

## Supplementary information

Supplementary Information Supplementary Figs. 1–51 and Tables 1–23.

## Data Availability

All synthetic protocols, spectroscopic data (NMR, IR), detailed crystallographic information, DFT calculations, magnetic measurements and CASSCF calculations can be found in Supplementary Information. Data for this paper are available from the article, Supplementary Information or at radar4chem: 10.22000/x3hmnud45rvac4mg (ref. ^91^). Crystallographic data for the structures reported in this article have been deposited at the Cambridge Crystallographic Data Centre, under deposition nos. CCDC 2429603 (**1**), 2429604 (**2-Dy**), 2429605 (**2-Tb**), 2429606 (**3** (benzene)), 2502348 (**3** (THF)), 2429607 (**4** (benzene)), 2502349 (**4** (THF)), 2502350 (**5**). Copies of the data can be obtained free of charge via https://www.ccdc.cam.ac.uk/structures/.
