# Supplementary material for: Contrasting single-molecule magnet behaviour in dysprosium and terbium bis(stannolediide) complexes
Source: Nat Chem. 2026 Apr 13;18(5):872–81. doi: 10.1038/s41557-026-02114-9 (PMC13148998; doi:10.1038/s41557-026-02114-9)
Supplement: Supplementary file 1 — Supplementary Figs. 1–51 and Tables 1–23. [file 41557_2026_2114_MOESM1_ESM.pdf]

nature chemistry

Supplementary information https://doi.org/10.1038/s41557-026-02114-9

Contrasting single-molecule magnet behaviour in dysprosium and terbium bis(stannolediide) complexes

In the format provided by the authors and unedited

Table of Contents

I. Synthesis and characterization S2

I.1 General procedures S2

I.2 Synthesis of complex **1** S4

I.3 Synthesis of complex **2-Dy** S5

I.4 Synthesis of complex **2-Tb** S6

I.5 Synthesis of complex **3-Tb** S7

I.6 Synthesis of complex **4-Dy** S8

II. NMR spectra S9

III. IR spectra S11

IV. X-ray crystallography S14

IV.1 General methods S14

IV.2 Summary of crystal data S16

V. UV/vis spectra S25

VI. PXRD data of **4-Dy** S28

VII. Magnetism S29

VII.1 General remarks S29

VII.2 DC Magnetic studies S29

VII.3 AC Magnetic studies S33

VII.5 Ab-initio calculations S52

VIII. Density functional theory calculations S59

VIII.1 General remarks S59

VIII.2 Electron configuration of Dy(II) in **4-Dy** S60

VIII.3 Relative stannole ligand orientation S61

VIII.4 Ct-Ln-Ct' angle within **4-Dy** S63

VIII.5 Stannole aromaticity S65

IX. References S70

I. Synthesis and characterization

I.1 General procedures

All air- and moisture-sensitive manipulations were performed under dry N_2 or Ar atmosphere using standard Schlenk techniques or in an argon-filled MBraun glovebox, unless otherwise stated. *n*-Pentane and toluene were dried using an MBraun solvent purification system (SPS-800) and degassed. THF and *n*-hexane were distilled under nitrogen from potassium benzophenone ketyl. THF-d_8 was dried over Na-K alloy and degassed by freeze-pump-thaw cycles. 1,1,3,4-Tetraphenyl-2,5-bis(*tert*-butyldimethylsilyl)stannole[1] was prepared according to the literature procedures and DyI_3 was synthesized using an analog route as for the synthesis of the rare-earth trichlorides.[2] All other chemicals were obtained from commercial sources and used without further purification.

Elemental analyses were carried out with an Elementar vario MICRO cube.

NMR spectra were recorded on Bruker spectrometers (Avance Neo 300 MHz, Avance Neo 400 MHz or Avance III 400 MHz). Chemical shifts are referenced internally using signals of the residual protio solvent (^{1}H) or the solvent ($^{13}C\{^{1}H\}$) and are reported relative to tetramethylsilane (^{1}H, $^{13}C\{^{1}H\}$), or externally relative to tetramethylsilane (^{29}Si), tetramethylstannane (^{119}Sn). All NMR spectra were measured at 298 K, unless otherwise specified. The multiplicity of the signals is indicated as s = singlet, d = doublet, dd = doublet of doublets, t = triplet, q = quartet, m = multiplet and br = broad. Assignments were determined based on unambiguous chemical shifts, coupling patterns and ^{13}C-DEPT experiments or 2D correlations (^{1}H-^{1}H COSY, ^{1}H-^{13}C HMQC and ^{1}H-^{13}C HMBC).

Infrared (IR) spectra were recorded in the region 4000–400 cm^{-1} on a Bruker Tensor 37 FTIR spectrometer equipped with a room temperature DLaTGS detector, a diamond attenuated total reflection (ATR) unit and a nitrogen-flushed chamber. In terms of their intensity, the signals were classified into different categories (vs = very strong, s = strong, m = medium, w = weak, and sh = shoulder).

X-ray powder diffraction (PXRD) was performed on a STOE STADI-MP diffractometer operating with Cu-Kα1-radiation (λ = 154.06 pm) monochromated by a focusing Ge crystal. Powder samples Rietveld refinements were performed with the program TOPAS-Academic (Version 5), using the cif-data to investigate the phase purity of the title compound.

The UV/Vis spectra were recorded using a Mettler-Toledo Spektralphotometer UV7 in quartz cuvettes (d = 1 cm) in solution. To subtract the solvent, the sample was measured relative to the pure solvent.

Synthesis of Starting Materials

1,1,3,4-Tetraphenyl-2,5-bis(*tert*-butyldimethylsilyl)stannole

Ph_2SnCl_2 (6.11g, 0.0177 mol) and the 1,4-dilithio-1,4-bis(tert-butyldimethylsilyl)-2,3-diphenyl-1,3-butadiene (10.5 g, 0.0137 mol) were placed in a 250 mL Schlenk flask. The Schlenk flask was placed in an ice bath and *n*-hexane (100 mL) was transferred, the mixture was stirred at 0 °C for 10 min and was allowed to room temperature. After stirring the reaction solution at room temperature for 16 h, the resulting mixture was concentrated to about half of the original volume, which led to the precipitation of a large amount of white powder. After filtration, the white powder was washed twice with *n*-hexane (10 mL) and redissolved in dichloromethane (120 mL). The insoluble materials were removed by filtration and the solvents (dichloromethane) from the filtrate were removed under reduced pressure. The remaining residue was dried under vacuum for 30 min, giving the title compound as white powder (7.2 g, 58%). The purity was confirmed by ^{1}H NMR spectroscopy.

^{1}H NMR (400.3 MHz, THF-d_8): δ (ppm) = 8.02-7.88 (m, 4H, H_{Ph}), 7.34-7.29 (m, 4H, H_{Ph}), 7.23-7.19 (m, 2H, H_{Ph}), 6.99-6.97 (m, 4H, H_{Ph}), 6.91-6.81 (m, 6H, H_{Ph}), 0.72 (s, 18H, Si*t*<u>*Bu*</u>Me$_2$), –0.12 (s, 12H, Si*t*Bu<u>*Me*$_2$</u>).

The NMR spectrum is available at radar4chem 10.22000/x3hmnud45rvac4mg.

DyI_3

1. Step: Synthesis of $(NH_4)_3DyI_6$. To a Schlenk flask containing Dy_2O_3 (5.0 g, 0.0134 mol) was slowly added 120 mL of concentrated HI. The mixture was stirred for 20 min at room temperature and gently heated until everything dissolved. Subsequently, NH_4I (11.7 g, 0.0807 mol) was added under inert conditions to the clear solution and the reaction mixture was stirred for 30 min. The solution was evaporated to dryness and the remaining residue was dried for another 2 h at 80 °C under vacuum.

2. Step: Thermal decomposition of $(NH_4)_3DyI_6$ to DyI_3. All residue of step 1 was then placed into a glastube and positioned in an oven and heated under dynamic vacuum. The temperature was kept for 6 h at 120 °C, 24 h at 240 °C and 6 h at 360 °C. The glastube was allowed to cool to room temperature and DyI_3 was obtained as white powder and used without further purification.

I.2 Synthesis of complex 1

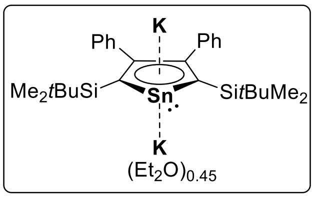

To 1,1,3,4-tetraphenyl-2,5-bis(*tert*-butyldimethylsilyl)stannole[1] (0.700 g, 0.992 mmol) and potassium chunk (0.660 g, 16.8 mmol) placed in a J. Young Schlenk flask, Et_2O (*ca.* 10 mL) was condensed at -88 °C. The solution was allowed to warm up to room temperature and stirred for 10 min. After freeze-pump-thaw cycles, the mixture was heated at 75 °C for 72 h. The deep red suspension was cooled down to room temperature, extracted with 60 mL Et_2O to remove unreacted potassium and insoluble materials. After removal of the solvent, the red solid was washed with 20 mL of *n*-pentane to give the crude product, which is analytically pure and can be used for further reactions without further purification steps.

Yield: 0.450 g, 69%. (Calculated with 0.45 coordinated Et_2O, as proven by NMR spectroscopy and elemental analysis).

^{1}H NMR (400.3 MHz, THF-d_8): δ (ppm) = 6.78-6.74 (m, 8H, H_{Ph}), 6.68-6.65 (m, 2H, H_{Ph}), 3.38 (q, ^{1}J = 7.0 Hz, CH_2 (Et_2O)), 1.12 (t, ^{1}J = 7.0 Hz, CH_3 (Et_2O)), 0.99 (s, 18H, Si<u>*tBu*</u>Me$_2$), –0.16 (s, 12H, Si*t*Bu<u>*Me*$_2$</u>).

^{13}C{^{1}H} NMR (100.67 MHz, THF-d_8): δ (ppm) = 176.1 (C_α), 155.8 (C_β), 145.9 (C_{Ph}, q), 132.0 (C_{Ph}), 126.6 (C_{Ph}), 122.5 (C_{Ph}), 66.7 (CH_2, Et_2O), 30.6 (Si<u>*tBu*</u>Me$_2$), 19.1 (Si<u>*tBu*</u>Me$_2$), 16.1 (CH_3, Et_2O), 4.1 (Si*t*Bu*Me*$_2$).

^{29}Si{^{1}H} NMR (79.52 MHz, THF-d_8): δ (ppm) = -5.9.

^{119}Sn NMR (101.0 MHz, THF-d_8): δ (ppm) = 615.5.

Anal. Calcd for $C_{28}H_{49}K_2Si_2Sn \cdot 0.45\ (Et_2O)$ (663.06): C 53.98; H 6.76. Found: C 53.86, H 6.29.

IR (ATR): $\tilde{\nu}$ (cm^{-1}) = 412 (vw), 448 (w), 494 (w), 559 (w), 594 (m), 633 (m), 653 (m), 698 (s), 762 (s), 797 (s), 815 (s), 952 (s), 1003 (w), 1022 (w), 1071 (m), 1098 (w), 1139 (w), 1153 (w), 1178 (w), 1196 (m), 1237 (s), 1329 (m), 1382 (m), 1403 (w), 1438 (m), 1466 (m), 1487 (m), 1587 (m), 2845 (vs), 2881 (s), 2922 (vs), 2943 (vs), 3014 (w), 3051 (w), 3068 (w).

I.3 Synthesis of complex 2-Dy

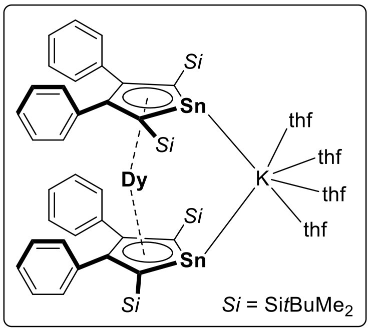

The dipotassium stannole **1** (0.120 g, 0.180 mmol) and anhydrous DyI_3 (0.049 g, 0.090 mmol) were placed together in a J. Young Schlenk flask. THF (*ca*. 5 mL) was condensed to the flask at -88 °C and the solution was allowed to warm up to room temperature and stirred for 16 h at room temperature. During this time, colorless insoluble materials (KI) formed. After filtration, the solution was concentrated to ca. 2 mL and layered with *n*-pentane. After two weeks, block-shaped dark red crystals formed. The solution was carefully decanted and the crystals were isolated.

Crystalline yield: 0.064 g, 47%.

Anal. Calcd for $C_{56}H_{80}DyKSi_4Sn_2\cdot 3\,(C_4H_8O)$ (1520.94): C 53.70; H 6.89. Found: C 53.53; H 6.58.

IR (ATR): $\tilde{\nu}$ (cm^{-1}) = 408 (vw), 448 (vw), 485 (w), 567 (w), 575 (w), 590 (w), 625 (w), 657 (m), 676 (m), 690 (m), 704 (m), 764 (s), 797 (s), 821 (s), 864 (w), 895 (w), 913 (w), 956 (m), 1003 (w), 1024 (w), 1053 (m), 1069 (w), 1155 (w), 1190 (m), 1241 (s), 1317 (w), 1356 (w), 1384 (w), 1405 (w), 1440 (m), 1466 (m), 1489 (m), 1573 (w), 1593 (w), 2697 (w), 2847 (vs), 2875 (s), 2920 (vs), 2947 (s), 3053 (w).

I.4 Synthesis of complex 2-Tb

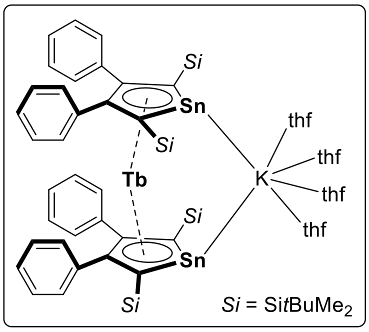

The dipotassium stannole **1** (0.144 g, 0.217 mmol) and anhydrous TbI_3 (0.058 g, 0.108 mmol) were placed together in a J. Young Schlenk flask. THF (ca. 5 mL) was condensed to the flask at -88 °C and the solution was allowed to warm up to room temperature and stirred for 16 h at room temperature. During this time, colorless insoluble materials (KI) formed. After filtration, the solution was concentrated to ca. 2 mL and layered with *n*-pentane. After two weeks, block-shaped dark red crystals formed. The solution was carefully decanted and the crystals were isolated.

Crystalline yield: 0.088 g, 54%.

Anal. Calcd for $C_{56}H_{80}TbKSi_4Sn_2 \cdot 3\ (C_4H_8O)$ (1517.36): C 53.83; H 6.91. Found: C 54.34, H 6.94.

IR (ATR): $\tilde{\nu}$ (cm^{-1}) = 386 (s), 395 (s), 447 (s), 472 (m), 486 (s), 515 (m), 532 (m), 539 (m), 564 (m), 590 (m), 622 (m), 629 (m), 659 (s), 676 (m), 699 (vs), 729 (m), 764 (vs), 804 (vs), 821 (s), 863 (w), 875 (w), 913 (w), 955 (m), 982 (w), 1005 (m), 1021 (w), 1050 (w), 1069 (w), 1154 (w), 1182 (w), 1191 (m), 1243(s), 1313 (w), 1331 (w), 1357 (w), 1385 (w), 1405 (w), 1439 (w), 1461 (m), 1469 (m), 1488 (w), 1593 (w), 2849 (m), 2880 (m), 2923 (m), 2948 (m), 3053 (vw).

I.5 Synthesis of complex 3-Tb

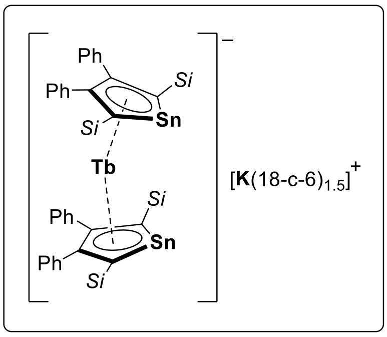

Complex **2-Tb** (0.051 g, 0.033 mmol) and 18-crown-6 (0.018 g, 0.068 mmol) were placed together in a J. Young Schlenk flask. THF (ca. 2 mL) was added to the flask, and the solution was evaporated under reduced pressure until only a tiny amount of THF was left. ca. 3 mL of benzene was added to the flask. After one day, red crystals were formed. Crystalline yield: 0.023 g, 38%. Alternatively, complex **3-Tb** can be crystallized from a THF/*n*-hexane mixture. Crystalline yield: 0.026 g, 44%.

Anal. Calcd for $C_{74}H_{116}TbKO_9Si_4Sn_2{\cdot}2(C_6H_6)$ (1853.74): C 55.72; H 6.96. Found: C 55.83, H 6.70.

IR (ATR): $\tilde{\nu}$ (cm^{-1}) = 386 (m), 395 (m), 408 (m), 434 (w), 447 (w), 472 (w), 492 (m), 530 (m), 567 (m), 591 (m), 622 (w), 629 (w), 656 (m), 678 (m), 699 (s), 765 (s), 804 (s), 822 (s), 855 (m), 863 (m), 945 (s), 957 (s), 985 (m), 1005 (m), 1022 (m), 1072 (m), 1106 (vs), 1185 (w), 1243 (s), 1286 (w), 1297 (w), 1352 (m), 1384 (w), 1442 (w), 1469 (m), 1488 (w), 1593 (w), 2848 (m), 2917 (m), 2946 (m), 3049 (vw).

I.6 Synthesis of complex 4-Dy

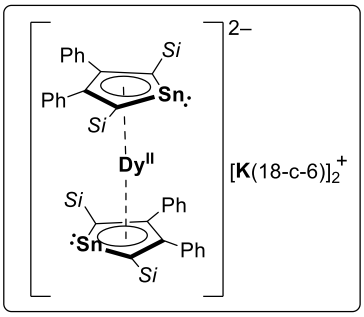

Complex **2-Dy** (0.050 g, 0.033 mmol) and 18-crown-6 (0.019 g, 0.072 mmol) were placed together in a J. Young Schlenk flask. THF (ca. 2 mL) was added to the flask and the solution was concentrated and layered with benzene. After three days, red crystals were formed. Crystalline yield: 0.025 g, 33%. Alternatively, complex **4-Dy** can be crystallized from a concentrated THF solution. Crystalline yield: 0.023 g, 30%. The yields were calculated relative to the amount of starting material **2-Dy**.

Anal. Calcd. for $C_{96}H_{160}DyKO_{16}Si_4Sn_2 \cdot 2(C_6H_6)$ (2317.00): C 55.99; H 7.48. Found: C 56.06, H 7.23.

IR (ATR): $\tilde{\nu}$ (cm^{-1}) = 378 (m), 387 (m), 404 (m), 447 (m), 466 (w), 485 (m), 502 (w), 513 (w), 530 (m), 542 (w), 556 (m), 567 (m), 591 (m), 621 (m), 628 (m), 653 (m), 669 (m), 699 (s), 730 (m), 760 (s), 802 (s), 819 (s), 860 (w), 909 (w), 945 (m), 960 (s), 1006 (w), 1022 (w), 1055 (m), 1069 (m), 1103 (vs), 1182 (w), 1236 (m), 1284 (w), 1351 (m), 1381 (w), 1437 (w), 1454 (w), 1469 (w), 1488 (w), 1590 (w), 2690 (vw), 2838 (m), 2885 (m), 2913 (m), 2944 (w), 3045 (vw).

II. NMR spectra

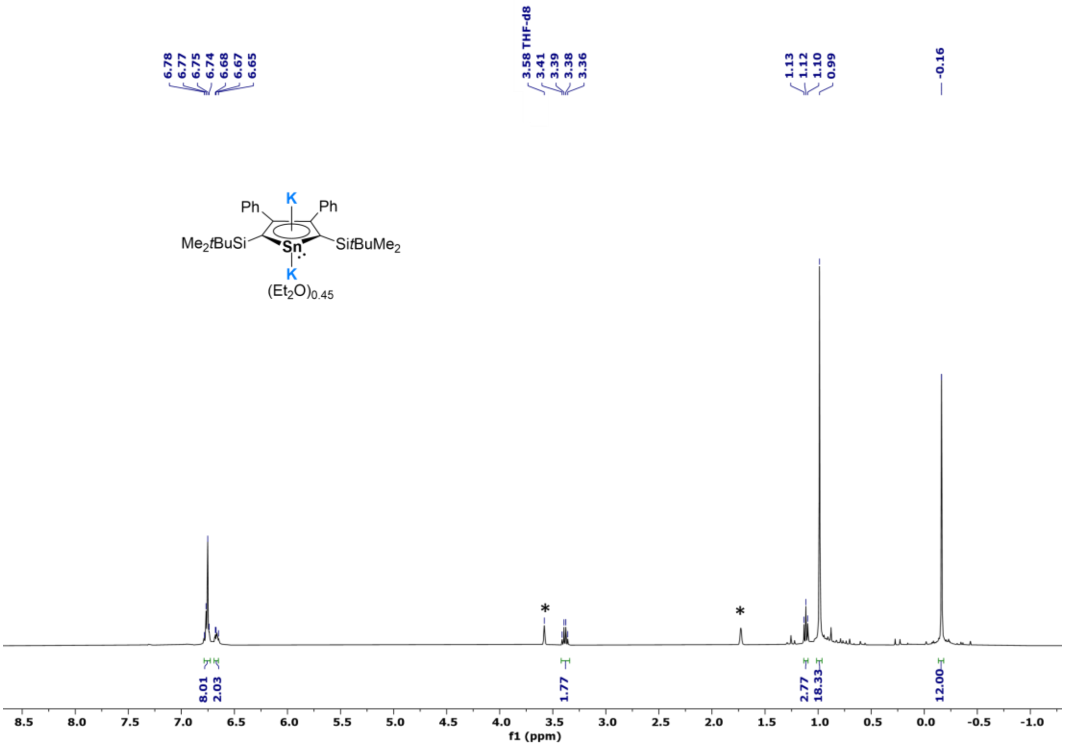

Supplementary Figure 1 ^{1}H NMR spectrum of complex **1** in THF-d_8. *, residual protio solvent signal.

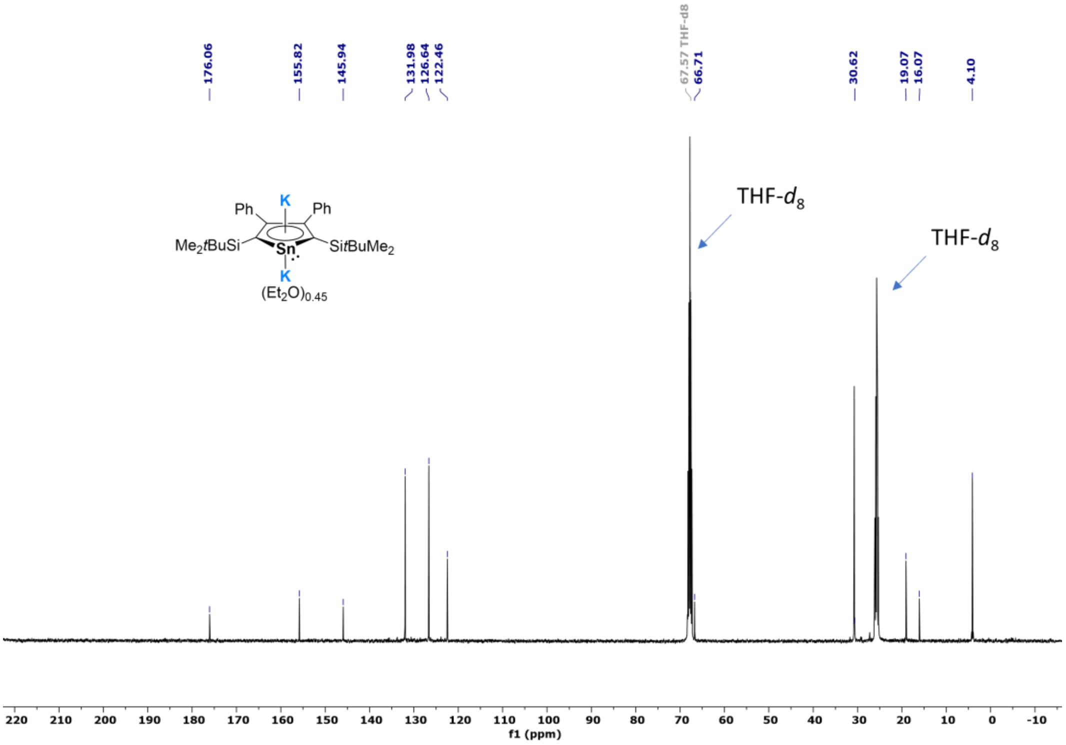

Supplementary Figure 2 $^{13}C\{^{1}H\}$ NMR spectrum of complex **1** in THF-d_8.

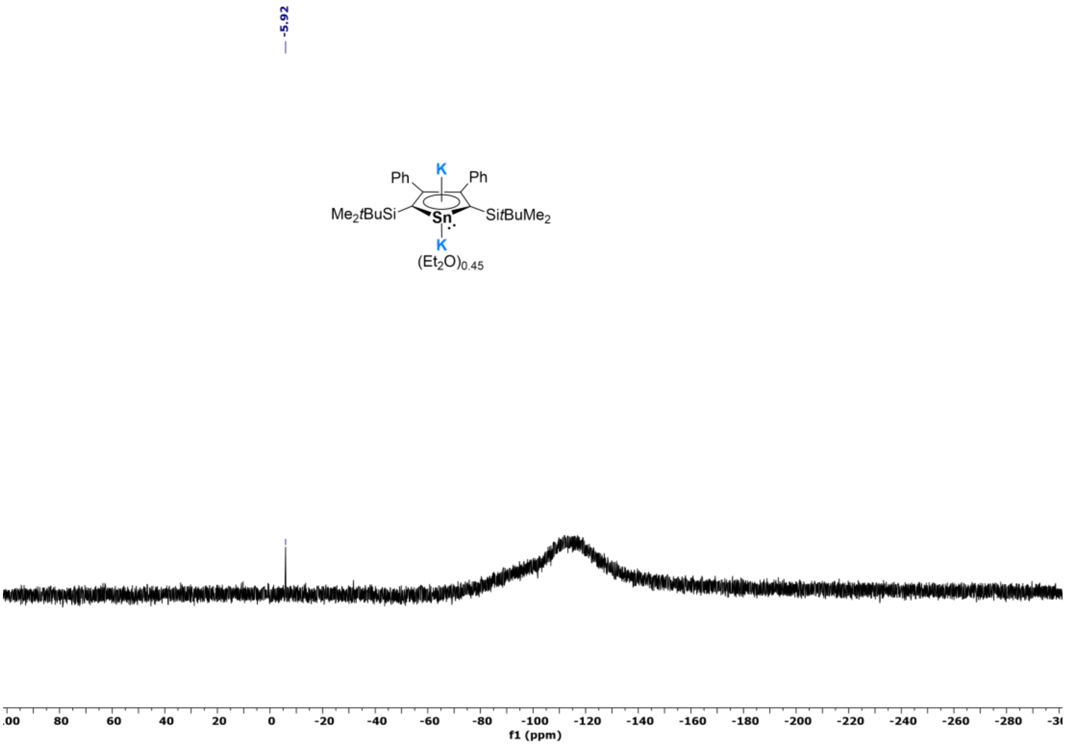

Supplementary Figure 3 $^{29}Si\{^{1}H\}$ NMR spectrum of complex **1** in THF-d_8.

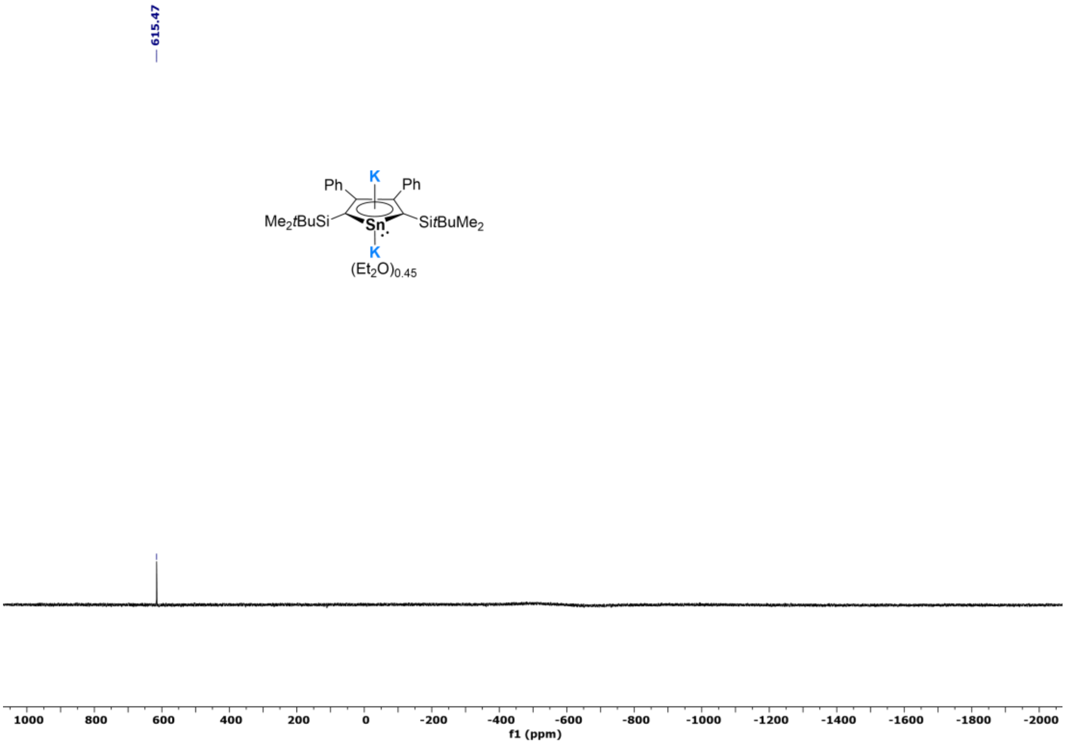

Supplementary Figure 4 ^{119}Sn NMR spectrum of complex **1** in THF-d_8.

III. IR spectra

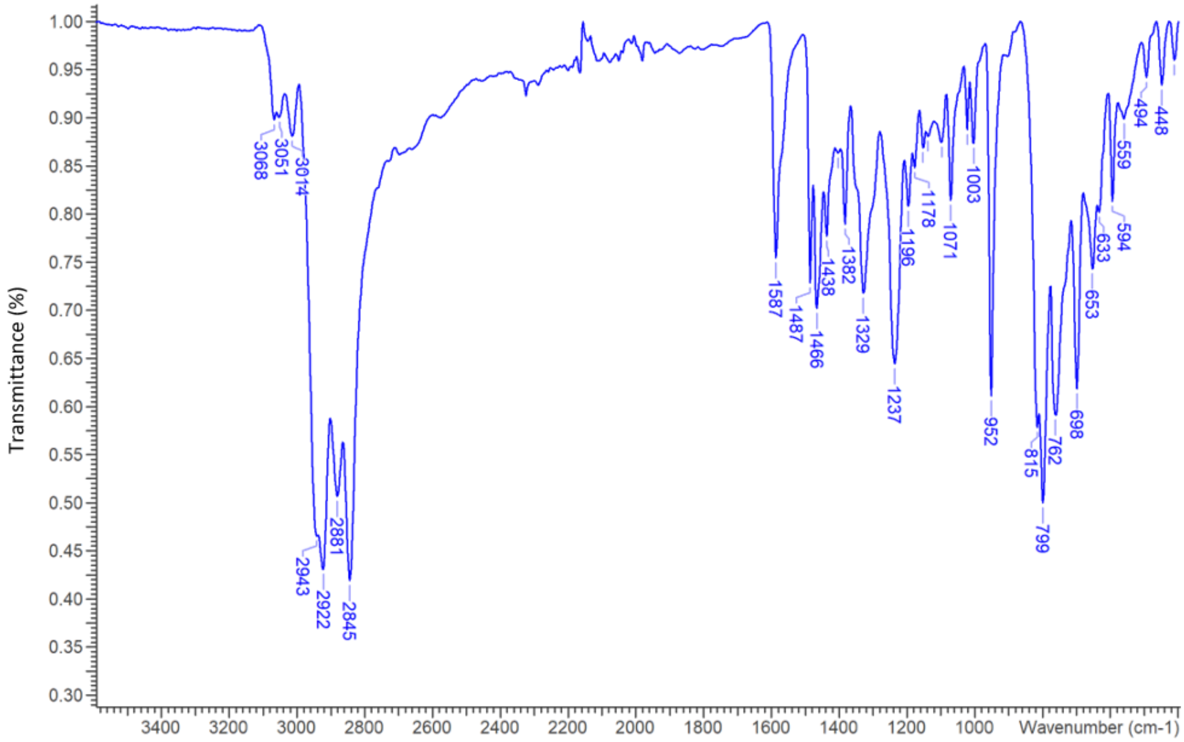

Supplementary Figure 5 IR spectrum of complex **1**.

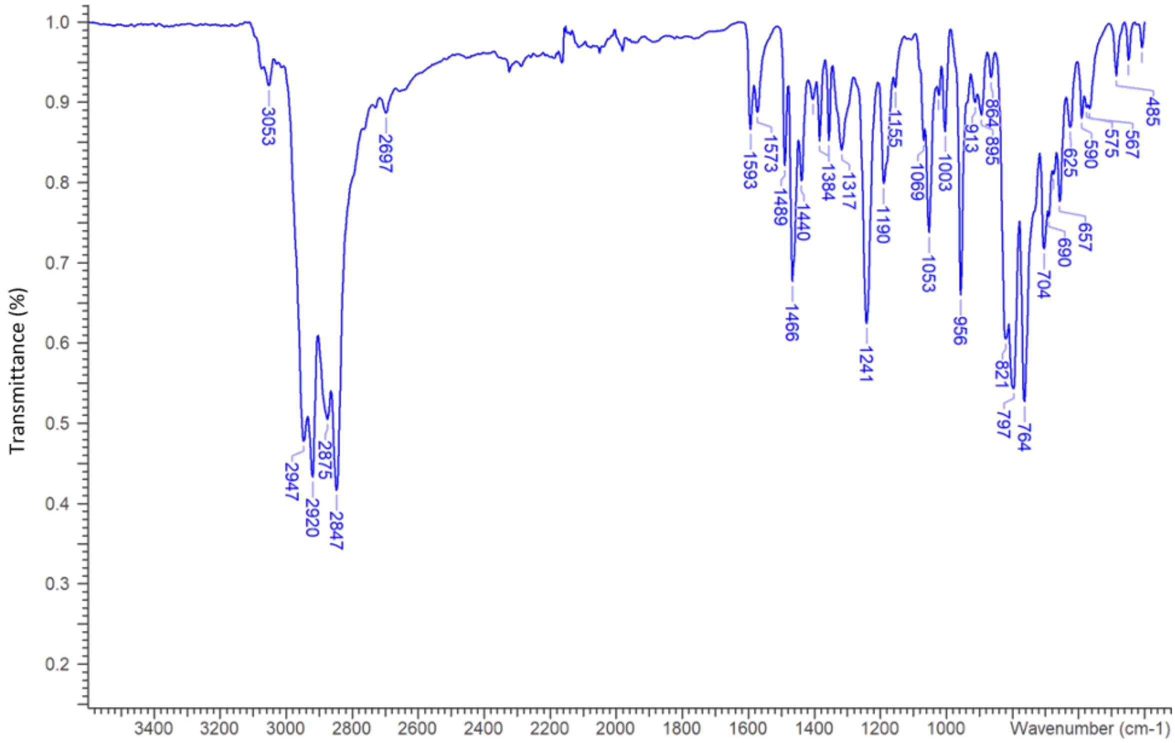

Supplementary Figure 6 IR spectrum of complex **2-Dy**.

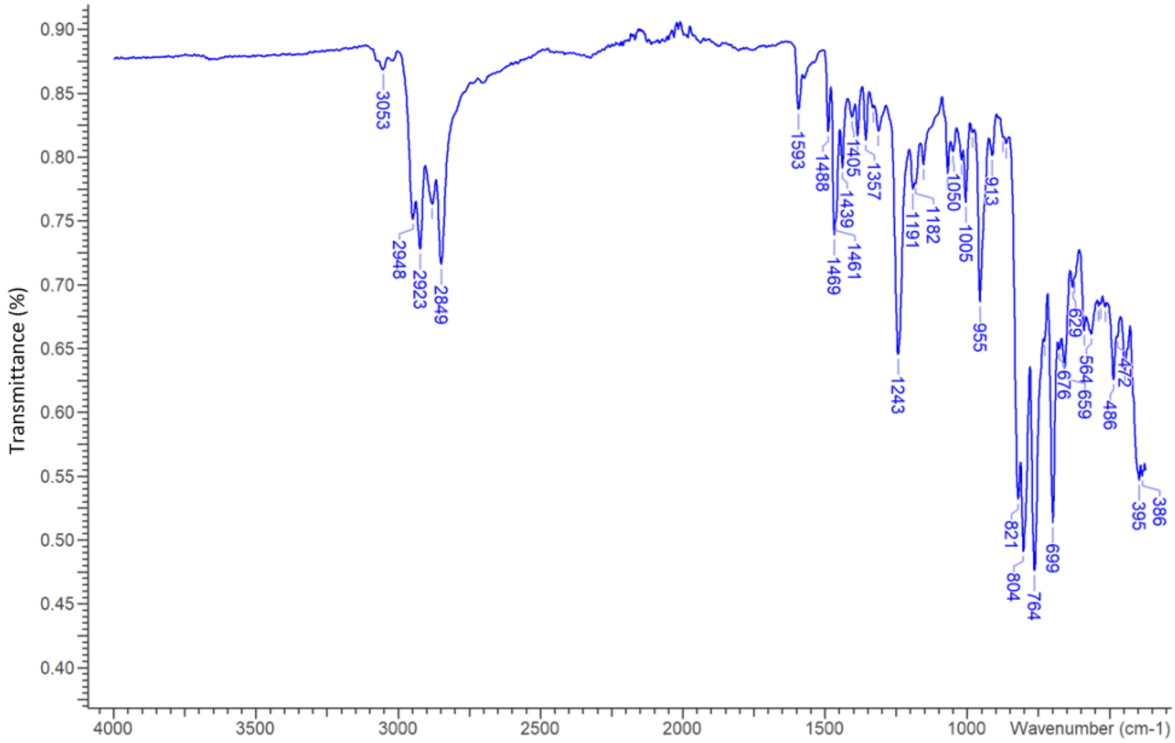

Supplementary Figure 7 IR spectrum of complex **2-Tb**.

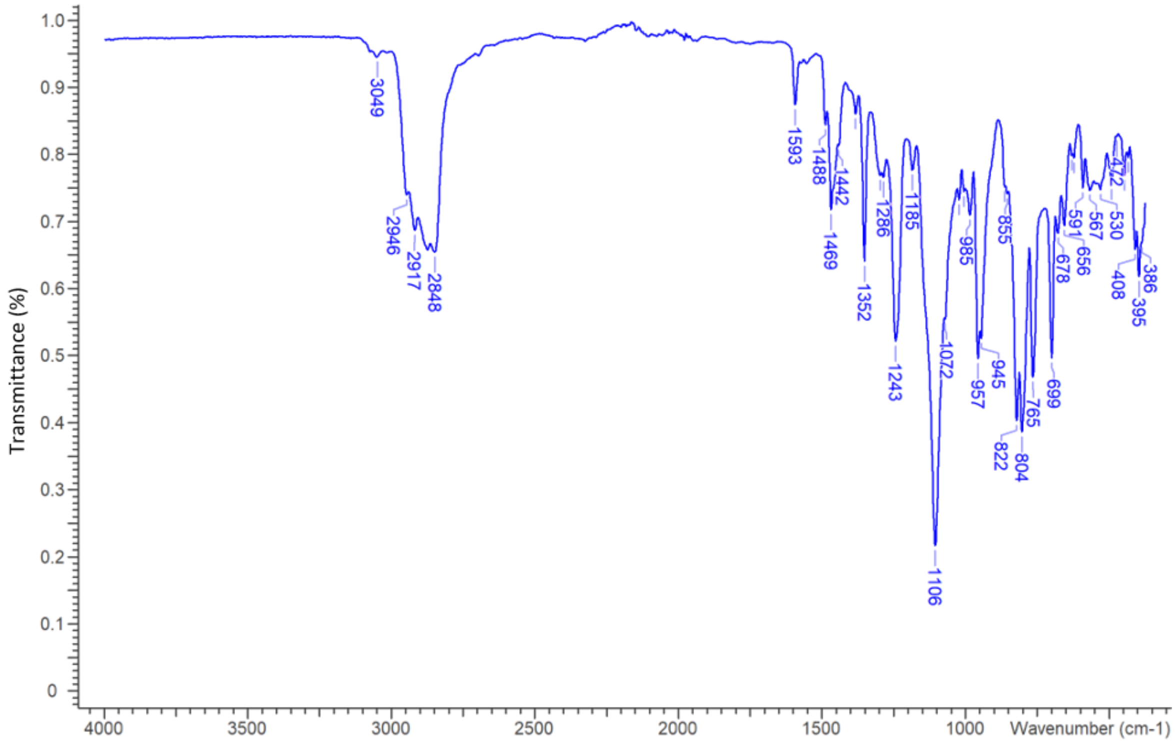

Supplementary Figure 8 IR spectrum of complex **3-Tb**.

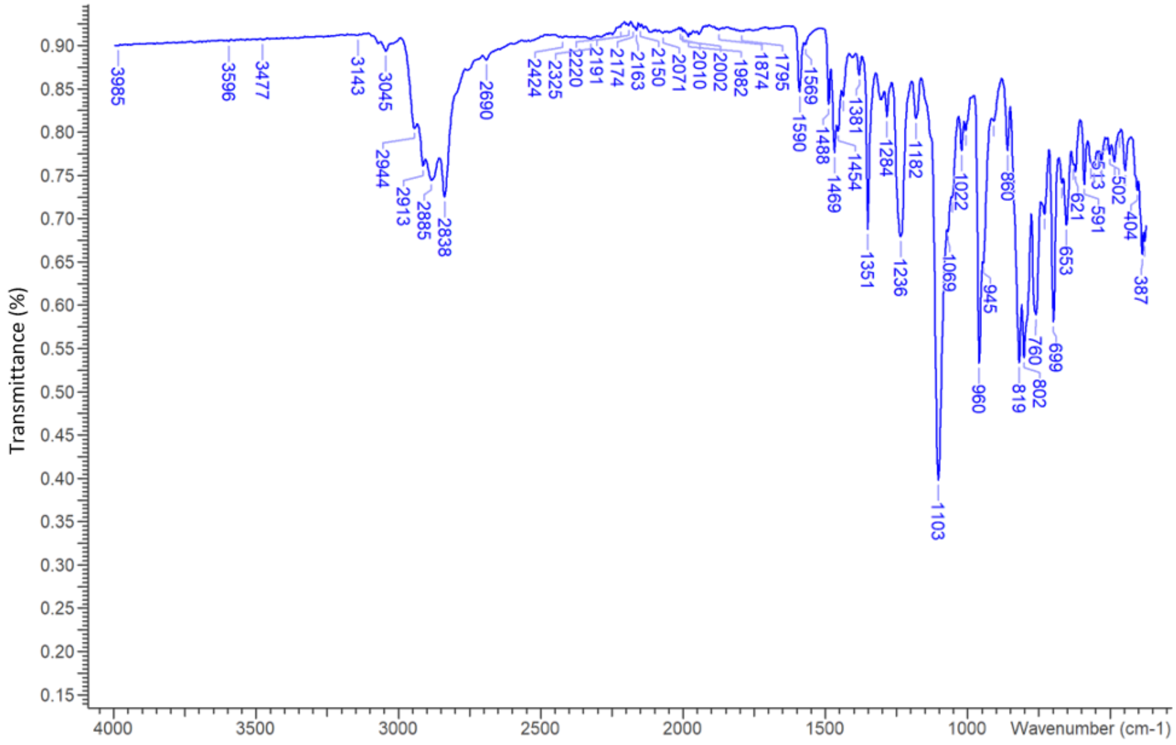

Supplementary Figure 9 IR spectrum of complex **4-Dy**.

IV. X-ray crystallography

IV.1 General methods

Suitable crystals for the X-ray analysis of all compounds were obtained as described above. A suitable crystal was covered in mineral oil (Aldrich) and mounted on a glass fibre. The crystal was transferred directly to the cold stream of a STOE StadiVari (100 K or 110 K) diffractometer. All structures were solved by using the program SHELXS/T[3,4] and Olex2.[5] The remaining non-hydrogen atoms were located from successive difference Fourier map calculations. The refinements were carried out by using full-matrix least-squares techniques on F_o^2 by using the program SHELXL.[3,4] The H-atoms were introduced into the geometrically calculated positions (SHELXL procedures) unless otherwise stated and refined riding on the corresponding parent atoms. In each case, the locations of the largest peaks in the final difference Fourier map calculations, as well as the magnitude of the residual electron densities, were of no chemical significance. Specific comments for each data set are given below. Summary of the crystal data, data collection and refinement for all compounds are given in Supplementary Table 1.

Crystallographic data for the structures reported in this paper have been deposited with the Cambridge Crystallographic Data Centre as a supplementary publication no. CCDC 2429603-2429607 and 2502348-2502350. Copies of the data can be obtained free of charge on application to CCDC, 12 Union Road, Cambridge CB21EZ, UK (fax: (+(44)1223-336-033; email: deposit@ccdc.cam.ac.uk).

The following special comments were applied to the models of the structures:

In the structure of complex **1**, one THF molecule (O2, C33-C36) is disordered over two positions in a ratio of 0.54/0.46.

In the structure of complex **2-Dy**, two THF molecules (O1, C29–C32; O2, C23–C36) are disordered over two positions in a ratio of 0.59/0.41 and 0.69/0.31, respectively. One *t*Bu group (C25–C28) is disordered over two positions in a ratio of 0.60/0.40.

In the structure of complex **2-Tb**, two THF molecules (O1, C29–C32; O2, C23–C36) are disordered over two positions in a ratio of 0.52/0.48 and 0.59/0.41, respectively. One *t*Bu group (C25–C28) is disordered over two positions in a ratio of 0.60/0.40.

In the structure of complex **2-Dy**, one THF molecule (O7, C41–C44) are disordered over two positions in a ratio of 0.83/0.17.

The crystal of **3-Tb** (crystallized from THF/*n*-hexane) consists of two twin domains. It was refined with a twin domain ratio of 0.71/0.29. In the structure, one *t*Bu group is disordered over two positions in a ratio of 0.64/0.36.

In the structure of complex **4-Dy** (crystallized from THF), three THF molecules (O4, C45—C48; O9, C35–C38; C10, C53–C59) are disordered over two positions in a ratio of 0.56/0.44, 0.56/0.44 and 0.57/0.43, respectively. One *t*Bu group (C25–C28) is disordered over two positions in a ratio of 0.61/0.39.

In the structure of complex **5**, one THF molecule (O15, C93-C96) is disordered over two positions in a ratio of 0.56/0.44. Note: one B alert appears in the CheckCIF routine of **5**

Alert level B – PLAT910_ALERT_3_B

Missing FCF Reflection(s) Below Theta(Min) [Deg] = 2.50:

1 0 0, -1 1 0, 0 1 0, 0 -1 1, 1 -1 1, -1 0 1, 0 0 1, 1 0 1, -1 1 1, 0 1 1, 0 -1 2, 1 -1 2, -1 0 2, 0 0 2, -1 1 2, 0 1 2, -1 0 3, 0 0 3

Author Response: The innermost reflections are affected by the beamstop settings.

IV.2 Summary of crystal data

Supplementary Table 1 Crystal data, data collection and refinement for all compounds.

**Compound**	**1**	**2-Dy**	**2-Tb**	**3-Tb**		**4-Dy**		**5**
Formula	$C_{36}H_{56}K_2O_2Si_2Sn$	$C_{72}H_{112}DyKO_4Si_4Sn_2$	$C_{72}H_{112}DyKO_4Si_4Sn_2$	$C_{56}H_{80}TbSi_4Sn_2(C_{18}H_{36}KO_9)(C_6H_6)_4$	$C_{56}H_{80}TbSi_4Sn_2(C_{20}H_{40}KO_8)(C_4H_8O)$	$C_{56}H_{80}DySi_4Sn_2(C_{20}H_{40}KO_8)_2(C_6H_6)$	$C_{56}H_{80}DySi_4Sn_2(C_{20}H_{40}KO_8)_2(C_4H_8O)_3$	$C_{92}H_{152}K_2O_{15}Si_4Sn_2$
μ/mm^{-1}	0.930	1.815	1.762	1.353	1.569	1.244	1.221	0.681
Formula Weight	773.87	1592.95	1589.37	1697.42	1781.58	2316.89	2376.98	1926.07
Colour	orange	red	red	red	red	red	dark red	red
Shape	block-shaped	block-shaped	block-shaped	rod-shaped	plate-shaped	block-shaped	block-shaped	block-shaped
T/K	110	100	100	100	100	100	100	100
Crystal System	orthorhombic	monoclinic	monoclinic	triclinic	triclinic	monoclinic	triclinic	triclinic
Flack Parameter	0.08(2)	0.093(6)	-0.010(8)	–	–	–	–	–
Space Group	$Pna2_1$	$C2$	$C2$	P-1	P-1	$P2_1/c$	P-1	P-1
a/Å	13.8460(8)	21.3271(7)	21.266(3)	13.3209(5)	15.4485(6)	12.9050(5)	13.631(3)	14.1738(7)
b/Å	15.2799(10)	15.0741(4)	15.088(2)	17.1058(7)	15.8245(5)	24.1038(7)	13.918(5)	14.4808(7)
c/Å	19.0006(17)	15.6124(5)	15.622(2)	22.2353(10)	20.6495(8)	18.5351(7)	16.964(7)	28.2248(15)
α/°	90	90	90	83.331(4)	104.516(3)	90	86.76(3)	94.413(4)
β/°	90	131.820(2)	131.760(8)	80.622(3)	100.108(3)	91.850(3)	68.39(3)	102.531(4)
γ/°	90	90	90	79.461(3)	114.154(3)	90	79.73(2)	114.562(4)
V/Å^3	4019.9(5)	3740.5(2)	3739.1(10)	4895.1(4)	4232.4(3)	5762.5(4)	2944.2(18)	5051.4(5)
Z	4	2	2	2	2	2	1	2
Z'	1	0.5	0.5	0.5	1	0.5	0.5	1
Wavelength/Å	0.71073	0.71073	0.71073	0.71073	0.71073	0.71073	0.71073	0.71073
Radiation type	MoK$_\alpha$	MoK$_\alpha$	MoK$_\alpha$	MoK$_\alpha$	MoK$_\alpha$	MoK$_\alpha$	MoK$_\alpha$	MoK$_\alpha$
Θ_{min}/°	1.710	1.862	1.747	1.864	2.121	1.690	2.386	2.498
Θ_{max}/°	27.999	26.000	25.999	25.000	25.000	25.999	32.838	30.042
Measured Refl's.	29614	18750	19556	48988	72164	30723	38516	58420
Indep't Refl's	9599	6410	7300	17175	72164	11257	16184	23778
Refl's I≥2 σ(I)	8278	6274	7041	11866	54414	9645	12561	17853
R_{int}	0.0248	0.0180	0.0213	0.1169	–	0.0252	0.0254	0.0339
GooF	1.015	1.052	1.029	1.016	1.037	1.039	1.028	1.009
wR_2 (all data)	0.0762	0.0433	0.0619	0.1531	0.1069	0.0726	0.0947	0.0768
wR_2	0.0719	0.0432	0.0616	0.1370	0.0955	0.0706	0.0848	0.0731
R_1 (all data)	0.0415	0.0174	0.0270	0.0855	0.0581	0.0337	0.0552	0.0503
R_1	0.0315	0.0169	0.0258	0.0561	0.0380	0.0272	0.0361	0.0316

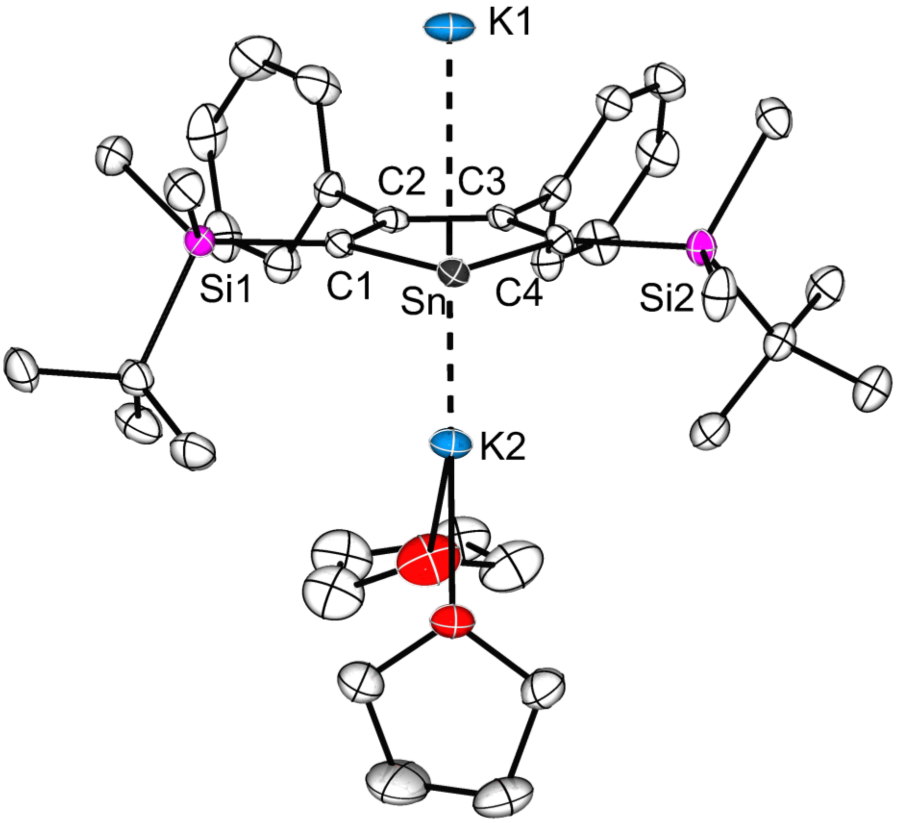

Supplementary Figure 10 Molecular structure of the complex **1** in the solid state with thermal ellipsoids at 50% level. Only the asymmetric unit of the polymeric structure is depicted here. All hydrogen atoms are omitted for clarity. Selected bond distances [Å] and angles [°]: Sn–C1 2.169(4), Sn–C4 2.165(4), C1–C2 1.418(6), C2–C3 1.429(5), C3–C4 1.430(6), K1–C1 3.117(4), K1–C2 2.966(4), K1–C3 2.950(4), K1–C4 3-086(4), K2–C1 3.121(4), K2–C2 2.873(4), K2–C3 2.975(4), K2–C4 3.125(4); C1–Sn–C4 81.17(12), C1–C2–C3 119.5(4), C2–C3–C4 119.0(4), C3–C4–Sn 110.1(3).

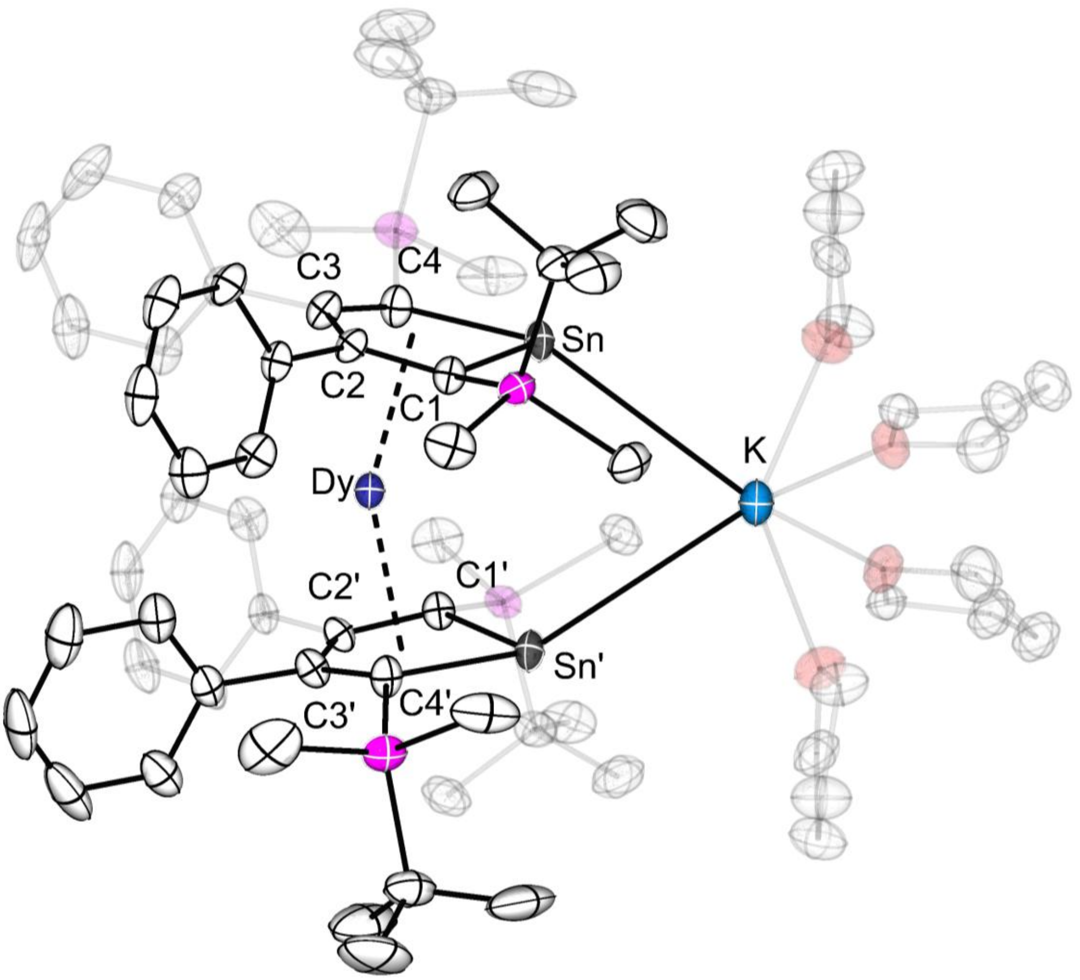

Supplementary Figure 11 Molecular structure of the complex **2-Dy** in the solid state with thermal ellipsoids at 50% level. All hydrogen atoms are omitted for clarity. Selected bond distances [Å] and angles [°]: Sn–C1 2.171(3), Sn–C4 2.171(3), C1–C2 1.423(5), C2–C3 1.423(5), C3–C4 1.423(5), Dy–Ct 2.3039, Sn–K 3.7236(10); C1–Sn–C4 79.30(13), C1–C2–C3 118.3(3), C2–C3–C4 118.2(3), C3–C4–Sn 111.6(2), Ct–Dy–Ct' 154.3.

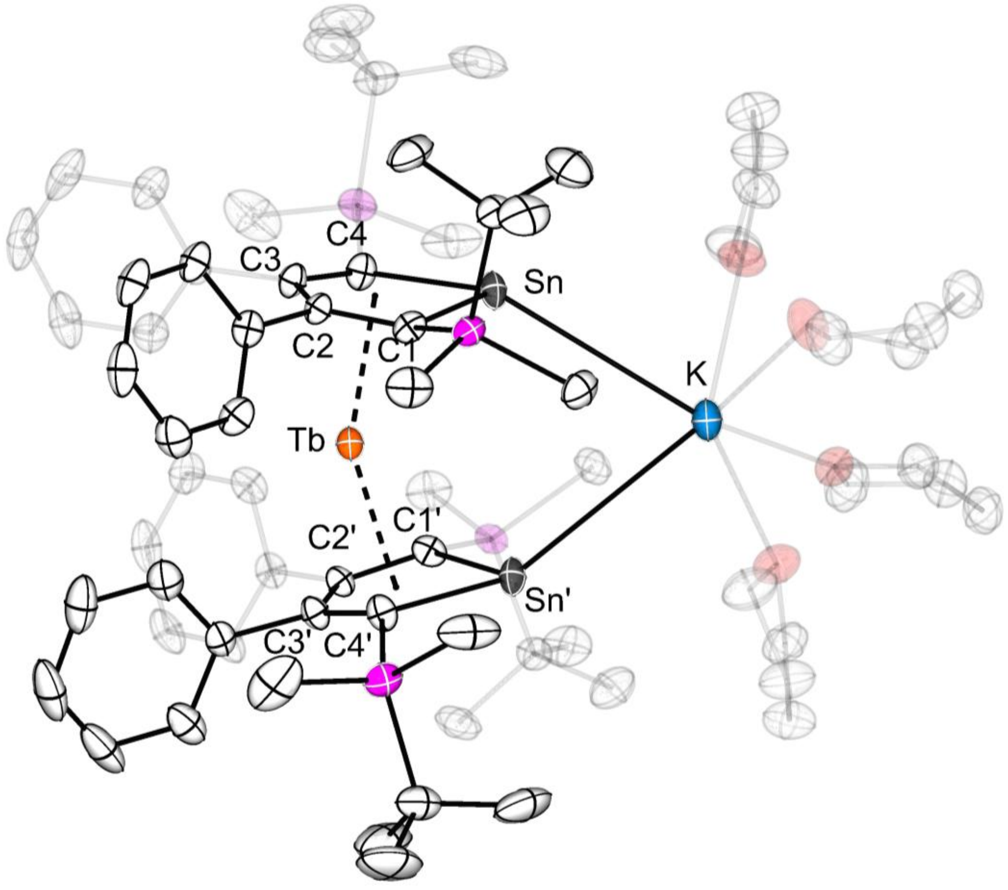

Supplementary Figure 12 Molecular structure of the complex **2-Tb** in the solid state with thermal ellipsoids at 50% level. All hydrogen atoms are omitted for clarity. Selected bond distances [Å] and angles [°]: Sn–C1 2.175(3), Sn–C4 2.165(5), C1–C2 1.419(7), C2–C3 1.429(6), C3–C4 1.420(7), Tb–Ct 2.3232, Sn–K 3.7284(15); C1–Sn–C4 79.3(2), C1–C2–C3 118.3(4), C2–C3–C4 117.7(4), C3–C4–Sn 112.2(3), Ct–Tb–Ct' 153.5.

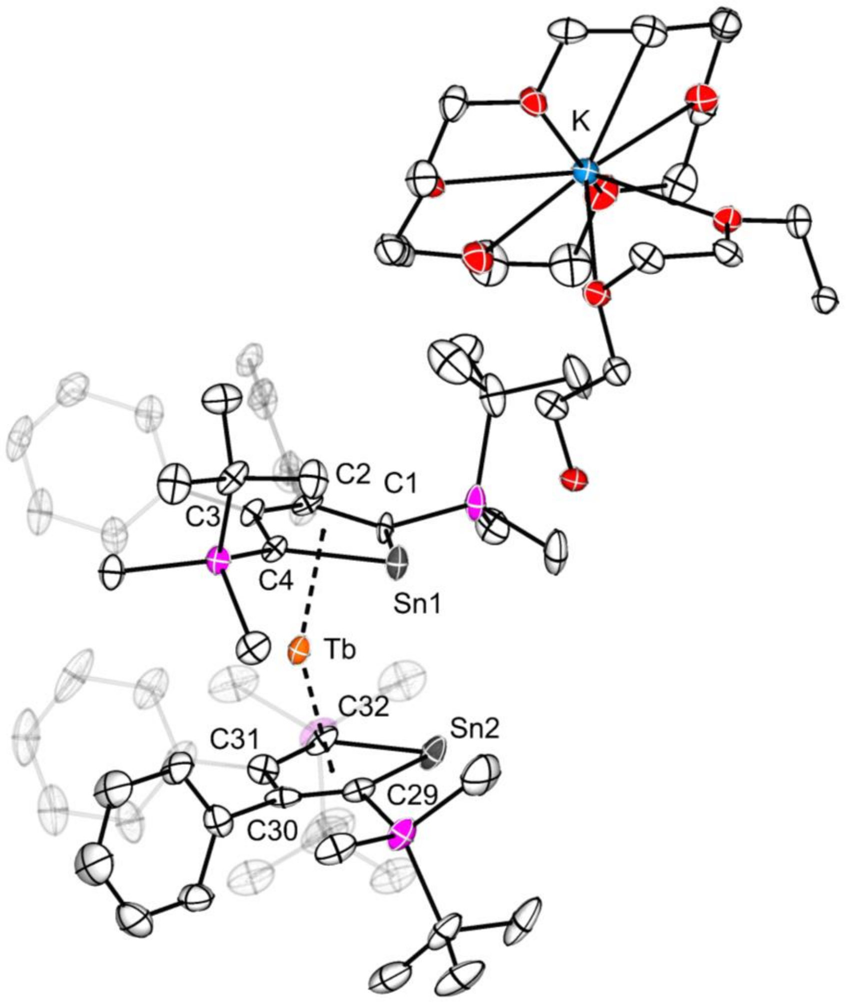

Supplementary Figure 13 Molecular structure of the complex **3-Tb** (crystallized from benzene/THF) in the solid state with thermal ellipsoids at 50% level. All hydrogen atoms and non-coordinating solvent molecules are omitted for clarity. Selected bond distances [Å] and angles [°]: Sn1–C1 2.161(6), Sn1–C4 2.189(4), C1–C2 1.415(9), C2–C3 1.462(8), Sn2–C29 2.161(6), Sn2–C32 2.160(6), C29–C30 1.419(8), C30–C31 1.456(8), C31–C32 1.441(9), Tb–Ct1 2.3432, Tb–Ct2 2.3391; C1–Sn1–C4 79.9(2), C1–C2–C3 117.9(6), C2–C3–C4 118.1(5), C3–C4–Sn1 110.8(4), C29–Sn2–C32 80.0(2), C29–C30–C31 117.3(5), C30–C31–C32 117.7(5), C31–C32–Sn2 111.1(4), Ct1–Tb–Ct2 155.3.

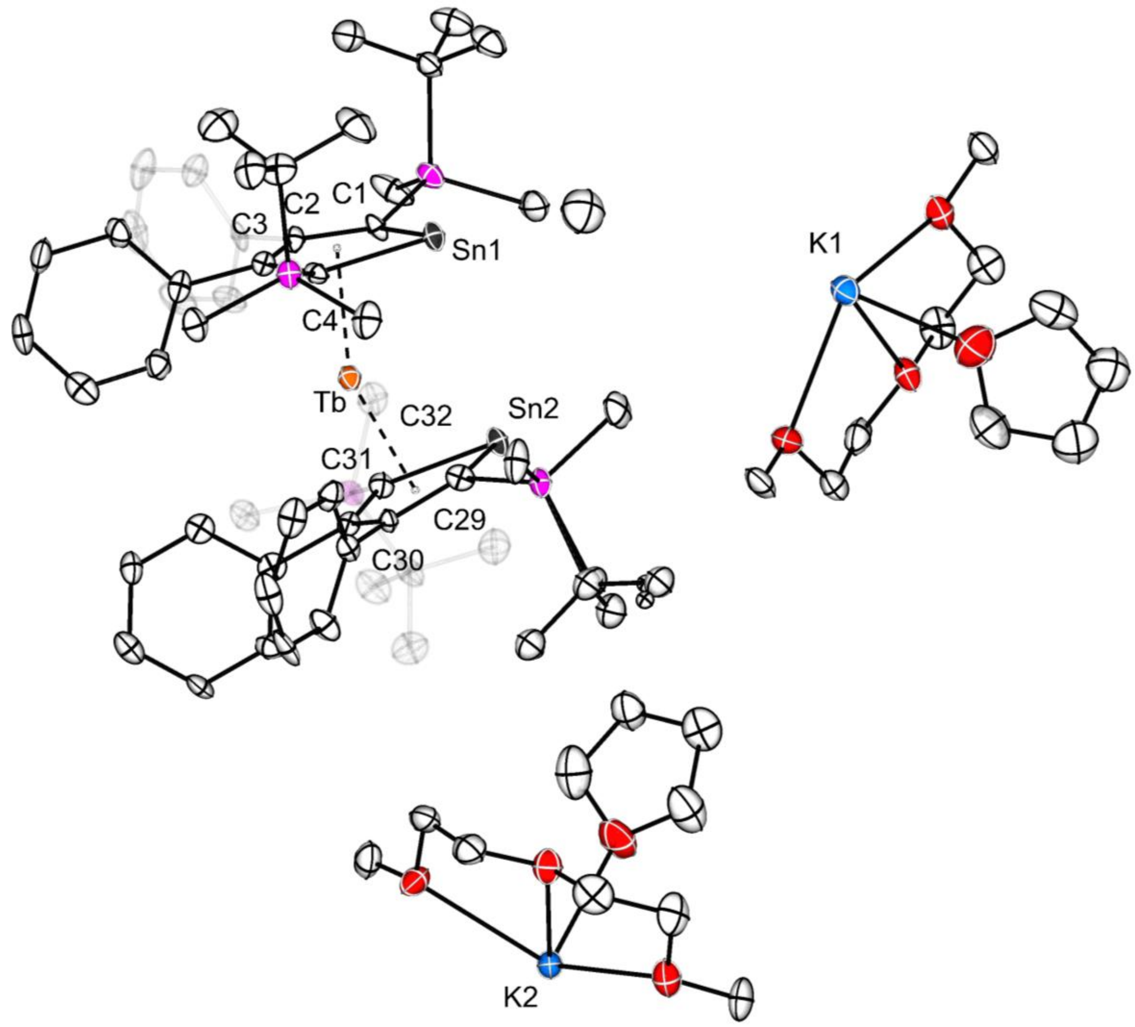

Supplementary Figure 14 Molecular structure of the complex **3-Tb** (crystallized from THF/*n*-hexane mixture) in the solid state with thermal ellipsoids at 50% level. All hydrogen atoms and non-coordinating solvent molecules are omitted for clarity. Selected bond distances [Å] and angles [°]: Sn1–C1 2.185(5), Sn1–C4 2.183(6), C1–C2 1.426(8), C2–C3 1.431(7), Sn2–C29 2.177(5), Sn2–C32 2.166(5), C29–C30 1.448(8), C30–C31 1.437(7), C31–C32 1.434(7), Tb–Ct1 2.3439, Tb–Ct2 2.3490; C1–Sn1–C4 79.7(2), C1–C2–C3 118.5(5), C2–C3–C4 118.7(5), C3–C4–Sn1 111.1(4), C29–Sn2–C32 79.8(2), C29–C30–C31 118.1(4), C30–C31–C32 117.8(5), C31–C32–Sn2 112.0(3), Ct1–Tb–Ct2 156.3.

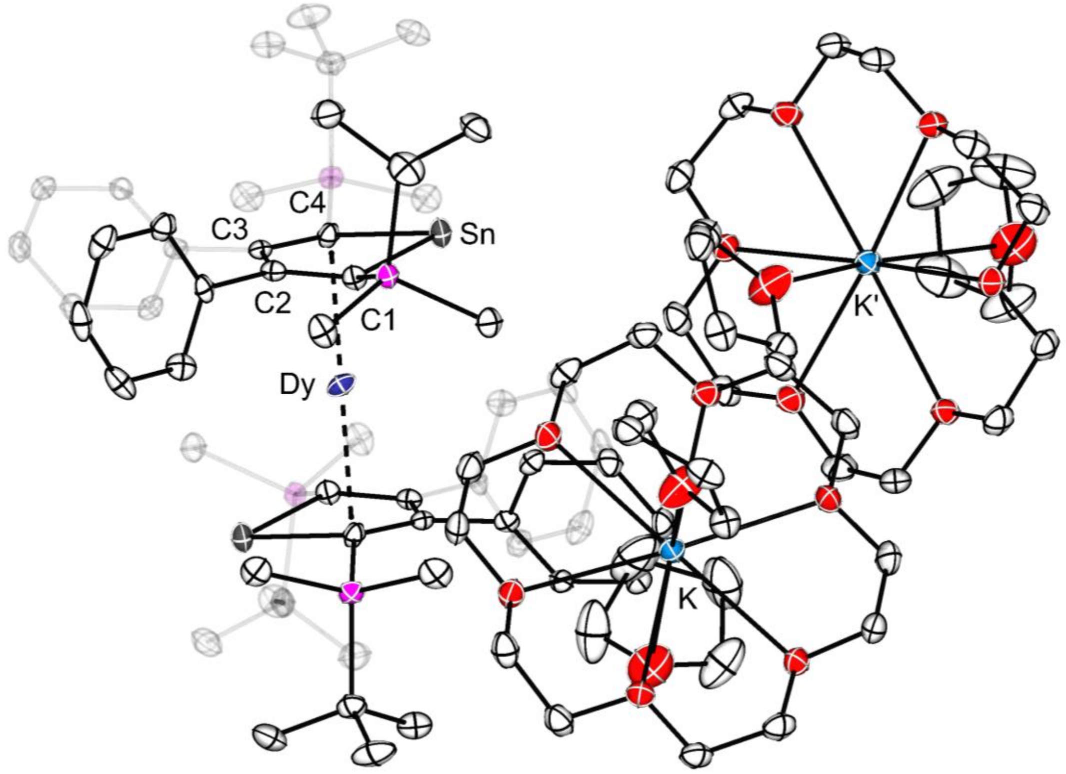

Supplementary Figure 15 Molecular structure of the complex **4-Dy** (crystallized from THF/benzene mixture) in the solid state with thermal ellipsoids at 50% level. All hydrogen atoms and non-coordinating solvent molecules are omitted for clarity. Selected bond distances [Å] and angles [°]: Sn–C1 2.212(2), Sn–C4 2.203(2), C1–C2 1.442(3), C2–C3 1.418(3), C3–C4 1.441(3), Dy–Ct 2.303; C1–Sn–C4 79.33(7), C1–C2–C3 118.9(2), C2–C3–C4 119.2(1), C3–C4–Sn 110.58(13), Ct–Dy–Ct' 180.0.

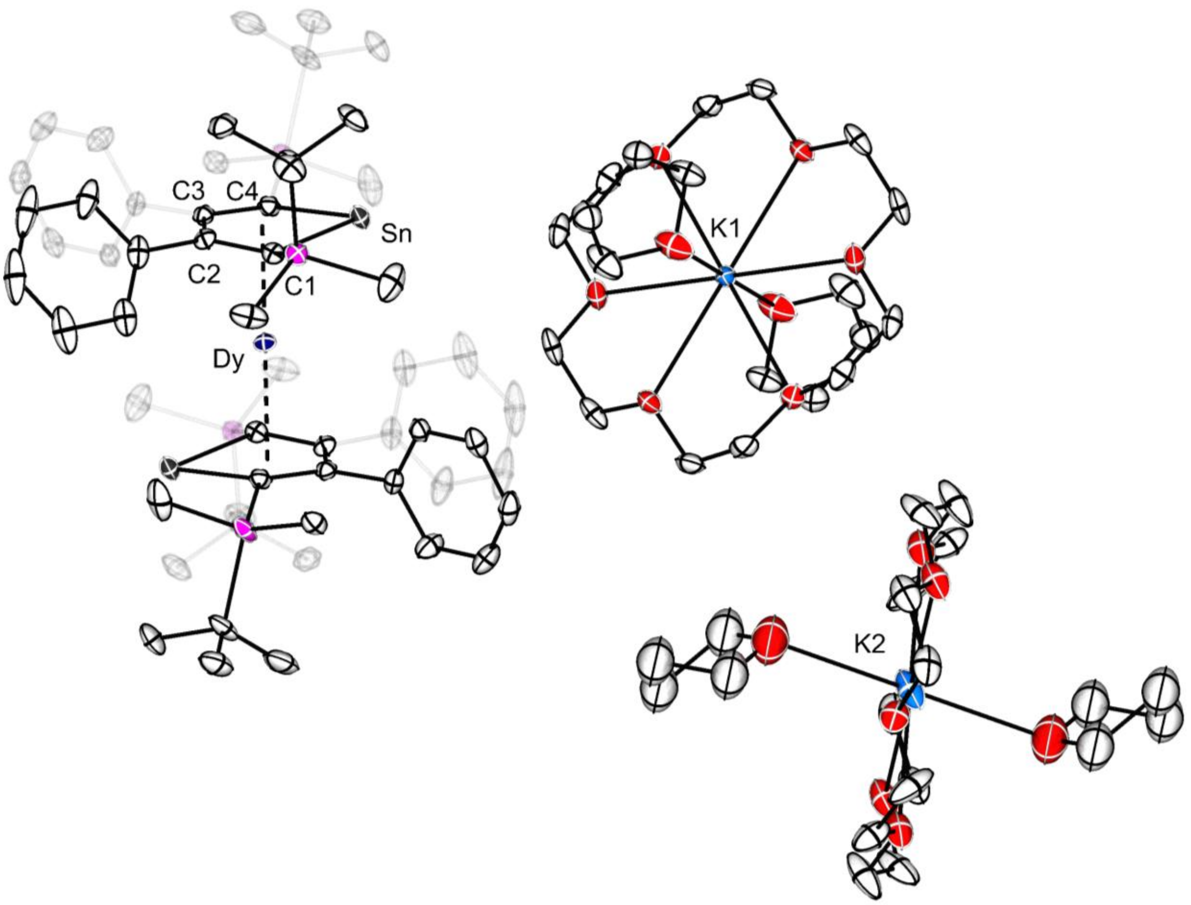

Supplementary Figure 16 Molecular structure of the complex **4-Dy** (crystallized from THF) in the solid state with thermal ellipsoids at 50% level. All hydrogen atoms and non-coordinating solvent molecules are omitted for clarity. Selected bond distances [Å] and angles [°]: Sn–C1 2.206(3), Sn–C4 2.196(3), C1–C2 1.456(4), C2–C3 1.430(4), C3–C4 1.436(4), Dy–Ct 2.2969; C1–Sn–C4 79.24(10), C1–C2–C3 118.2(2), C2–C3–C4 118.7(2), C3–C4–Sn 111.6(2), Ct–Dy–Ct' 180.0.

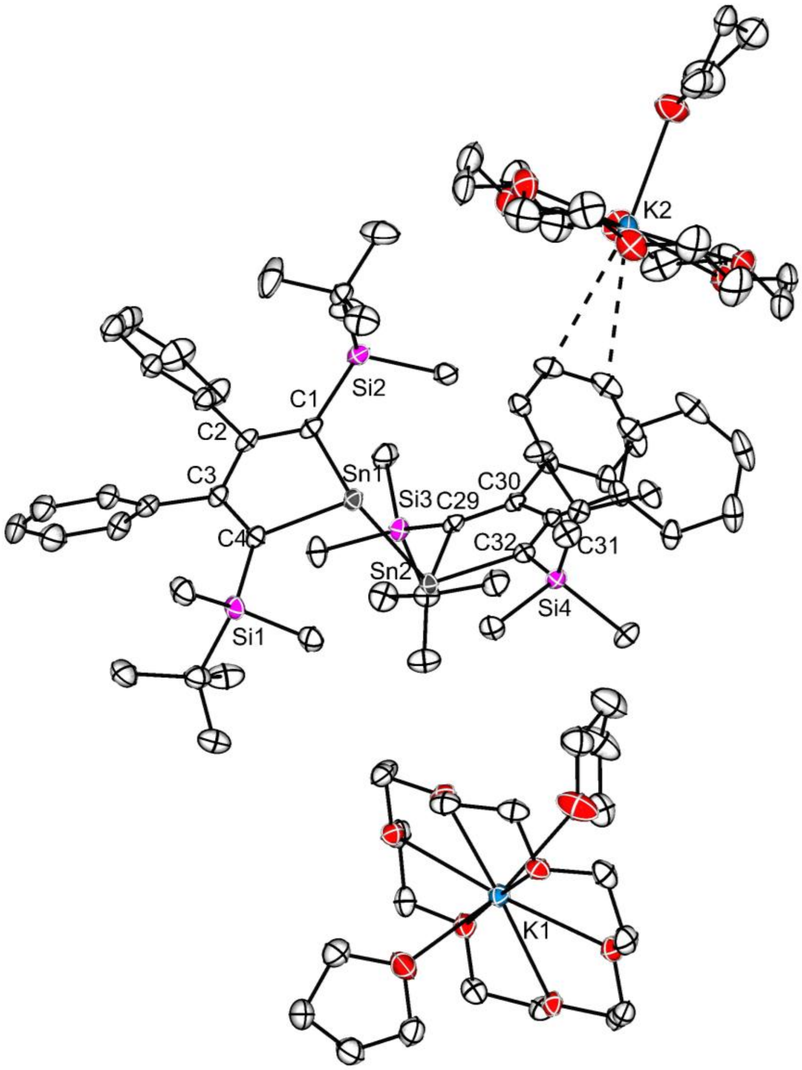

Supplementary Figure 17 Molecular structure of the complex **5** in the solid state with thermal ellipsoids at 50% level. All hydrogen atoms are omitted for clarity. Selected bond distances [Å] and angles [°]: Sn1–Sn2 2.9231(2), Sn1–C1 2.189(2), C1–C2 1.383(3), C2–C3 1.476(3), C3–C4 1.373(3), Sn1–C4 2.190(2), Sn2–C29 2.200(2), C29–C30 1.372(3), C30–C31 1.480(3), C31–C32 1.375(3), Sn2–C32 2.185(2); C1–Sn1–C4 81.43(8), C29–Sn2–C32 80.87(7).

V. UV/vis spectra

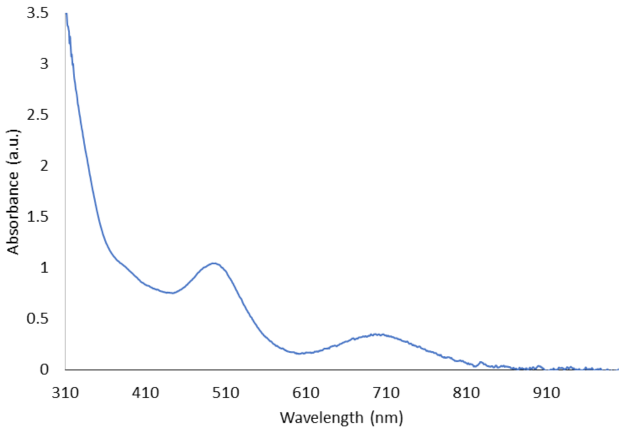

Supplementary Figure 18 UV/vis absorption spectrum of complex **1** recorded in THF.

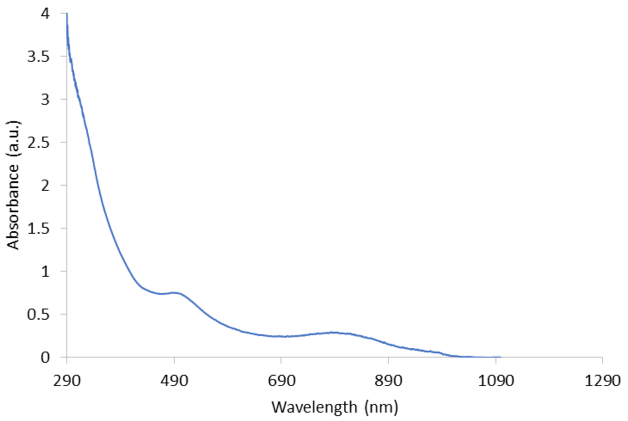

Supplementary Figure 19 UV/vis absorption spectrum of complex **2-Tb** recorded in THF.

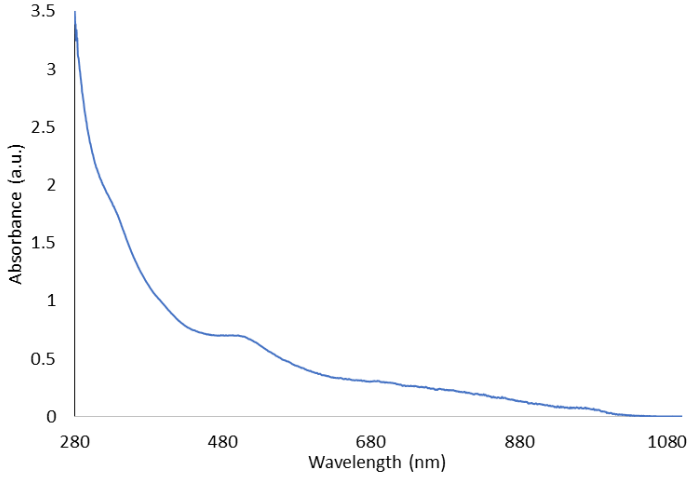

Supplementary Figure 20 UV/vis absorption spectrum of complex **2-Dy** recorded in THF.

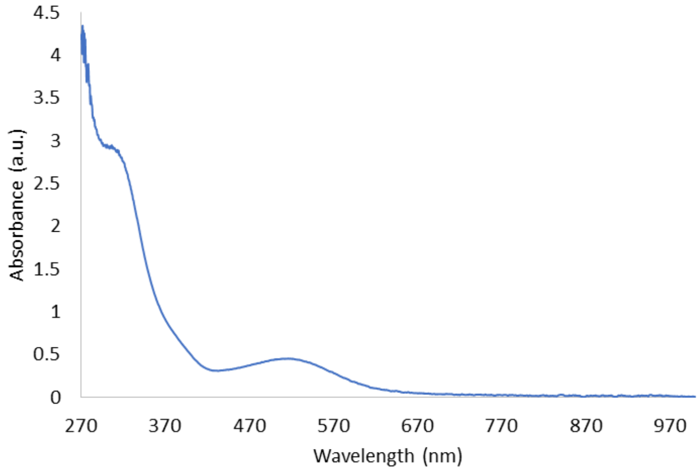

Supplementary Figure 21 UV/vis absorption spectrum of complex **3-Tb** recorded in THF.

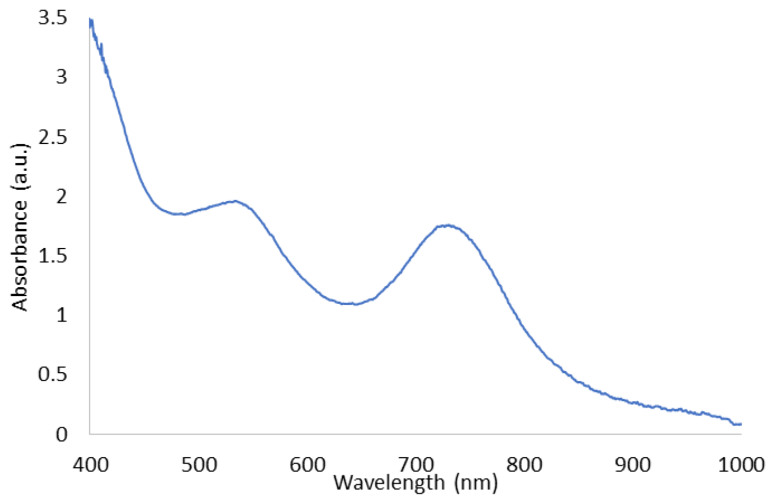

Supplementary Figure 22 UV/vis absorption spectrum of complex **4-Dy** recorded in THF.

VI. PXRD data of 4-Dy

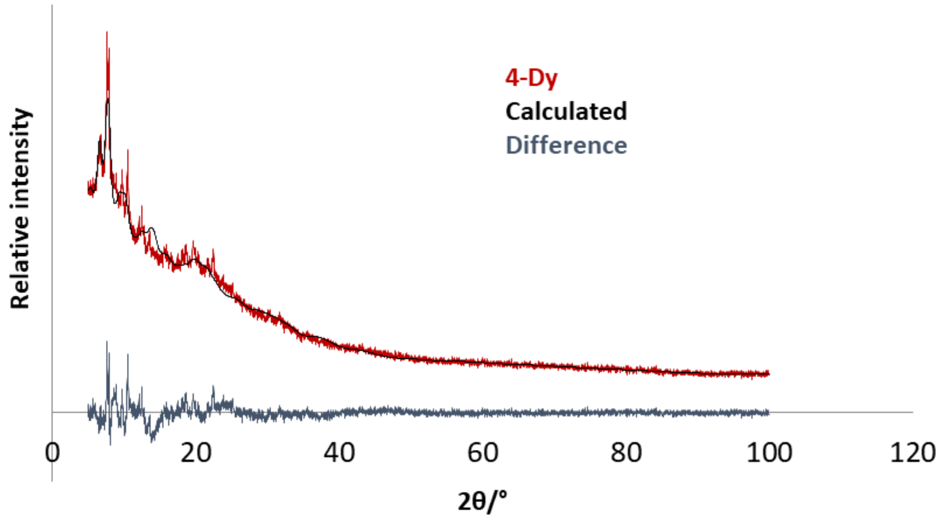

Supplementary Figure 23 Rietveld refinement of diffraction patterns of **4-Dy** with experimental powder diffractogram (red), Rietveld refinement (black), and difference curve (blue).

VII. Magnetism

VII.1 General remarks

The magnetic measurements were carried out on polycrystalline samples of the respective compounds. The finely ground samples were sealed in a glass tube with a small amount of eicosane to avoid movement during the measurements. Magnetic susceptibility measurements were collected using Quantum Design MPMS®3 and MPMS-XL SQUID magnetometers on polycrystalline material in the temperature range 2 – 300 K under an applied DC magnetic field (*H*) ranging from 0 to 0.5 T. Magnetization as a function of applied field was investigated in a MPMS®3 magnetometer in the field and temperature ranges of ±7 T and 1.8–60 K, respectively, at a sweep rate varying from 70 mT/s down to 2 mT/s. The AC data were collected in an MPMS-XL SQUID magnetometer using an oscillating amplitude of 6 Oe and frequencies between 0.1 and 1512 Hz. DC data were corrected for diamagnetic contributions from eicosane and core diamagnetism employing Pascal's constants. DC fields between 0 and 0.5 T were employed during the AC data collection.

VII.2 DC Magnetic studies

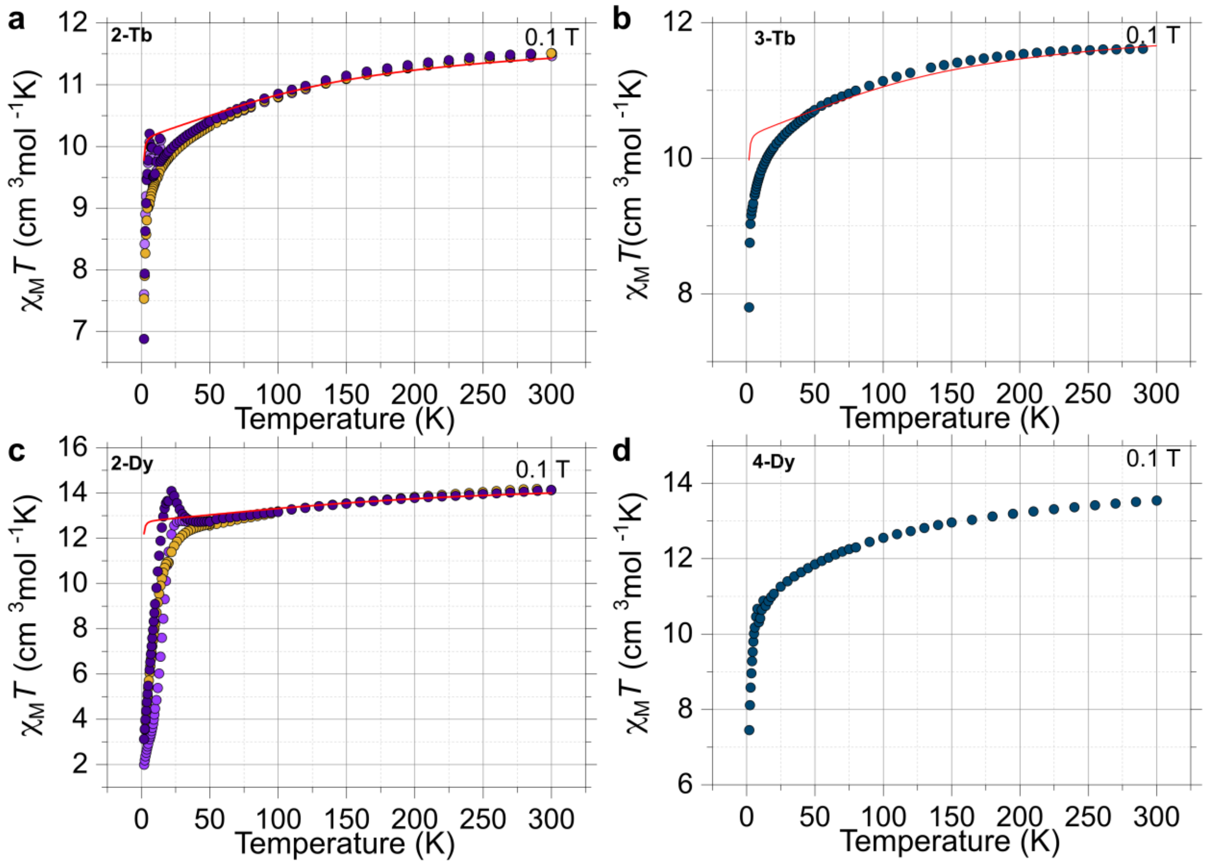

Supplementary Figure 24 Temperature dependence of the magnetic susceptibility (χ_MT) for complexes (**a**) **2-Tb**, (**b**) **3-Tb**, (**c**) **2-Dy**, and (**d**) **4-Dy** with an applied DC field of 0.1 T. The different traces for **2-Tb** and **2-Dy** correspond to the Field-Cooled Zero-Field-Cooled traces. The solid red lines in panels **a**, **b**, and **c** are the CASSCF calculated $\chi_M T(T)$ traces.

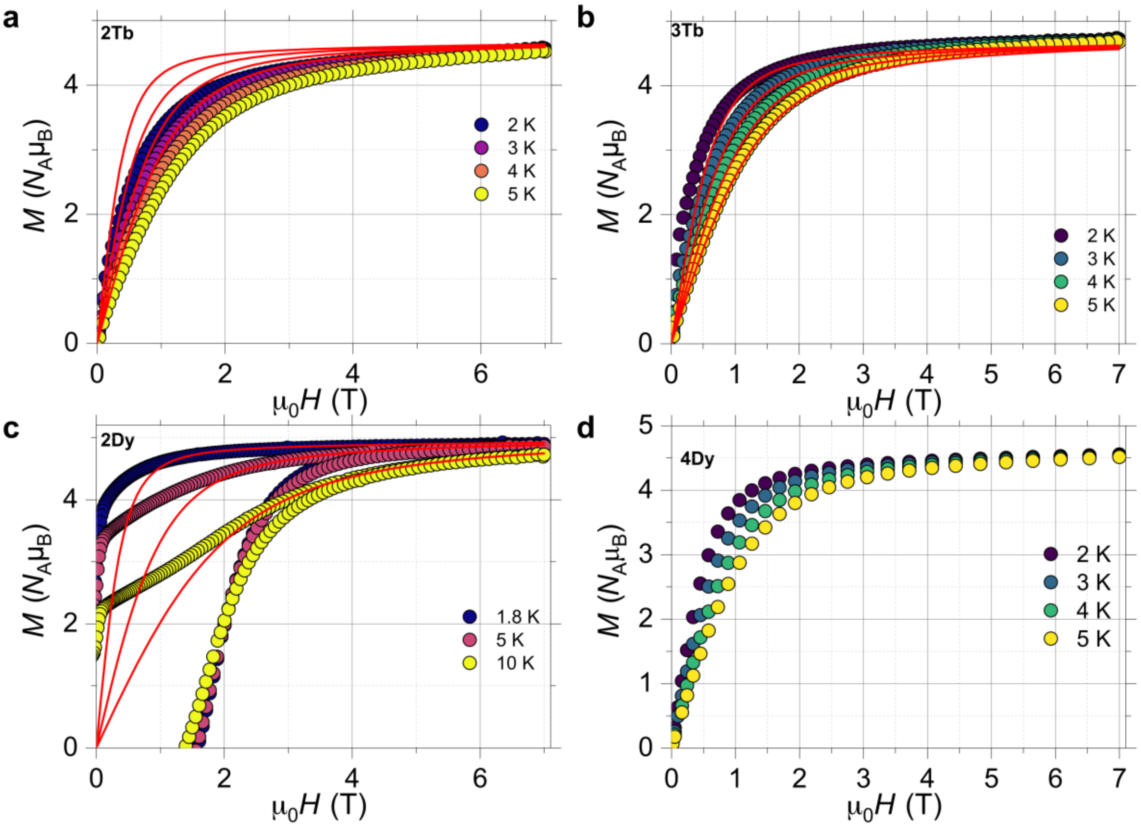

Supplementary Figure 25 *M*(*H*) experimental data for (**a**) **2-Tb**, (**b**) **3-Tb**, (**c**) **2-Dy,** and (**d**) **4-Dy**. The solid traces in panels **a**, **b**, and **c** are the CASSCF calculated *M*(*H*) traces.

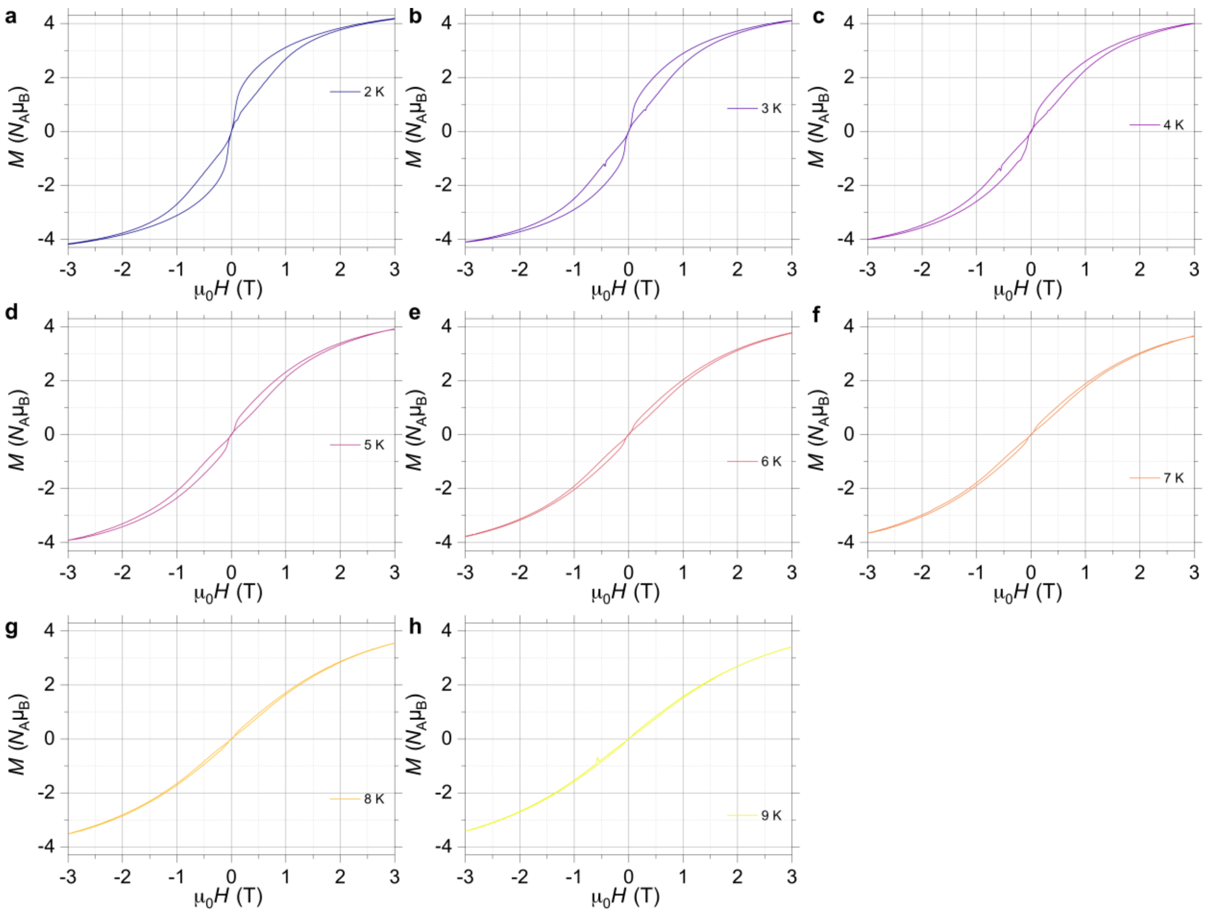

Supplementary Figure 26 Hysteresis *M*(*H*) loops for **2-Tb** at different temperatures, showing opening of the loops up to 9 K at a sweep rate of 5 mT/s.

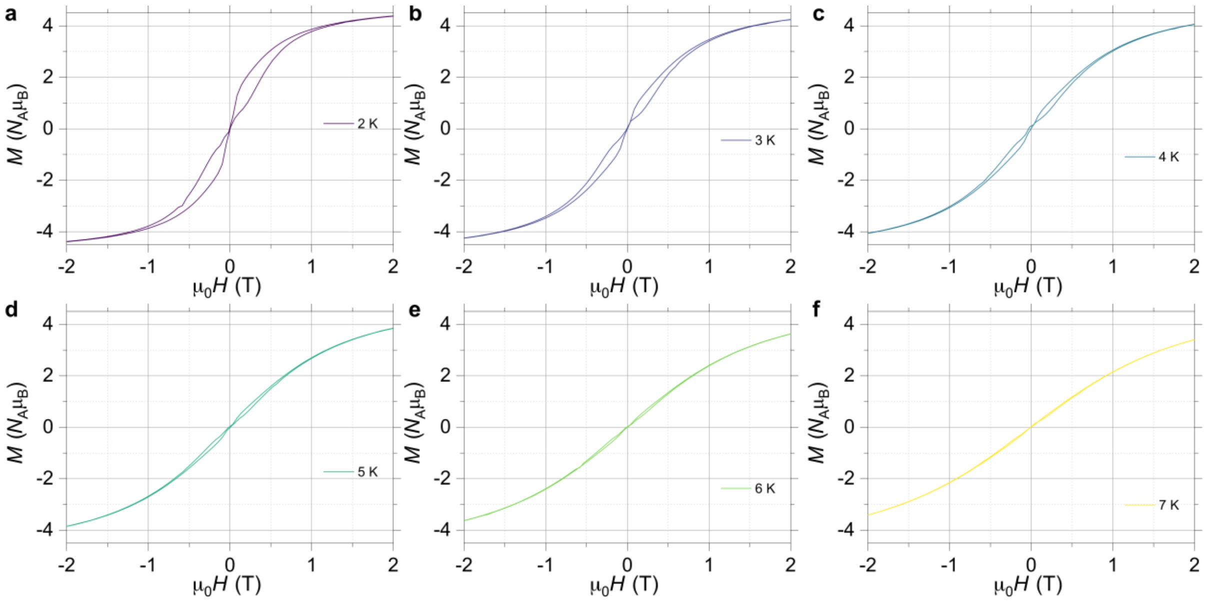

Supplementary Figure 27 Hysteresis *M*(*H*) loops for **3-Tb** at different temperatures, showing opening of the loops up to 7 K at a sweep rate of 5 mT/s.

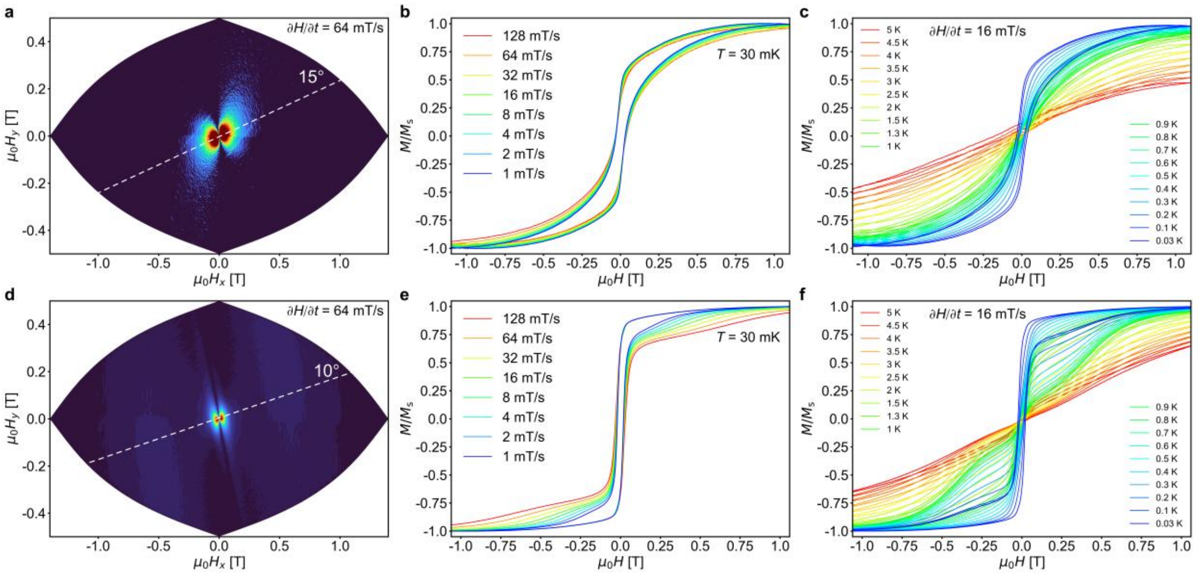

Supplementary Figure 28 (**a**, **d**) angular map μSQUID hysteresis loops at 64 mT/s, revealing angles of 15° and 10° with respect to the x-magnet, for **2-Tb** and **3-Tb**, respectively. (**b**, **e**) sweep rate dependence at 30 mK and (**c**, **f**) temperature dependence study at a fixed sweep rate of 16 mT/s for **2-Tb** and **3-Tb**, respectively. The sweep- and temperature dependence data were collected with the magnetic field along the crystallographic easy axis as revealed in the angular map (**a**, **d**).

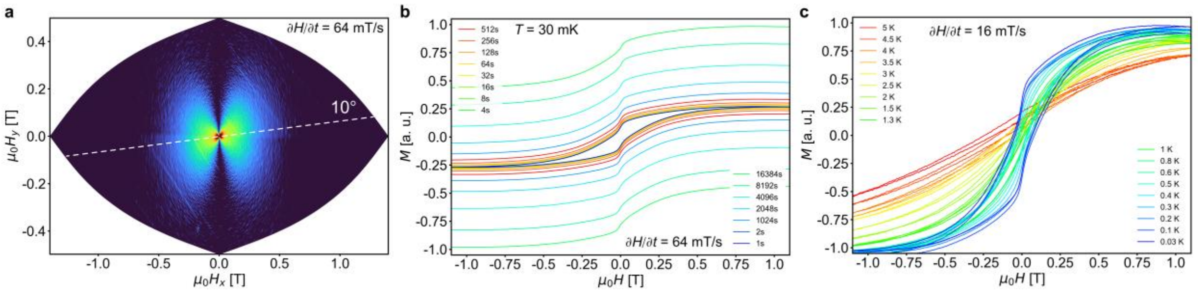

Supplementary Figure 29 μSQUID for **2-Dy**. (**a**) angular map μSQUID hysteresis loops at 64 mT/s, revealing an angle of 10° with respect to the x-magnet. (**b**) waiting time dependence at 30 mK and a fixed sweep rate of 64 mT/s, and (**c**) temperature dependence study at a fixed sweep rate of 16 mT/s. The waiting time- and temperature dependence data were collected with the magnetic field along the crystallographic easy axis as revealed in the angular map (**a**).

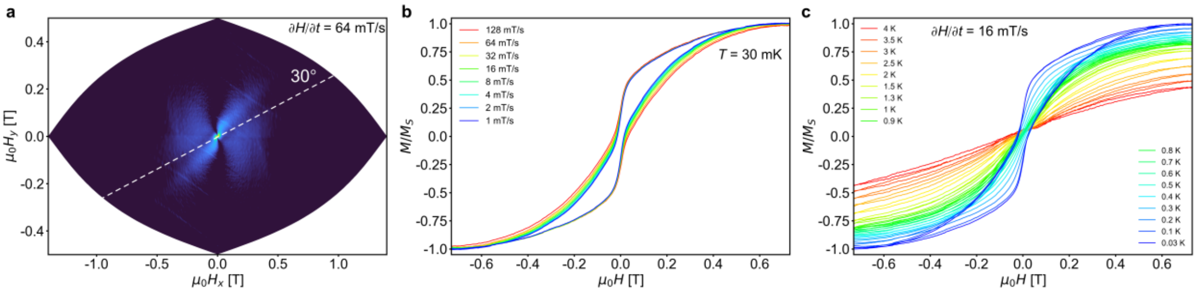

Supplementary Figure 30 (**a**) angular map μSQUID hysteresis loops at 64 mT/s, revealing an angle of 30° with respect to the x-magnet. (**b**) sweep rate dependence at 30 mK and (**c**) temperature dependence at a fixed sweep rate of 16 mT/s. The sweep- and temperature-dependent data were collected with the magnetic field along the crystallographic easy axes, as revealed in the angular map (**a**).

VII.3 AC Magnetic studies

The $\tau(T,H)$ relaxation data were obtained by simultaneously fitting the collected AC ($\chi_M''/\chi_M'(\nu,T)$) to an extended Debye model. The standard deviation of the $\tau(T, H)$ data was calculated taking into account the fitted α parameters and Equation S1:[6]

$$\sigma^2_{\langle \ln \tau \rangle} = \left(\frac{1}{(1-\alpha)^2} - 1\right)\frac{\pi^2}{3} \quad \text{(S1)}$$

The upper and lower limit of the standard deviation of $\tau(T, H)$ was subsequently calculated according to Equation S2: [6]

$$\tau_{\pm} = \exp\left(\langle \ln \tau \rangle \pm \sqrt{\sigma^2_{\langle \ln \tau \rangle}}\right) \quad \text{(S2)}$$

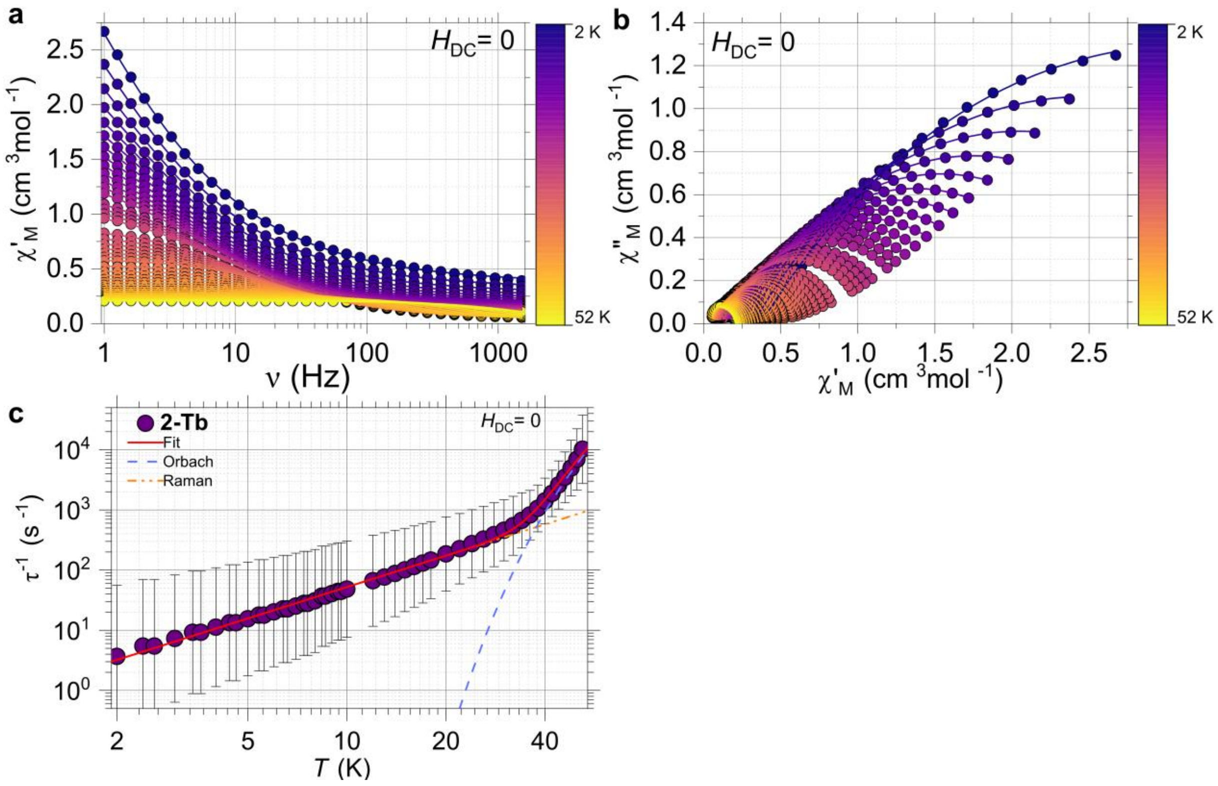

Supplementary Figure 31 (**a**) $\chi'_M(T;\nu)$ for compound **2-Tb** at zero DC field and (**b**) Cole-Cole plot at the same field, both in the temperature range of 2 and 52 K. Solid lines are fits to an extended Debye model. Panel (**c**) is the $\tau(T)$

data, associated errors, fit, and contribution of the Orbach and Raman processes to the overall fit. The error bars represent 1 estimated standard deviation of the distribution of rates. The fit was performed using equation (1) in the main manuscript.

Supplementary Table 2 Fitted parameters for magnetic data of **2-Tb** (H_{DC} = 0), as depicted in Figures 4a,c**,** and Supplementary Figure 31 for a simultaneous fit of $\chi''_M(T;\nu)$ and $\chi'_M(T;\nu)$ and associated errors. The τ errors were obtained with equations S1 and S2.

T (K)	χ_S (cm^3 mol^{-1})	χ_S error	χ_T (cm^3 mol^{-1})	χ_T error	α	α error	τ (s)	$\tau_{\_upper\ limit}$ (s)	$\tau_{\_lower\ limit}$ (s)
2	0.371	0.010	5.930	1.9E-01	0.445	0.009	0.27228	4.12508	0.01797
2.4	0.322	0.007	4.620	9.7E-02	0.420	0.007	0.1837	2.34044	0.01442
2.6	0.322	0.007	4.620	9.7E-02	0.420	0.007	0.1837	2.34044	0.01442
3	0.283	0.006	3.804	6.3E-02	0.403	0.006	0.13737	1.56772	0.01204
3.4	0.255	0.005	3.244	4.2E-02	0.388	0.006	0.10823	1.12911	0.01037
3.6	0.255	0.005	3.244	4.2E-02	0.388	0.006	0.10823	1.12911	0.01037
4	0.232	0.004	2.839	2.9E-02	0.377	0.005	0.08907	0.86706	0.00915
4.4	0.213	0.003	2.526	1.9E-02	0.367	0.004	0.07505	0.69175	0.00814
4.6	0.213	0.003	2.526	1.9E-02	0.367	0.004	0.07505	0.69175	0.00814
5	0.196	0.003	2.280	1.7E-02	0.360	0.004	0.0647	0.57067	0.00734
5.4	0.185	0.002	2.072	1.3E-02	0.351	0.004	0.05631	0.47181	0.00672
5.6	0.185	0.002	2.072	1.3E-02	0.351	0.004	0.05631	0.47181	0.00672
6	0.173	0.002	1.905	1.1E-02	0.345	0.004	0.0498	0.40382	0.00614
6.4	0.164	0.002	1.759	9.2E-03	0.338	0.004	0.04429	0.34464	0.00569
6.6	0.164	0.002	1.759	9.2E-03	0.338	0.004	0.04429	0.34464	0.00569
7	0.155	0.002	1.637	9.1E-03	0.334	0.004	0.03982	0.30301	0.00523
7.4	0.147	0.002	1.528	7.4E-03	0.327	0.004	0.03589	0.26382	0.00488
7.6	0.147	0.002	1.528	7.4E-03	0.327	0.004	0.03589	0.26382	0.00488
8	0.141	0.002	1.435	7.8E-03	0.323	0.004	0.03261	0.23458	0.00453
8.4	0.129	0.002	1.276	6.9E-03	0.313	0.005	0.02713	0.18486	0.00398
8.6	0.129	0.002	1.276	6.9E-03	0.313	0.005	0.02713	0.18486	0.00398
9	0.124	0.002	1.204	6.4E-03	0.308	0.005	0.02472	0.16387	0.00373
9.4	0.120	0.002	1.139	6.2E-03	0.302	0.005	0.02263	0.14554	0.00352
9.6	0.120	0.002	1.139	6.2E-03	0.302	0.005	0.02263	0.14554	0.00352
10	0.115	0.002	1.082	6.0E-03	0.298	0.005	0.02072	0.13067	0.00329
12	0.101	0.003	0.899	5.3E-03	0.279	0.007	0.01508	0.08615	0.00264
13	0.097	0.003	0.830	5.2E-03	0.270	0.007	0.01309	0.07143	0.00240
14	0.092	0.003	0.771	4.9E-03	0.261	0.008	0.01139	0.05947	0.00218
15	0.089	0.003	0.717	4.3E-03	0.248	0.008	0.00996	0.04893	0.00203
16	0.084	0.003	0.672	4.2E-03	0.241	0.009	0.00873	0.04139	0.00184
17	0.080	0.003	0.632	4.0E-03	0.234	0.009	0.00769	0.03522	0.00168
18	0.079	0.003	0.596	3.6E-03	0.224	0.009	0.00683	0.02983	0.00156
20	0.071	0.003	0.536	3.3E-03	0.212	0.010	0.0054	0.02231	0.00131

22	0.068	0.003	0.486	2.7E-03	0.197	0.010	0.00442	0.01699	0.00115
24	0.064	0.003	0.445	2.4E-03	0.188	0.010	0.00366	0.01347	0.00099
26	0.061	0.002	0.411	2.1E-03	0.178	0.010	0.00307	0.01081	0.00087
28	0.057	0.002	0.381	1.8E-03	0.171	0.009	0.00258	0.00877	0.00076
30	0.055	0.002	0.356	1.6E-03	0.160	0.010	0.00218	0.00702	0.00068
32	0.052	0.002	0.334	1.6E-03	0.151	0.011	0.00184	0.00569	0.00060
34	0.050	0.002	0.314	1.2E-03	0.136	0.009	0.0015	0.00431	0.00052
36	0.050	0.002	0.296	9.3E-04	0.114	0.008	0.00122	0.00315	0.00047
38	0.047	0.001	0.281	6.9E-04	0.103	0.007	9.46E-04	0.00232	0.00039
40	0.042	0.001	0.267	5.8E-04	0.100	0.006	7.11E-04	0.00171	0.00030
42	0.039	0.002	0.255	5.9E-04	0.102	0.007	5.33E-04	0.00129	0.00022
44	0.035	0.002	0.243	5.5E-04	0.108	0.008	3.85E-04	0.00097	0.00015
46	0.031	0.003	0.233	5.6E-04	0.119	0.009	2.88E-04	0.00076	0.00011
48	0.024	0.003	0.224	4.6E-04	0.138	0.009	2.02E-04	0.00059	0.00007
50	0.017	0.005	0.215	5.4E-04	0.160	0.013	1.45E-04	0.00047	0.00004
52	0.004	0.009	0.208	5.8E-04	0.190	0.016	9.82E-05	0.00036	0.00003

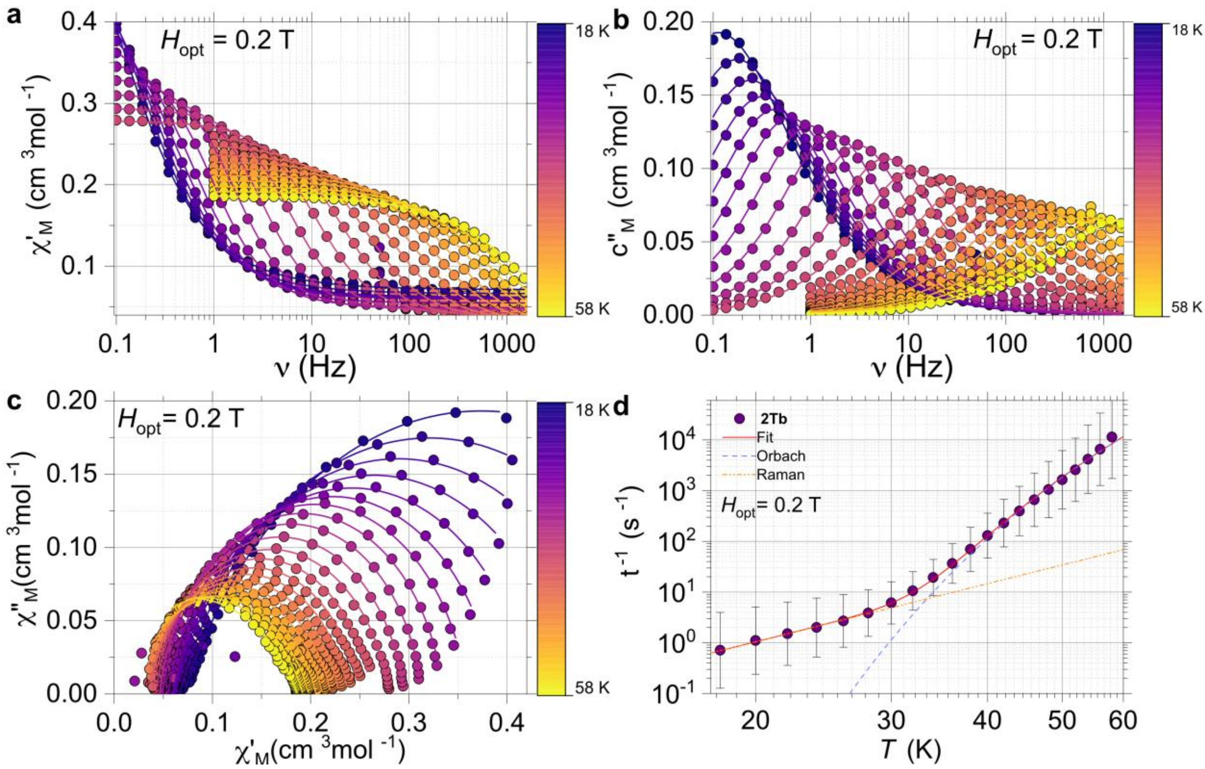

Supplementary Figure 32 AC dynamic susceptibility for **2-Tb** with H_{opt} = 0.2 T. (**a**) $\chi'_M(T;\nu)$, (**b**) $\chi''_M(T;\nu)$, (**c**) Cole-Cole plot at the same field, all in the temperature range of 18 and 58 K. Solid lines are fits to an extended Debye model. Panel (**d**) is the $\tau(T)$ data, associated errors, fit, and contribution of the Raman and Orbach processes to the overall fit. The error bars represent 1 estimated standard deviation of the distribution of rates. The fit was performed using equation (1) in the main manuscript.

Supplementary Table 3 Fitted parameters for magnetic data of **2-Tb** (H_{opt} = 0.2 T), as depicted in Figures 4b,c, and Supplementary Figure 32 for a simultaneous fit of $\chi''_M(T;\nu)$ and $\chi'_M(T;\nu)$ and associated errors. The τ errors were obtained with equations S1 and S2.

T (K)	χ_S (cm^3 mol^{-1})	χ_S error	χ_T (cm^3 mol^{-1})	χ_T error	α	α err.	τ (s)	$\tau_{\_upper\ limit}$ (s)	$\tau_{\_lower\ limit}$ (s)
18	0.071	0.003	0.674	0.043	0.275	0.0275	1.4067	7.88629	0.25090
20	0.066	0.005	0.575	0.048	0.235	0.0473	0.9044	4.15603	0.19682
22	0.062	0.002	0.515	0.017	0.217	0.0230	0.6611	2.79072	0.15663
24	0.061	0.003	0.467	0.015	0.195	0.0249	0.5001	1.90533	0.13125
26	0.060	0.004	0.424	0.018	0.165	0.0394	0.3713	1.22412	0.11262
28	0.055	0.002	0.388	0.007	0.136	0.0189	0.2593	0.74630	0.09012
30	0.049	0.002	0.359	0.006	0.116	0.0198	0.1635	0.42578	0.06275
32	0.047	0.002	0.334	0.004	0.099	0.0161	0.0946	0.22644	0.03952
34	0.046	0.001	0.312	0.002	0.089	0.0094	0.0517	0.11780	0.02271
36	0.042	0.001	0.294	0.001	0.104	0.0042	0.0273	0.06719	0.01108
38	0.039	0.001	0.279	0.001	0.125	0.0090	0.0143	0.03921	0.00524
40	0.036	0.001	0.261	0.002	0.127	0.0106	0.0077	0.02110	0.00279
42	0.034	0.002	0.249	0.002	0.141	0.0135	0.0044	0.01286	0.00148
44	0.031	0.002	0.237	0.002	0.149	0.0154	0.0025	0.00776	0.00082
46	0.027	0.003	0.228	0.002	0.169	0.0175	0.0015	0.00511	0.00045
48	0.025	0.004	0.220	0.002	0.182	0.0191	0.0010	0.00341	0.00027
50	0.021	0.005	0.212	0.002	0.192	0.0230	0.0006	0.00228	0.00016
52	0.016	0.006	0.205	0.001	0.217	0.0229	0.0004	0.00164	0.00009
54	0.001	0.009	0.198	0.002	0.241	0.0270	0.0002	0.00113	0.00005
56	-0.008	0.010	0.192	0.001	0.259	0.0230	0.0002	0.00079	0.00003
58	-0.029	0.015	0.186	0.001	0.306	0.0234	0.0001	0.00058	0.00001

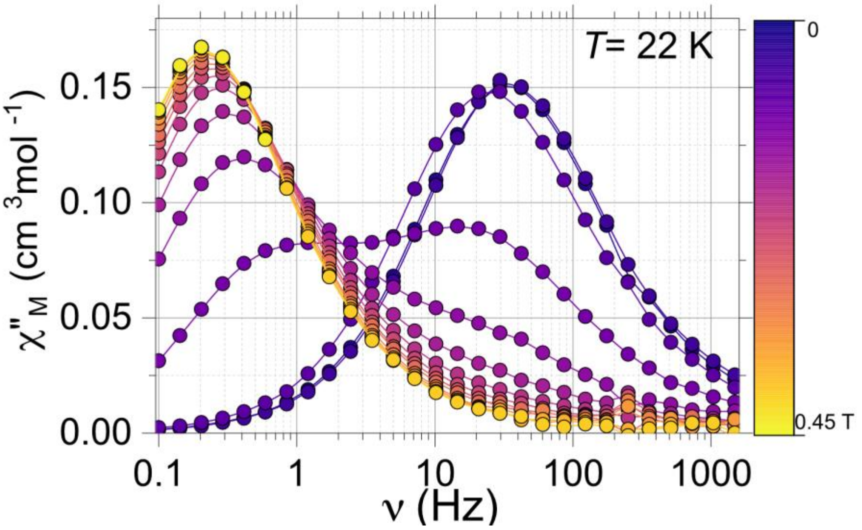

Supplementary Figure 33 Field dependence of χ″$_M$(ν) for **2-Tb** at a fixed temperature of 22 K.

Supplementary Table 4 Fitted parameters for magnetic data field dependence of **2-Tb** (T = 22 K), as depicted in Figure 4d for a simultaneous fit of $\chi''_M(T;\nu)$ and $\chi'_M(T;\nu)$ and associated errors. The τ errors were obtained with equations S1 and S2.

z	χ_S (cm^3 mol^{-1})	χ_S error	χ_T (cm^3 mol^{-1})	χ_T error	α	α err.	τ (s)	$\tau_{\_upper\ limit}$ (s)	$\tau_{\_lower\ limit}$ (s)
0.000	0.481	0.001	0.071	0.002	0.181	0.006	0.0044	0.0155	0.0012
0.010	0.451	0.002	0.183	0.002	0.208	0.012	0.0036	0.0144	0.0009
0.020	0.481	0.001	0.070	0.002	0.180	0.006	0.0042	0.0148	0.0012
0.030	0.451	0.001	0.184	0.001	0.198	0.006	0.0045	0.0172	0.0012
0.040	0.482	0.001	0.069	0.002	0.198	0.006	0.0058	0.0224	0.0015
0.050	0.444	0.002	0.175	0.001	0.339	0.008	0.0079	0.0616	0.0010
0.060	0.491	0.003	0.231	0.009	0.273	0.023	0.1512	0.8367	0.0273
0.060	0.373	0.004	0.055	0.002	0.343	0.009	0.0106	0.0853	0.0013
0.070	0.458	0.003	0.258	0.004	0.266	0.018	0.2651	1.4202	0.0495
0.070	0.332	0.013	0.174	0.005	0.370	0.052	0.0118	0.1096	0.0013
0.080	0.507	0.005	0.140	0.005	0.264	0.015	0.3600	1.9138	0.0677
0.090	0.462	0.002	0.221	0.002	0.234	0.011	0.4243	1.9399	0.0928
0.100	0.503	0.004	0.110	0.003	0.213	0.010	0.4804	1.9943	0.1157
0.125	0.505	0.004	0.088	0.002	0.204	0.008	0.5543	2.1978	0.1398
0.150	0.530	0.008	0.064	0.001	0.257	0.010	0.6287	3.2260	0.1225
0.175	0.521	0.007	0.064	0.001	0.229	0.009	0.6478	2.9019	0.1446
0.200	0.518	0.005	0.062	0.001	0.220	0.007	0.6663	2.8531	0.1556
0.250	0.514	0.006	0.060	0.001	0.206	0.008	0.6968	2.7989	0.1735
0.300	0.509	0.006	0.061	0.001	0.192	0.008	0.7130	2.6746	0.1901
0.350	0.508	0.004	0.060	0.001	0.188	0.005	0.7286	2.6782	0.1982
0.400	0.506	0.004	0.061	0.001	0.182	0.005	0.7373	2.6415	0.2058
0.450	0.490	0.003	0.073	0.002	0.136	0.007	0.7350	2.1146	0.2555

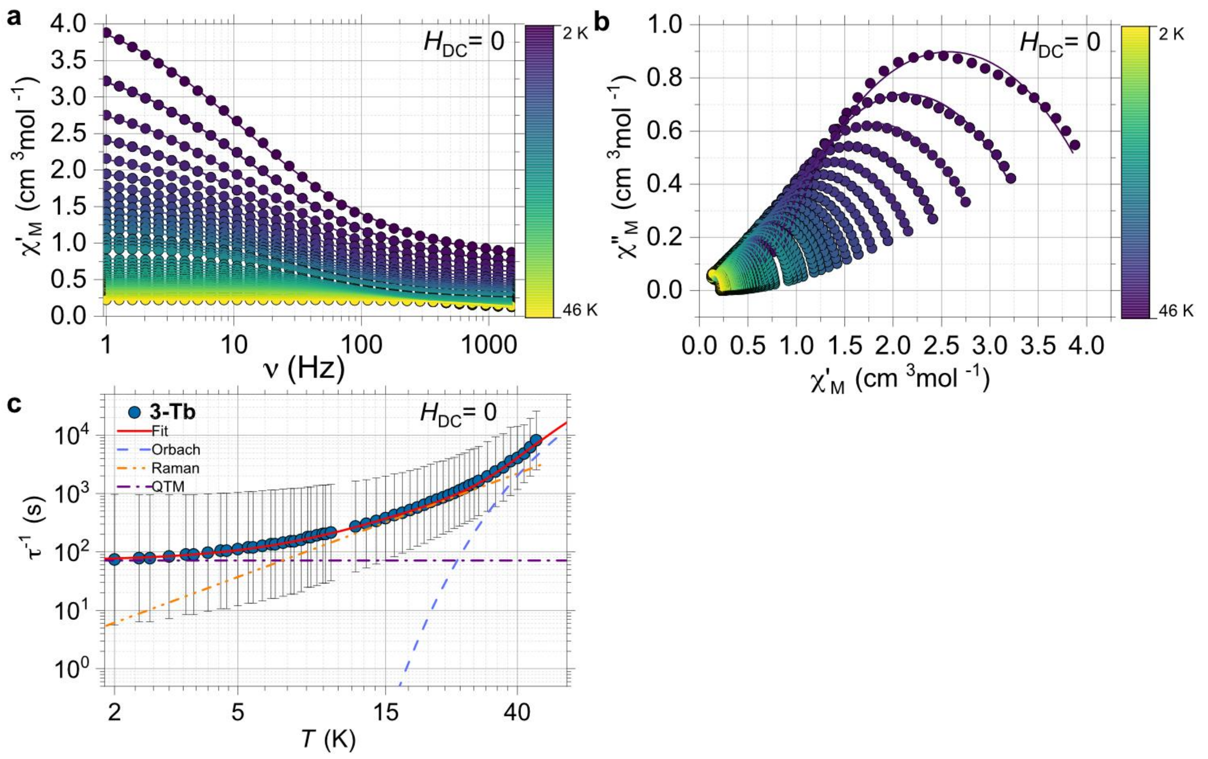

Supplementary Figure 34 AC dynamic susceptibility for **3-Tb** with H_{opt} = 0 T. (**a**) $\chi'_M(T;\nu)$, (**b**) Cole-Cole plot at the same field, all in the temperature range of 2 and 46 K. Solid lines are fits to an extended Debye model. Panel (**c**) is the $\tau(T)$ data, associated errors, fit, and contribution of the Raman and Orbach processes to the overall fit. The error bars represent 1 estimated standard deviation of the distribution of rates. The fit was performed using equation (1) in the main manuscript.

Supplementary Table 5 Fitted parameters for magnetic data of **3-Tb** (H_{DC} = 0), as depicted in Figure 5a,c, and Supplementary Figure 34 for a simultaneous fit of $\chi''_M(T;\nu)$ and $\chi'_M(T;\nu)$ and associated errors. The τ errors were obtained with equations S1 and S2.

T (K)	χ_S (cm^3 mol^{-1})	χ_S error	χ_T (cm^3 mol^{-1})	χ_T error	α	α err.	τ (s)	$\tau_{\_upper\ limit}$ (s)	$\tau_{\_lower\ limit}$ (s)
2	0.7296	0.0214	4.4225	0.0441	0.424	0.010	0.01354	0.177131	0.001035
2.4	0.6500	0.0145	3.6278	0.0288	0.414	0.008	0.01283	0.157233	0.001047
2.6	0.6500	0.0145	3.6278	0.0288	0.414	0.008	0.01283	0.157233	0.001047
3	0.5869	0.0103	3.0648	0.0195	0.403	0.007	0.01198	0.137102	0.001047
3.4	0.5402	0.0070	2.6581	0.0127	0.392	0.006	0.01114	0.118732	0.001045
3.6	0.5402	0.0070	2.6581	0.0127	0.392	0.006	0.01114	0.118732	0.001045
4	0.4978	0.0055	2.3528	0.0095	0.383	0.005	0.01035	0.104598	0.001024
4.4	0.4591	0.0044	2.1133	0.0073	0.378	0.004	0.00956	0.093553	0.000977
4.6	0.4591	0.0044	2.1133	0.0073	0.378	0.004	0.00956	0.093553	0.000977
5	0.4300	0.0040	1.9185	0.0063	0.370	0.004	0.00897	0.083752	0.000961
5.4	0.4043	0.0038	1.7580	0.0057	0.363	0.004	0.00841	0.075725	0.000934
5.6	0.4043	0.0038	1.7580	0.0057	0.363	0.004	0.00841	0.075725	0.000934
6	0.3815	0.0036	1.6240	0.0052	0.359	0.005	0.00789	0.069085	0.000901
6.4	0.3618	0.0038	1.5088	0.0054	0.353	0.005	0.00742	0.063031	0.000873
6.6	0.3618	0.0038	1.5088	0.0054	0.353	0.005	0.00742	0.063031	0.000873
7	0.3424	0.0039	1.4099	0.0053	0.349	0.006	0.00697	0.057615	0.000843
7.4	0.3275	0.0041	1.3224	0.0053	0.343	0.006	0.00658	0.052637	0.000823
7.6	0.3275	0.0041	1.3224	0.0053	0.343	0.006	0.00658	0.052637	0.000823
8	0.3134	0.0040	1.2452	0.0051	0.336	0.007	0.00622	0.048025	0.000806
8.4	0.2904	0.0043	1.1155	0.0052	0.324	0.008	0.00560	0.040457	0.000775
8.6	0.2904	0.0043	1.1155	0.0052	0.324	0.008	0.00560	0.040457	0.000775
9	0.2764	0.0045	1.0573	0.0051	0.322	0.008	0.00522	0.037302	0.000730
9.4	0.2673	0.0043	1.0039	0.0048	0.315	0.008	0.00494	0.033984	0.000718
9.6	0.2673	0.0043	1.0039	0.0048	0.315	0.008	0.00494	0.033984	0.000718
10	0.2565	0.0046	0.9559	0.0049	0.310	0.009	0.00465	0.031168	0.000694
12	0.2247	0.0041	0.8020	0.0039	0.288	0.010	0.00367	0.021991	0.000612
13	0.2131	0.0039	0.7437	0.0035	0.278	0.010	0.00331	0.018818	0.000582
14	0.2003	0.0038	0.6932	0.0032	0.269	0.010	0.00296	0.016112	0.000544
15	0.1898	0.0038	0.6491	0.0030	0.262	0.011	0.00265	0.013923	0.000504
16	0.1781	0.0037	0.6099	0.0028	0.256	0.011	0.00235	0.011986	0.000461
17	0.1715	0.0036	0.5752	0.0026	0.247	0.011	0.00215	0.010495	0.000440
18	0.1637	0.0034	0.5445	0.0023	0.241	0.011	0.00192	0.009093	0.000405
19	0.1577	0.0030	0.5166	0.0019	0.233	0.010	0.00174	0.007947	0.000381
20	0.1506	0.0034	0.4921	0.0020	0.233	0.011	0.00157	0.007158	0.000344

21	0.1433	0.0033	0.4695	0.0019	0.227	0.012	0.00140	0.006211	0.000316
22	0.1386	0.0035	0.4489	0.0019	0.226	0.012	0.00127	0.005596	0.000288
23	0.1355	0.0027	0.4297	0.0014	0.217	0.010	0.00117	0.004949	0.000277
24	0.1302	0.0030	0.4130	0.0015	0.215	0.011	0.00107	0.004483	0.000255
25	0.1259	0.0028	0.3969	0.0013	0.210	0.011	0.00098	0.003995	0.000240
26	0.1206	0.0031	0.3822	0.0014	0.210	0.012	0.00089	0.003614	0.000217
27	0.1178	0.0030	0.3685	0.0013	0.208	0.012	0.00081	0.003272	0.000201
28	0.1116	0.0031	0.3561	0.0012	0.211	0.012	0.00073	0.002980	0.000177
29	0.1094	0.0031	0.3443	0.0012	0.204	0.012	0.00068	0.002695	0.000170
30	0.1076	0.0027	0.3331	0.0010	0.201	0.011	0.00061	0.002395	0.000156
32	0.1003	0.0037	0.3137	0.0011	0.203	0.014	0.00051	0.002018	0.000129
34	0.0939	0.0035	0.2959	0.0010	0.203	0.014	0.00042	0.001668	0.000107
36	0.0916	0.0043	0.2800	0.0010	0.193	0.017	0.00036	0.001352	0.000095
38	0.0821	0.0043	0.2663	0.0008	0.196	0.015	0.00028	0.001078	0.000074
40	0.0829	0.0044	0.2533	0.0008	0.174	0.016	0.00025	0.000854	0.000072
42	0.0837	0.0038	0.2417	0.0006	0.154	0.014	0.00021	0.000653	0.000066
44	0.0777	0.0044	0.2312	0.0005	0.152	0.015	0.00016	0.000502	0.000052
46	0.0681	0.0062	0.2220	0.0006	0.156	0.018	0.00012	0.000390	0.000039

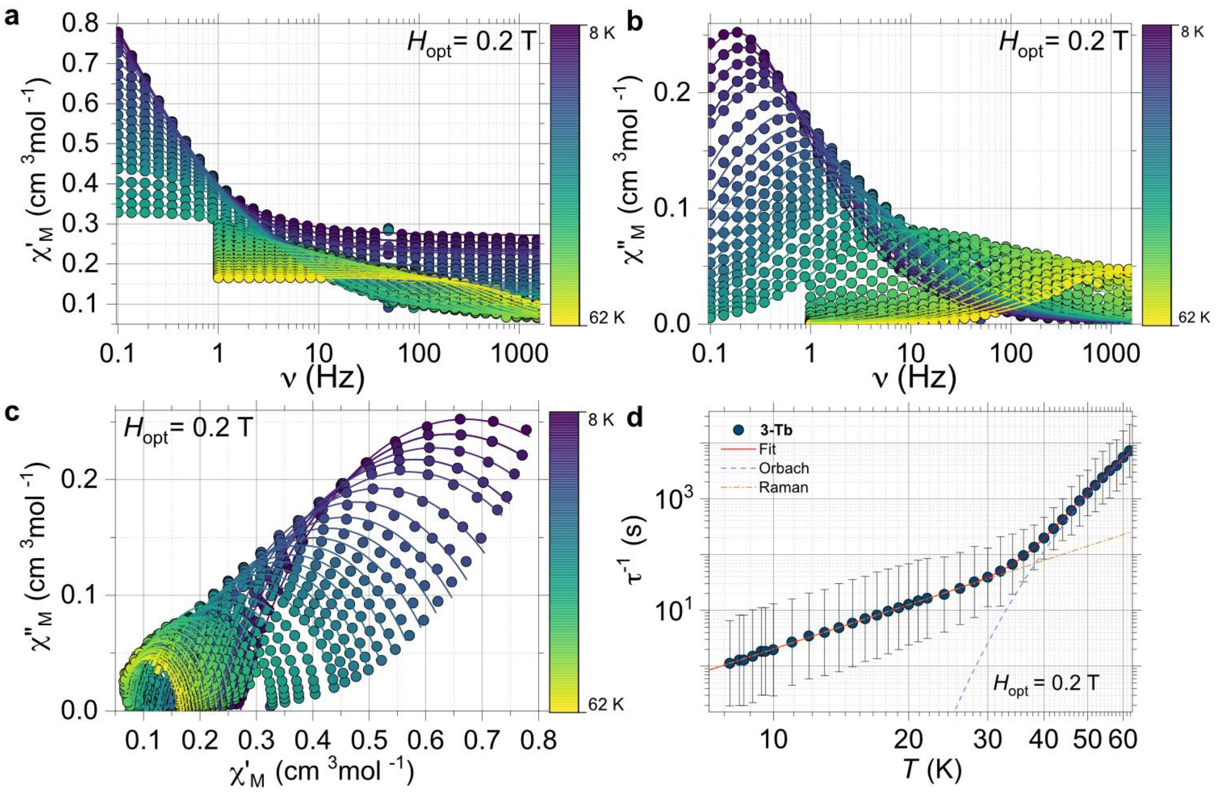

Supplementary Figure 35 AC dynamic susceptibility for **3-Tb** with H_{opt} = 0.2 T. (**a**) $\chi'_M(T;\nu)$, (**b**) $\chi''_M(T;\nu)$, (**c**) Cole-Cole plot at the same field, all in the temperature range of 8 and 62 K. Solid lines are fits to an extended Debye model. Panel (**d**) is the $\tau(T)$ data, associated errors, fit, and contribution of the Raman and Orbach processes to

the overall fit. The error bars represent 1 estimated standard deviation of the distribution of rates. The fit was performed using equation (1) in the main manuscript.

Supplementary Table 6 Fitted parameters for magnetic data of **3-Tb** (H_{opt} = 0.2 T), as depicted in Figure 5b,c, and Supplementary Figure 35 for a simultaneous fit of $\chi''_M(T;\nu)$ and $\chi'_M(T;\nu)$ and associated errors. The τ errors were obtained with equations S1 and S2.

T (K)	χ_S (cm^3 mol^{-1})	χ_S error	χ_T (cm^3 mol^{-1})	χ_T error	α	α err.	τ (s)	τ_upper limit (s)	τ_lower limit (s)
8	0.271	0.014	1.0692	0.1311	0.282	0.079	0.88978	5.161607	0.153384
8.4	0.251	0.003	1.0366	0.0232	0.303	0.015	0.77692	5.022224	0.120187
8.6	0.251	0.003	1.0366	0.0232	0.303	0.015	0.77692	5.022224	0.120187
9	0.235	0.009	0.9933	0.0732	0.312	0.051	0.66367	4.494841	0.097992
9.4	0.230	0.005	0.9300	0.0319	0.292	0.027	0.54317	3.319824	0.088870
9.6	0.230	0.005	0.9301	0.0319	0.292	0.027	0.54318	3.319885	0.088872
10	0.224	0.011	0.9116	0.0680	0.311	0.058	0.50892	3.423935	0.075644
11	0.210	0.003	0.8235	0.0140	0.286	0.016	0.37258	2.209408	0.062829
12	0.190	0.010	0.7640	0.0407	0.285	0.053	0.28784	1.697042	0.048821
13	0.190	0.009	0.7218	0.0362	0.284	0.053	0.25012	1.469425	0.042574
14	0.172	0.002	0.6826	0.0072	0.294	0.012	0.20473	1.264729	0.033141
15	0.162	0.002	0.6435	0.0066	0.292	0.012	0.16977	1.034970	0.027848
16	0.152	0.005	0.6055	0.0149	0.282	0.031	0.14161	0.823391	0.024355
17	0.147	0.002	0.5766	0.0045	0.287	0.010	0.12202	0.726679	0.020489
18	0.141	0.002	0.5457	0.0044	0.278	0.011	0.10426	0.593722	0.018308
19	0.135	0.002	0.5189	0.0039	0.275	0.011	0.09009	0.503640	0.016115
20	0.131	0.002	0.4935	0.0043	0.266	0.013	0.07837	0.419018	0.014658
21	0.124	0.002	0.4719	0.0042	0.268	0.014	0.06783	0.367776	0.012510
22	0.122	0.002	0.4511	0.0037	0.260	0.014	0.06087	0.316074	0.011722
24	0.125	0.015	0.4148	0.0262	0.240	0.115	0.05209	0.245653	0.011046
26	0.112	0.011	0.3826	0.0163	0.226	0.082	0.04072	0.179693	0.009228
28	0.100	0.003	0.3561	0.0040	0.222	0.023	0.03121	0.134818	0.007225
30	0.093	0.011	0.3300	0.0138	0.168	0.092	0.02555	0.085832	0.007606
32	0.089	0.001	0.3189	0.0028	0.215	0.012	0.01997	0.083692	0.004765
34	0.086	0.002	0.2994	0.0034	0.176	0.018	0.01492	0.051810	0.004297
36	0.081	0.001	0.2818	0.0022	0.139	0.014	0.0105	0.030625	0.003600
38	0.080	0.001	0.2660	0.0013	0.108	0.010	0.00743	0.018622	0.002964
40	0.076	0.001	0.2525	0.0011	0.097	0.009	0.00508	0.012048	0.002142
42	0.072	0.001	0.2408	0.0007	0.099	0.007	0.00345	0.008244	0.001444
44	0.070	0.002	0.2299	0.0014	0.097	0.016	0.00238	0.005627	0.001007
46	0.064	0.002	0.2204	0.0014	0.124	0.017	0.00164	0.004453	0.000604

48	0.061	0.003	0.2115	0.0013	0.132	0.019	0.00109	0.003079	0.000386
50	0.060	0.003	0.2032	0.0013	0.112	0.022	7.87E-04	0.002011	0.000308
52	0.058	0.003	0.1966	0.0009	0.145	0.017	5.77E-04	0.001730	0.000192
54	0.057	0.002	0.1895	0.0005	0.141	0.010	4.22E-04	0.001245	0.000143
56	0.053	0.004	0.1829	0.0008	0.150	0.020	3.14E-04	0.000966	0.000102
58	0.058	0.003	0.1770	0.0007	0.135	0.019	2.55E-04	0.000730	0.000089
60	0.055	0.004	0.1716	0.0006	0.144	0.021	1.83E-04	0.000546	0.000061
62	0.052	0.007	0.1660	0.0007	0.144	0.031	1.39E-04	0.000415	0.000046

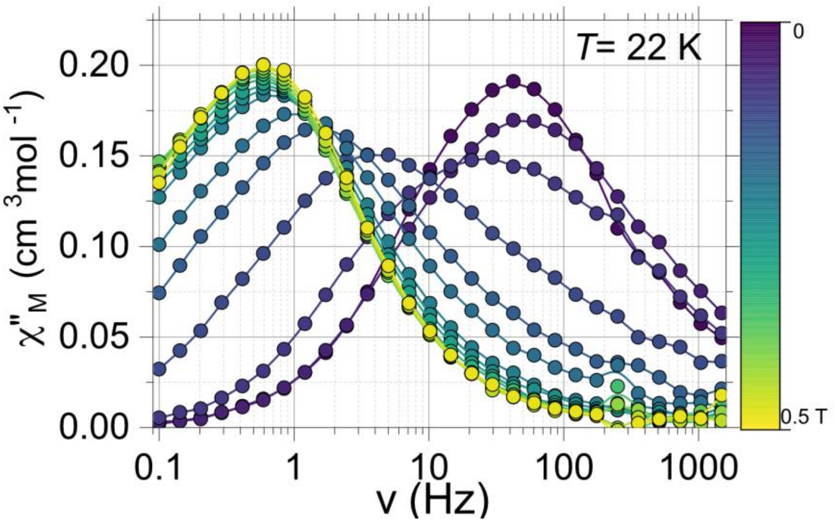

Supplementary Figure 36 Field dependence of χ"M(ν) for **3-Tb** at a fixed temperature of 22 K.

Supplementary Table 7 Fitted parameters for magnetic data field dependence of **3-Tb** (T = 22 K), as depicted in Figure 5d for a simultaneous fit of $\chi''_M(T;\nu)$ and $\chi'_M(T;\nu)$ and associated errors. The τ errors were obtained with equations S1 and S2.

H (T)	χ_S (cm^3 mol^{-1})	χ_S error	χ_T (cm^3 mol^{-1})	χ_T error	α	α err.	τ (s)	$\tau_{\_upper\ limit}$ (s)	$\tau_{\_lower\ limit}$ (s)
0	0.8463	0.0019	0.2455	0.0031	0.2682	0.0064	0.0037	0.01982	0.00068
0.02	0.8475	0.0024	0.2338	0.0046	0.3392	0.0081	0.0028	0.02213	0.00036
0.04	0.8587	0.0039	0.2316	0.0062	0.4211	0.0105	0.0047	0.06100	0.00037
0.06	0.8812	0.0059	0.2293	0.0040	0.4605	0.0093	0.0289	0.48934	0.00170
0.08	0.8876	0.0063	0.2252	0.0023	0.4256	0.0076	0.1029	1.36507	0.00776
0.1	0.8742	0.0058	0.2218	0.0017	0.3869	0.0066	0.1732	1.79259	0.01674
0.15	0.8389	0.0044	0.2105	0.0011	0.3242	0.0052	0.2589	1.87182	0.03582
0.175	0.8279	0.0055	0.2091	0.0013	0.3052	0.0065	0.2837	1.85446	0.04339
0.2	0.8216	0.0049	0.2065	0.0011	0.2949	0.0059	0.2993	1.85440	0.04829
0.25	0.8162	0.0052	0.2060	0.0012	0.2794	0.0062	0.3220	1.84399	0.05621
0.3	0.8145	0.0066	0.2042	0.0015	0.2727	0.0079	0.3321	1.83872	0.05997
0.35	0.8128	0.0055	0.2021	0.0012	0.2653	0.0067	0.3324	1.77440	0.06228
0.4	0.8090	0.0051	0.2021	0.0012	0.2561	0.0063	0.3248	1.65648	0.06368
0.45	0.8068	0.0052	0.2014	0.0012	0.2490	0.0066	0.3149	1.55101	0.06392
0.5	0.8057	0.0055	0.2001	0.0014	0.2446	0.0071	0.3013	1.45307	0.06246

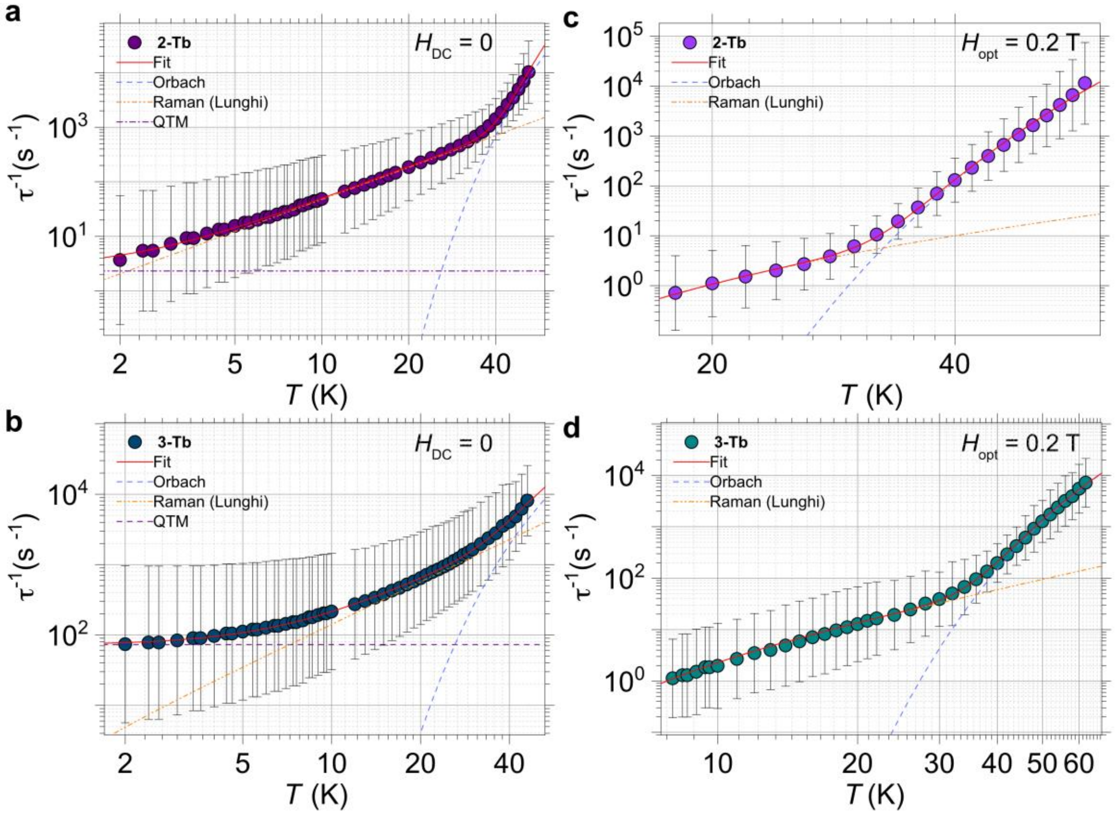

Supplementary Figure 37 Fit of $\tau(T)$ data employing the vibrational dependent Raman process for **2-Tb** and **3-Tb** at (**a**, **b**) zero field and (**c**, **d**) for H_{opt} = 0.2 T, respectively. The parameters for the fits are given in Supplementary Table 8. The error bars represent 1 estimated standard deviation of the distribution of rates.

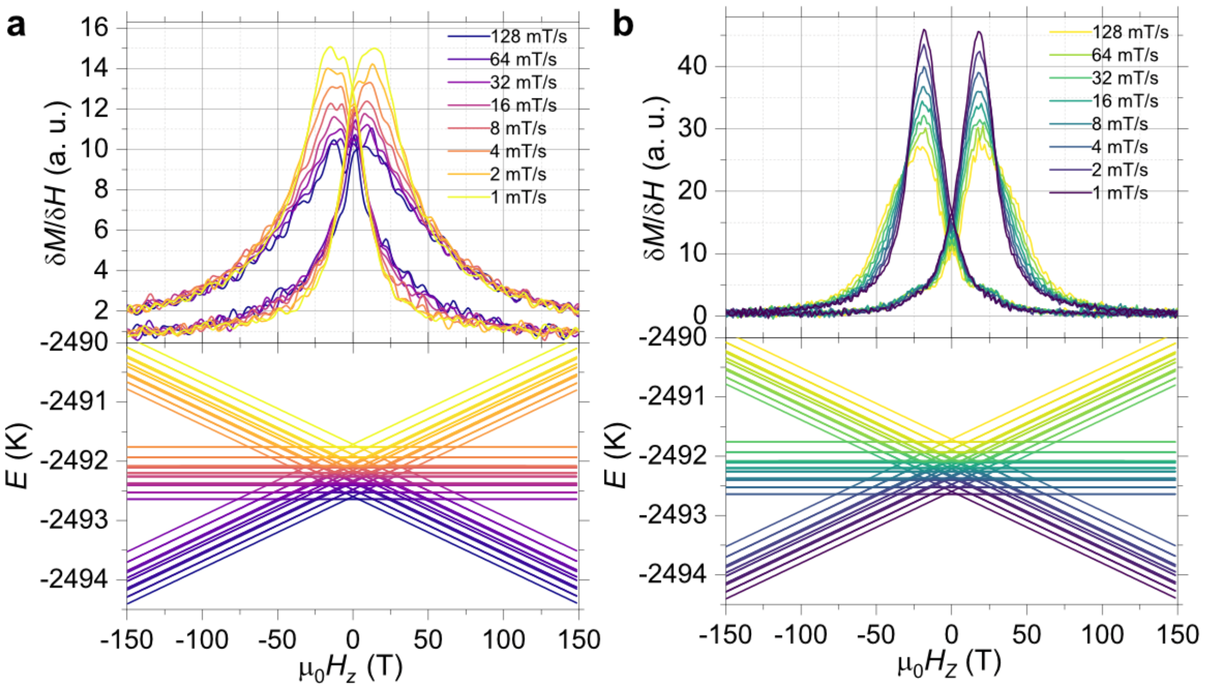

Supplementary Figure 38 Derivative of the µSQUID hysteresis loops (δ*M*/δ*H*) (top) and Zeeman diagram (bottom) for **2-Tb** (**a**) and **3-Tb** (**b**) employing the crystal field parameters as determined from CASSCF calculations, including the hyperfine, quadrupolar interaction, and a dipolar field. The dipolar interaction was obtained considering: $D_{\text{dip}} = \frac{\mu_0 \mu_B^2}{4\pi r^3}\left[\bar{\bar{g}}_A . \bar{\bar{g}}_B - 3\left(\bar{\bar{g}}_A . \vec{R}\right).\left(\vec{R}^T . \bar{\bar{g}}_B\right)\right]$, where μB is the Bohr magneton, *r* is the shortest Tb···Tb intermolecular distance obtained from single-crystal X-ray analysis. $\bar{\bar{g}}_{Ln}$ is the *g*-matrix for the ions, and $\vec{R}$ is the directional unit vector connecting the ions (for simplicity, we set this to [0 1 0]). The Zeeman diagram was obtained employing the following Hamiltonian: $\mathcal{H}_{\text{Ln}}^i = \mathcal{H}_{\text{LF}}^i + g_J \mu_0 \mu_B (\hat{J}_{1z} + \hat{J}_{2z}) H_z + D_{\text{dip}} \hat{J}_1 \cdot \hat{J}_2 + \sum_{i=1}^{2} A_{\text{hyp}} I^i \hat{J}_{\text{iz}} + P_{quad} I_z^i I_z^i$, where $\mathcal{H}_{\text{LF}}^i = \sum_{k=2,4,6,-k \le q \le k} B_k^q O_k^q$ is the ligand field Hamiltonian expressed as Steven's operators (O_k^q), B_k^q. The second term is the Zeeman term, and the third, fourth, and fifth are the dipolar, hyperfine, and quadrupolar interactions, respectively. The ligand field parameters are obtained from CASSCF, and $\hat{J}_{Ln}$, and g_J the spin-orbit, and Landé *g*-factor for Tb(III). The hyperfine and quadrupolar parameters were fixed to the commonly observed values in other molecular systems as described in the main manuscript.

Supplementary Table 8 Fitting parameters for the zero field and in-field data of **2-Tb** and **3-Tb** employing the vibrational dependent Raman mode.

	**2-Tb**		**3-Tb**	
	H_{DC} = 0	H_{opt} = 0.2 T	H_{DC} = 0	H_{opt} = 0.2 T
τ_0 (s)	5.8(8)×10^{-9}	1(3)×10^{-9}	1.1(7)×10^{-6}	1.6(3)×10^{-7}
U_{eff}/k_B (K)	511(7)	541(10)	246(14)	425(5)
C_v (s^{-1})	0.378(3)	54(16)	10(1)	26(2)
ω (cm^{-1})	0.621(3)	55(4)	1.9(4)	18(1)
τ_{QTM} (s)	0.38(5)	-	1.37(2)×10^{-2}	-

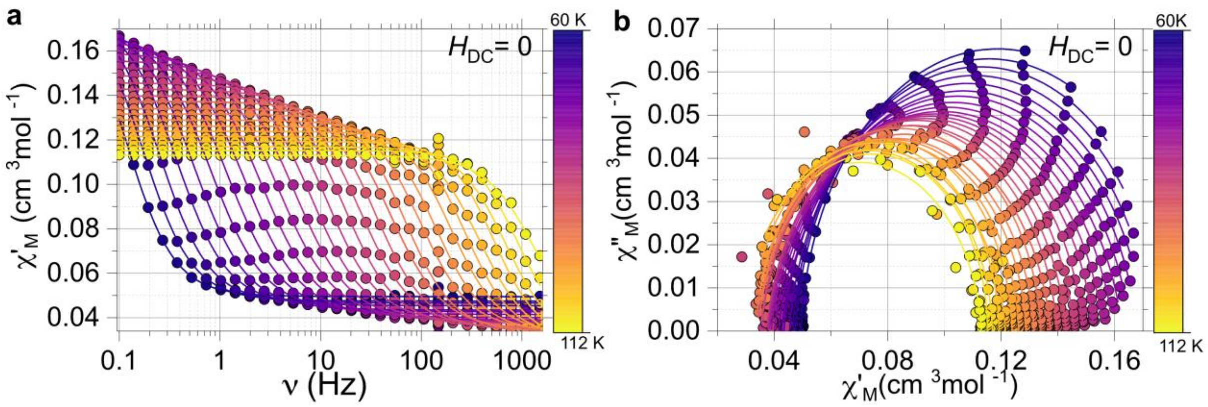

Supplementary Figure 39 (**a**) $\chi'_M(T;\nu)$ for compound **2-Dy** at zero DC field and (**b**) Cole-Cole plot at the same field, both in the temperature range of 2 and 52 K. Solid lines are fits to an extended Debye model.

Supplementary Table 9 Fitted parameters for magnetic data of **2-Dy** (H_{DC} = 0), as depicted in Figure 6a,c, and Supplementary Figure 39 for a simultaneous fit of $\chi''_M(T;\nu)$ and $\chi'_M(T;\nu)$ and associated errors. The τ errors were obtained with equations S1 and S2.

T (K)	χ_S (cm^3 mol^{-1})	χ_S error	χ_T (cm^3 mol^{-1})	χ_T error	α	α err.	τ (s)	$\tau_{\_upper\ limit}$ (s)	$\tau_{\_lower\ limit}$ (s)
60	0.0495	0.0004	0.18791	0.00492	0.037	0.017	1.34481	2.227E+00	8.120E-01
62	0.0475	0.0004	0.18363	0.00310	0.049	0.014	0.92734	1.672E+00	5.143E-01
64	0.0466	0.0004	0.17896	0.00201	0.050	0.011	0.62818	1.139E+00	3.463E-01
66	0.0454	0.0007	0.17587	0.00272	0.062	0.018	0.42685	8.339E-01	2.185E-01
68	0.0434	0.0009	0.17305	0.00293	0.078	0.022	0.28869	6.176E-01	1.349E-01
70	0.0427	0.0008	0.17075	0.00228	0.090	0.019	0.19794	4.514E-01	8.679E-02
72	0.0415	0.0009	0.16808	0.00200	0.096	0.018	0.13505	3.189E-01	5.719E-02
74	0.0400	0.0009	0.16567	0.00170	0.109	0.017	0.09173	2.306E-01	3.649E-02
76	0.0389	0.0009	0.16279	0.00145	0.113	0.016	0.06254	1.606E-01	2.436E-02
78	0.0382	0.0010	0.15965	0.00150	0.112	0.018	0.04266	1.093E-01	1.665E-02
80	0.0378	0.0008	0.15599	0.00099	0.102	0.013	0.02899	7.062E-02	1.190E-02
82	0.0374	0.0009	0.15269	0.00098	0.094	0.014	0.02007	4.676E-02	8.614E-03
84	0.0374	0.0011	0.14896	0.00110	0.080	0.018	0.01391	3.007E-02	6.435E-03
86	0.0354	0.0012	0.14570	0.00106	0.078	0.018	0.00943	2.017E-02	4.408E-03
88	0.0351	0.0005	0.14290	0.00043	0.079	0.008	0.00657	1.411E-02	3.059E-03
90	0.0340	0.0012	0.13976	0.00087	0.067	0.018	0.00456	9.190E-03	2.263E-03
92	0.0343	0.0015	0.13740	0.00099	0.079	0.022	0.00318	6.836E-03	1.479E-03
92	0.0343	0.0015	0.13740	0.00099	0.079	0.022	0.00318	6.836E-03	1.479E-03
94	0.0335	0.0013	0.13402	0.00074	0.043	0.017	0.00231	4.009E-03	1.331E-03
96	0.0342	0.0011	0.13166	0.00054	0.056	0.014	0.00163	3.079E-03	8.629E-04
98	0.0335	0.0010	0.12889	0.00046	0.046	0.013	0.00116	2.053E-03	6.554E-04
100	0.0332	0.0017	0.12620	0.00063	0.038	0.019	8.01E-04	1.338E-03	4.793E-04
102	0.0318	0.0019	0.12385	0.00064	0.024	0.021	5.77E-04	8.623E-04	3.864E-04
104	0.0286	0.0025	0.12149	0.00065	0.061	0.023	4.23E-04	8.232E-04	2.171E-04
106	0.0306	0.0032	0.11924	0.00069	0.011	0.028	3.03E-04	3.987E-04	2.307E-04
108	0.0298	0.0021	0.11691	0.00036	0.026	0.017	2.32E-04	3.543E-04	1.517E-04
110	0.0339	0.0052	0.11515	0.00072	0.024	0.040	1.78E-04	2.682E-04	1.187E-04
112	0.0279	0.0078	0.11307	0.00060	0.056	0.042	1.17E-04	2.203E-04	6.233E-05

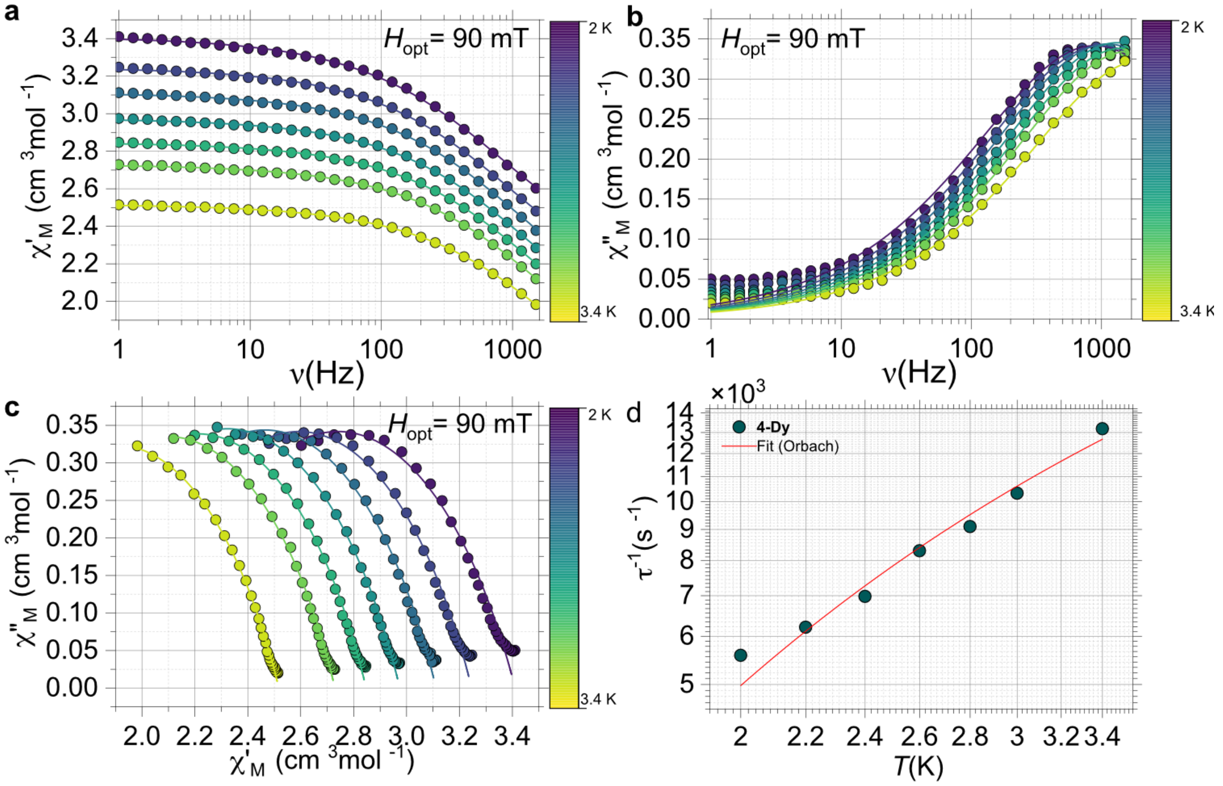

Supplementary Figure 40 AC dynamic susceptibility for **4-Dy** with H_{opt} = 90 mT. (**a**) $\chi'_M(T;\nu)$, (**b**) $\chi''_M(T;\nu)$, (**c**) Cole-Cole plot at the same field, all in the temperature range of 2 and 3.4 K. Solid lines are fits to an extended Debye model. Panel (**d**) is the $\tau(T)$ data, and the fit corresponding to the Orbach process. The fit was performed using equation (1) in the main manuscript.

Supplementary Table 10 Fitted parameters for magnetic data of **4-Dy** (H_{opt} = 90 mT), as depicted in Supplementary Figure 40 for a simultaneous fit of $\chi''_M(T;\nu)$ and $\chi'_M(T;\nu)$ and associated errors. The τ errors were obtained with equations S1 and S2.

T (K)	χ_S (cm^3 mol^{-1})	χ_S error	χ_T (cm^3 mol^{-1})	χ_T error	α	α err.	τ (s)	τ_upper limit (s)	τ_lower limit (s)
2.0	3.4101	0.0043	2.071	0.034	0.399	0.011	1.79E-04	2.002E-03	1.603E-05
2.2	3.2460	0.0035	1.939	0.031	0.387	0.010	1.61E-04	1.664E-03	1.558E-05
2.4	3.1113	0.0029	1.818	0.028	0.378	0.009	1.43E-04	1.406E-03	1.461E-05
2.6	2.9744	0.0024	1.673	0.028	0.379	0.008	1.20E-04	1.187E-03	1.223E-05
2.8	2.8471	0.0021	1.579	0.026	0.376	0.007	1.10E-04	1.063E-03	1.137E-05
3.0	2.7293	0.0017	1.473	0.024	0.376	0.006	9.69E-05	9.417E-04	9.967E-06
3.4	2.5173	0.0014	1.289	0.024	0.382	0.006	7.59E-05	7.643E-04	7.528E-06

VII.4 Magnetization decay

Due to its long relaxation time at low T, the relaxation times for **2-Dy** below 30 K were determined using magnetization decay data.[7] The measurements were performed on a Quantum Design MPMS 3 SQUID magnetometer by increasing the field to a maximum of 7 T and a waiting step of 10 min to magnetically saturate the sample. The field was then switched off as fast as possible (700 Oe/s, linear mode), and the magnetization was recorded during this whole procedure. The starting point t = 0 was taken as the first datapoint with H < 20 Oe. The decay curves were fit to:

$$M(t) = M_{eq} + M_0 \exp[-(\frac{t}{\tau^*})^{\beta}] \quad \text{(S3)}$$

as described by Blackmore et al.[7] The presented relaxation times in Figure 6c of the main text correspond to the definition of [7] as: $e^{<\ln[\tau^*]>}$, where:

$$\langle \ln(\tau) \rangle = \left(1 - \frac{1}{\beta}\right) Eu + ln[\tau^*] \quad \text{(S4)}$$

In S4, Eu is the Euler-Gamma constant. Considering the fitted β parameters and Equation S5:

$$\sigma^2_{\langle \ln \tau \rangle} = \left(\frac{1}{\beta^2} - 1\right) \frac{\pi^2}{6} \quad \text{(S5)}$$

The upper and lower limit of the standard deviation of $M(t)$ was subsequently calculated according to Equation S2.

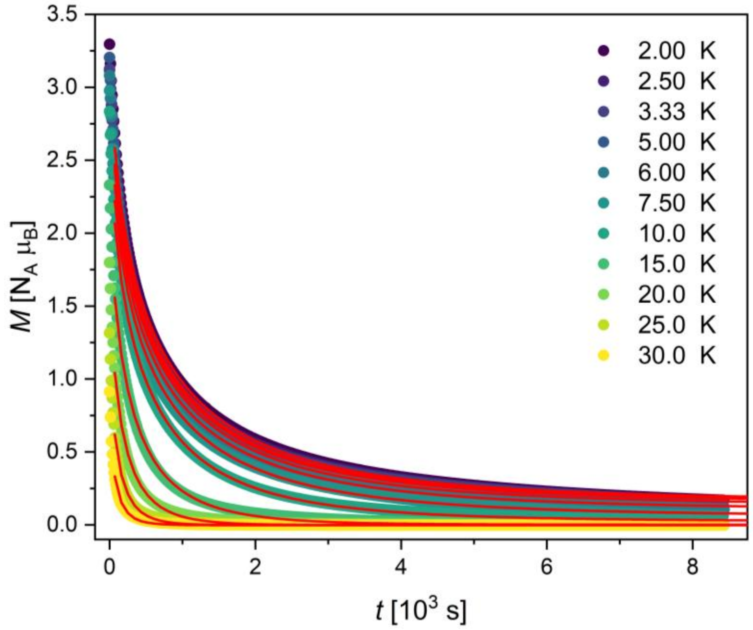

Supplementary Figure 41 Magnetization decay for **2-Dy** recorded up to 30 K. The solid red lines are the best fits to the stretched exponential decay (eq. S3).

Supplementary Table 11 Best fit parameters of the magnetization decay data for **2-Dy** to the stretched exponential decay (eq. S3). And derived $e^{<\ln[\tau^*]>}$ values using (eq. S5).

T	M_{eq}	M_0	τ^* (s)	β	$e^{<\ln[\tau^*]>}$	$\tau_{\_upper\ limit}$ (s)	$\tau_{\_lower\ limit}$ (s)
2	0.16818	3.29504	551.96498	0.5708	357.61748	3.493E+03	8.723E+01
2.5	0.16145	3.11658	565.29013	0.57933	371.74402	3.435E+03	9.302E+01
3.33	0.15704	3.12544	539.55892	0.57201	350.32853	3.394E+03	8.576E+01
5	0.1361	3.20499	506.96846	0.56176	323.16037	3.352E+03	7.668E+01
6	0.10548	3.08118	513.52726	0.56558	329.62335	3.332E+03	7.914E+01
7.5	0.0605	2.97733	507.41305	0.57854	333.22782	3.095E+03	8.319E+01
10	0.02554	2.83463	439.10469	0.61153	304.31547	2.308E+03	8.354E+01
15	9.9648E-4	2.32977	281.50959	0.66201	209.65641	1.203E+03	6.590E+01
20	-0.00237	1.79785	171.11326	0.69576	132.94393	6.431E+02	4.553E+01
25	-0.00238	1.31544	107.25498	0.70696	84.43252	3.869E+02	2.973E+01
30	-0.00203	0.91272	70.71783	0.68982	54.55165	2.717E+02	1.841E+01

VII.5 Ab-initio calculations

To understand the magnetic behavior observed for the systems, *ab initio* calculations were carried out employing the CASSCF/SO-RASSI/SINGLE_ANISO approach implemented in the OpenMolcas package.[8] For the calculations, the crystal structures were employed without further optimizations, and the atoms were described using the standard basis sets from the ANO-RCC library. A basis set of VTZP quality was employed for the Ln^{3+} ions, while VDZP quality was employed for atoms directly bound to the metal centers, and VDZ quality for the remaining atoms, using the second-order DKH transformation. The optimization of the molecular orbitals (MOs) was achieved by state-averaged CASSCF calculations. The active space was defined by the number of unpaired electrons of the metal of interest in the seven 4*f* orbitals. Subsequently, excited states calculations were carried out (RASSCF routine) depending on the spin multiplicity of the metal of interest. The CASSCF wavefunctions were subsequently mixed by spin-orbit coupling, employing the RASSI routine. Lastly, the crystal field decomposition of the lanthanides was extracted with the SINGLE_ANISO module.

Supplementary Table 12 Computed energy levels (the ground state is set at zero), and the main components (>10%) of the wavefunction for each m_J state of the ground-state multiplet $^7F_6$ for the Tb^{3+} in **2-Tb**, at the CASSCF level.

Energy (cm^{-1})	Wavefunction	g_z	Angle (°)
0	100% |±6>	17.9405	-
0	100% |±6>		
316.239	100% |±5>	14.6005	0.4
316.253	100% |±5>		
635.937	99.8% |±4>	11.3142	0.2
636.171	100% |±4>		
934.599	99.2% |±3>	8.1538	1.2
936.351	99.6% |±3>		
1166.464	93.4% |±2>	5.1125	4.8
1194.86	99.4% |±2>		
1303.853	98.8% |±1>	2.6710	13.5
1416.53	99.0% |±1>		
1435.653	92.6% |0>	0.14258	12.4

Supplementary Table 13 Computed energy levels (the ground state is set at zero), and the main components (>10%) of the wavefunction for each m_J state of the ground-state multiplet $^7F_6$ for the Tb^{3+} in **3-Tb**, at the CASSCF level.

Energy (cm^{-1})	Wavefunction	g_z	Angle (°)
0	100% |±6>	17.9397	-
0	100% |±6>		
316.550/	100% |±5>	14.5987	1.1
316.556	100% |±5>		
635.633	99.8% |±4>	11.3112	1.1
635.833	99.8% |±4>		
930.760	99.0% |±3>	8.1717	2.6
939.477	99.8% |±3>		
1173.822	98.6% |±2>	5.2537	8.5
1193.410	97.6% |±2>		
1326.459	98.2% |±1>	2.6842	18.0
1381.224	97.8% |±1>		
1417.106	95.5% |0>	0.0807	38.2

Supplementary Table 14 Computed energy levels (the ground state is set at zero), composition of the *g*-tensor (g_x, g_y, g_z) and the main components (>10%) of the wavefunction for each m_J state of the ground-state multiplet $^6H_{15/2}$ for the Dy^{3+} in **2-Dy**, at the CASSCF level.

Energy (cm^{-1})	g_x	g_y	g_z	Wavefunction	Angle (°)
0	0.0000	0.0000	19.8939	99.8% |±15/2>	-
408.435	0.0014	0.0016	17.0118	99.7% |±13/2>	2.3
672.997	0.0293	0.0305	14.2611	96.6% |±11/2>	1.2
872.225	0.1292	0.1722	11.6188	95.0% |±9/2>	2.8
1043.689	0.1941	0.4762	9.0214	93.6% |±7/2>	3.6
1189.976	0.2485	0.9523	6.3983	92.8% |±5/2>	3.2
1296.165	6.9755	6.4242	3.3645	84.8% |±3/2>+11.0% |±1/2>	3.3
1373.085	0.8316	3.5316	16.9631	11.4%|±3/2>+84.2% |±1/2>	90.0

Supplementary Table 15 Crystal field Hamiltonian is given as $\hat{\boldsymbol{H}}_{\boldsymbol{CF}} = \sum_{k,q} \boldsymbol{B}_{k}^{q} \boldsymbol{O}_{k}^{q}$ and the extended Stevens operator coefficients $\boldsymbol{B}_{k}^{q}$ are extracted from CASSCF calculations for **2-Dy**, **2-Tb** and **3-Tb**.

			B_k^q	
k	*q*	**2-Dy**	**2-Tb**	**3-Tb**
2	-2	-8.54146E-5	-1.18558	-0.50402
2	-1	0.45065	0.09375	-0.51934
2	0	-7.73359	-13.11954	-13.06053
2	1	-1.03001E-4	0.16647	0.023
2	2	1.63731	-0.79396	-0.25173
4	-4	3.42405E-7	-0.00579	0.00923
4	-3	0.02441	-0.05386	0.07514
4	-2	-2.2455E-7	0.01672	0.00922
4	-1	2.15094E-4	-7.67729E-4	0.00508
4	0	-0.00141	0.01316	0.01277
4	1	1.15622E-6	-0.00135	-0.0031
4	2	0.00931	0.01109	0.00812
4	3	1.24452E-6	-0.00602	0.03539
4	4	0.00211	0.00227	-0.00201
6	-6	8.26106E-10	-3.3127E-6	6.42375E-5
6	-5	-3.14004E-4	-3.03235E-4	1.53952E-4
6	-4	-4.05514E-9	1.80382E-5	-7.1448E-5
6	-3	-1.27805E-4	3.37067E-4	-4.8684E-4
6	-2	1.32223E-8	-1.93464E-4	-8.58368E-5
6	-1	-6.29665E-5	-1.33341E-5	3.52794E-5
6	0	-2.40031E-5	-2.44885E-5	-2.14977E-5
6	1	-1.02715E-8	-2.42329E-5	1.29571E-4
6	2	-7.96958E-5	-1.24309E-4	-1.1098E-4
6	3	-2.31391E-8	3.4244E-5	-2.46429E-4
6	4	-1.82748E-6	-8.34838E-6	-3.50714E-6
6	5	4.72146E-8	3.60638E-4	-1.22954E-4
6	6	-1.08015E-5	1.45538E-5	-4.82695E-5

Supplementary Table 16 Average value of the matrix element of the transition magnetic moment (|μ$_x$| + |μ$_y$| + |μ$_z$|)/3 for **2-Dy, 2-Tb** and **3-Tb**.

Multiplet	**2-Dy**	**2-Tb**	**3-Tb**
Matrix Element	Average magnetic moment μB		
$\langle +1.1 \| -1.1 \rangle$	2.87061E-6	2.1637E-10	4.74506E-10
$\langle +2.1 \| -2.1 \rangle$	5.31518E-4	3.17188E-10	5.08282E-10
$\langle +3.1 \| -3.1 \rangle$	0.00997	9.72171E-10	4.00674E-10
$\langle +4.1 \| -4.1 \rangle$	0.05028	1.13199E-9	5.65769E-10
$\langle +5.1 \| -5.1 \rangle$	0.1118	1.84686E-9	4.91571E-10
$\langle +6.1 \| -6.1 \rangle$	0.20039	1.78382E-9	2.48418E-9
$\langle +7.1 \| -7.1 \rangle$	2.22852	2.79628E-8	3.16871E-7
$\langle +8.1 \| -8.1 \rangle$	2.87061E-6	1.58202E-4	8.48689E-6
	Matrix Elements Between Neighbouring Multiplets: $I \rightarrow I + 1$		
Matrix Element	Average magnetic moment μB		
$\langle +1.1 \| +2.1 \rangle$	1.76059	1.80099	1.801
$\langle +1.1 \| -2.1 \rangle$	1.73104E-5	1.7426E-5	9.9402E-6
$\langle +2.1 \| +3.1 \rangle$	2.39413	2.41567	2.4224
$\langle +2.1 \| -3.1 \rangle$	7.81948E-4	5.64769E-4	3.73131E-4
$\langle +3.1 \| +4.1 \rangle$	2.80121	2.77789	2.79246
$\langle +3.1 \| -4.1 \rangle$	0.00991	0.00404	0.02002
$\langle +4.1 \| +5.1 \rangle$	3.07186	2.99176	3.00424
$\langle +4.1 \| -5.1 \rangle$	0.04405	0.08989	0.09918
$\langle +5.1 \| +6.1 \rangle$	3.28417	3.08562	3.1278
$\langle +5.1 \| -6.1 \rangle$	0.13324	0.67915	0.43203
$\langle +6.1 \| +7.1 \rangle$	3.38284	2.20073	2.24362
$\langle +6.1 \| -7.1 \rangle$	0.24652	2.23794	2.26614
$\langle +7.1 \| +8.1 \rangle$	2.67157	1.88373	1.84069
$\langle +7.1 \| -8.1 \rangle$	0.46379	1.88086	1.82044
	Matrix Elements Between Neighboring Multiplets: $I \rightarrow I + 2$		
Matrix Element		Average magnetic moment μB	
$\langle +1.1 \| +3.1 \rangle$	0.11762	0.02477	0.04652
$\langle +1.1 \| -3.1 \rangle$	2.76529E-4	4.68326E-5	4.48418E-5
$\langle +2.1 \| +4.1 \rangle$	0.13778	0.02773	0.05238
$\langle +2.1 \| -4.1 \rangle$	0.00339	7.32425E-4	7.50754E-4
$\langle +3.1 \| +5.1 \rangle$	0.25327	0.06844	0.08961
$\langle +3.1 \| -5.1 \rangle$	0.01517	0.00377	0.02211

$\langle +4.1 \vert +6.1\rangle$	0.26108	0.14609	0.16152
$\langle +4.1 \vert -6.1\rangle$	0.05457	0.03857	0.05161
$\langle +5.1 \vert +7.1\rangle$	0.21872	0.18295	0.15916
$\langle +5.1 \vert -7.1\rangle$	0.12313	0.19313	0.15852
$\langle +6.1 \vert +8.1\rangle$	0.20482	0.04994	0.02888
$\langle +6.1 \vert -8.1\rangle$	0.57309	0.05059	0.03789

Matrix Elements Between Neighboring Multiplets: *I* → *I* + 3

Matrix Element		Average magnetic moment μ_B	
$\langle +1.1 \vert +4.1\rangle$	0.07276	0.02618	0.02541
$\langle +1.1 \vert -4.1\rangle$	3.40846E-4	2.27002E-4	1.23161E-4
$\langle +2.1 \vert +5.1\rangle$	0.06735	0.07163	0.05889
$\langle +2.1 \vert -5.1\rangle$	0.00103	0.00293	0.01011
$\langle +3.1 \vert +6.1\rangle$	0.07221	0.16853	0.11691
$\langle +3.1 \vert -6.1\rangle$	0.00527	0.04941	0.04703
$\langle +4.1 \vert +7.1\rangle$	0.13394	0.28493	0.20448
$\langle +4.1 \vert -7.1\rangle$	0.02614	0.25713	0.18501
$\langle +5.1 \vert +8.1\rangle$	0.06172	0.05119	0.05139
$\langle +5.1 \vert -8.1\rangle$	0.05985	0.05039	0.04643

Matrix Elements Between Neighboring Multiplets: *I* → *I* + 4

Supplementary Table 17 Average value of the matrix element of the transition magnetic moment ($|\mu_x| + |\mu_y| + |\mu_z|$)/3 for **2-Dy, 2-Tb,** and **3-Tb**.

Multiplet	**2-Dy**	**2-Tb**	**3-Tb**
Matrix Element	Average magnetic moment μ_B		
⟨+1.1|+5.1⟩	0.03216	0.00728	0.00861
⟨+1.1|−5.1⟩	6.0089E-4	5.03475E-4	7.0765E-4
⟨+2.1|+6.1⟩	0.04529	0.01372	0.01808
⟨+2.1|−6.1⟩	0.00184	0.00152	0.00809
⟨+3.1|+7.1⟩	0.03501	0.0514	0.06826
⟨+3.1|−7.1⟩	0.01938	0.06139	0.06103
⟨+4.1|+8.1⟩	0.02107	0.128	0.13764
⟨+4.1|−8.1⟩	0.02702	0.12732	0.1241
Matrix Elements Between Neighboring Multiplets: $I \rightarrow I + 5$			
Matrix Element	Average magnetic moment μ_B		
⟨+1.1|+6.1⟩	0.01677	0.00337	0.00461
⟨+1.1|−6.1⟩	3.44073E-4	5.95396E-4	0.00149
⟨+2.1|+7.1⟩	0.02998	0.08473	0.08981
⟨+2.1|−7.1⟩	0.00623	0.08368	0.10132
⟨+3.1|+8.1⟩	0.02982	0.15796	0.16109
⟨+3.1|−8.1⟩	0.01654	0.15662	0.15789
Matrix Elements Between Neighboring Multiplets: $I \rightarrow I + 6$			
Matrix Element	Average magnetic moment μ_B		
⟨+1.1|+7.1⟩	0.00489	0.13154	0.13171
⟨+1.1|−7.1⟩	9.93219E-4	0.12894	0.12982
⟨+2.1|+8.1⟩	0.01084	0.19719	0.18677
⟨+2.1|−8.1⟩	0.00594	0.19741	0.20729
Matrix Elements Between Neighboring Multiplets: $I \rightarrow I + 7$			
Matrix Element	Average magnetic moment μ_B		
⟨+1.1|+8.1⟩	0.00164	0.13046	0.1351
⟨+1.1|−8.1⟩	0.00122	0.13027	0.12483

Supplementary Table 18 Properties of the four quasi-doublets of **4-Dy**, at the CASSCF level.

Energy (cm^{-1})	Δ_{tun} (cm^{-1})	g_z	Angle (°)
0.000<br>6.4	6.4	19.5579	-
525.8<br>613.6	87.7	14.0458	10.2
958.6<br>1336.9	378	9.0302	13.1
1428.0<br>1605.2		9.3385	93.6

VIII. Density functional theory calculations

VIII.1 General remarks

Quantum chemical calculations at the density functional theory (DFT) level were performed with TURBOMOLE.[9,10] Optimized structures of all compounds considered in the calculations are provided in the separate ASCII file optimized-structures.txt as part of the Supporting Information.

All calculations were performed with the exact two-component (X2C) Hamiltonian[11,12] in its local variant (DLU-X2C)[13] within the finite nucleus model (parameters taken from Ref. 14) using the TPSSh hybrid functional[15,16] x2c-TZVPall basis sets for Tb, Dy, Sn, and the ring C atoms of the stannole ligands, and x2c-SV(P)all basis sets for all remaining atoms.[17] The resolution of the identity approximation for the Coulomb part (RI-*J*)[18] in combination with the corresponding auxiliary basis sets as well as the multipole-accelerated RI-*J* approximation (MARI-*J*)[19] were employed. The D4 dispersion correction was used.[20] Self-consistent field (SCF) thresholds were set to 10^{-7} E_h, and medium sized grids (gridsize 3 or 3a)[21,22] were used for the numerical integration of the exchange-correlation terms.

Calculations concerning magnetically induced ring currents were performed with the GIMIC program[23-25] with a preceding calculation of the magnetically (un)perturbed densities as part of a nuclear shielding calculation with TURBOMOLE. A threshold of 10^{-7} a.u. was used for the response of the orbitals (norm of the residuum) in the coupled-perturbed Kohn-Sham (CPKS) equations.

Visualizations of molecular structures, molecular orbitals, and integration planes were prepared with Chemcraft (Version 1.8).[26] Magnetically induced current density contour plots were visualized with OriginPro (Version 2023, OriginLab Corporation, Northampton, MA, USA).

VIII.2 Electron configuration of Dy(II) in 4-Dy

While for compounds **2-Tb**, **2-Dy**, and **3-Tb**, featuring trivalent lanthanide ions, a [Xe]4f^n electron configuration of the central lanthanide ion is expected, a [Xe]4f^{10} or [Xe]4$f^9$5d^1 electron configuration is conceivable for the central Dy(II) ion in **4-Dy**. Thus, geometry optimizations of **4-Dy** were performed with 4 unpaired electrons (corresponding to a [Xe]4f^{10} electron configuration) and 6 unpaired electrons (corresponding to a [Xe]4$f^9$5d^1 electron configuration), respectively, using a Fermi-smearing approach[27] with a fixed number of unpaired electrons. From the Ln-Ct distances and Ct-Ln-Ct' angles calculated for these two cases it is evident that the experimental results are only reproduced when assuming a [Xe]4$f^9$5d^1 electron configuration for the Dy(II) ion (refer to Supplementary Table 19). Natural population analyses (NPA),[28] validating that the targeted electron configurations were obtained, are provided in Supplementary Tables 20 and 21.

Supplementary Table 19 Calculated and experimentally determined Ln-Ct distances and Ct-Ln-Ct' angles.

	**Ln-Ct1**	**Ln-Ct2**	**Ct-Ln-Ct'**
**4-Dy** with 4 unpaired electrons	2.359 Å	2.439 Å	166.1°
**4-Dy** with 6 unpaired electrons	2.304 Å	2.298 Å	176.7°
**4-Dy** Experimental	2.303 Å	2.303 Å	180.0°

Supplementary Table 20 NPA results obtained for compound **4-Dy** with four unpaired electrons.

**Natural electron configuration**	[core] 6s(0.19) 7s(0.01) 6p(0.02) 5d(0.64) 6d(0.19) 4f(9.56)					
**Atomic populations from total density**	charge	n(s)	n(p)	n(d)	n(f)	n(g)
	1.41500	10.18479	24.00792	20.82974	9.56240	0.00016
**Atomic populations from spin density**	sum	n(s)	n(p)	n(d)	n(f)	n(g)
	4.30275	0.00396	0.02196	-0.12498	4.40181	0.00000

Supplementary Table 21 NPA results obtained for compound **4-Dy** with six unpaired electrons.

**Natural electron configuration**	[core] 6s(0.20) 7s(0.01) 6p(0.02) 5d(0.93) 6d(0.22) 4f(9.15)					
**Atomic populations from total density**	charge	n(s)	n(p)	n(d)	n(f)	n(g)
	1.49195	10.19881	24.00591	21.14936	9.15378	0.00019
**Atomic populations from spin density**	sum	n(s)	n(p)	n(d)	n(f)	n(g)
	5.55842	0.03978	0.10472	0.58930	4.82458	0.00003

VIII.3 Relative stannole ligand orientation

Experimentally, it is evident that the tin atoms of the two distinct stannole ligands in **2-Tb**, **2-Dy** and **3-Tb** face in the same direction, while the tin atoms of the two distinct stannole ligands in **4-Dy** point in opposite directions. For compounds **2-Tb** and **2-Dy**, the observed relative orientation seems intuitive given that the highly polarizable tin atoms are attracted to the potassium counter ion by electrostatic interactions. However, in compounds **3-Tb** and **4-Dy** the potassium counter ion is not in close spatial proximity to the tin atoms, thus preventing it from directly exerting an effect on the relative orientation of the stannole ligands. To investigate whether there is an intrinsic preference for any of the relative orientations of the tin atoms, the simplified model systems shown in Supplementary Figure 42 were constructed computationally, and their geometries optimized using the computational settings outlined above (refer to Chapter VI.1). x2c-TZVPall basis sets were used for all atoms in these calculations. Counter ions were omitted and the negative charges compensated using the conductor-like screening model (COSMO)[29] with the Gaussian charge model[30] (Lebedev gridsize 3) and otherwise default parameters.

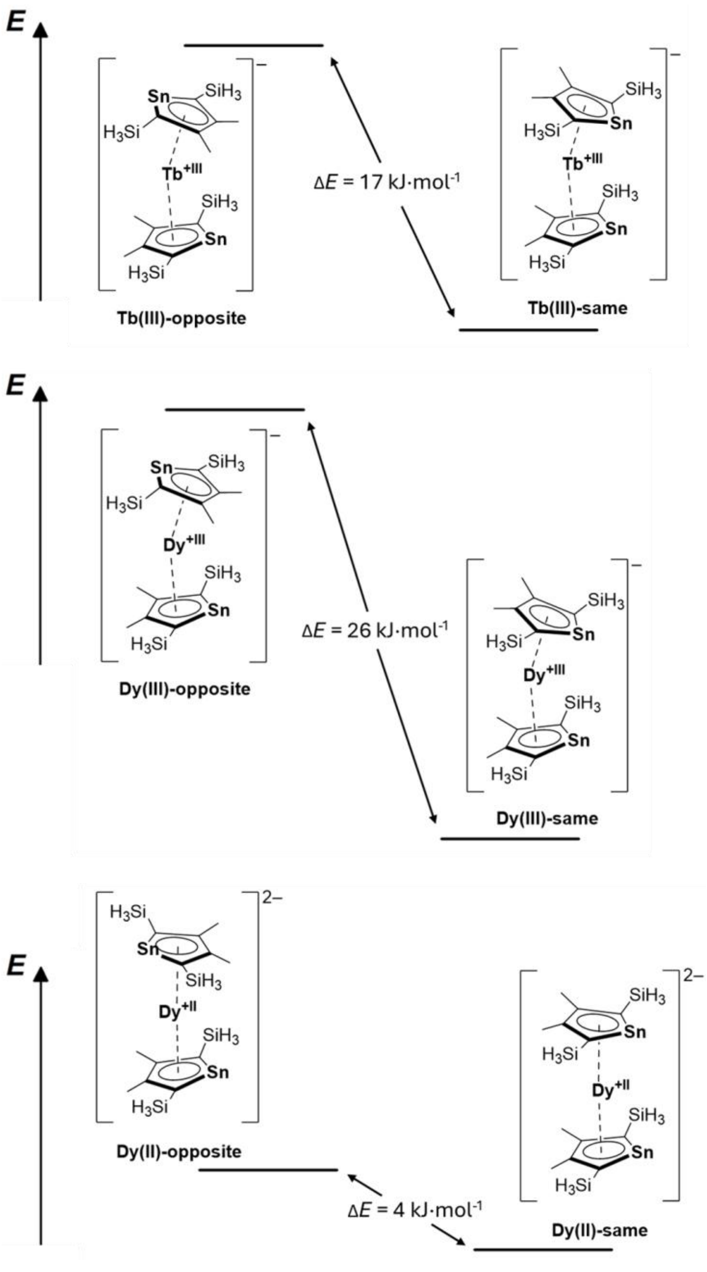

Supplementary Figure 42 Chemical structures of and relative energy differences between the model systems for the investigation of the relative stannole ligand orientation.

For the trivalent model systems, orientations with the tin atoms of the two distinct stannole ligands facing the same direction are energetically preferred by about 17 kJ·mol^{-1} (Tb, Supplementary Figure 42 top) and 26 kJ·mol^{-1} (Dy, Supplementary Figure 42 center), respectively. For the divalent dysprosium model systems, this orientation is energetically preferred by about 4 kJ·mol^{-1} over the alternative with the two tin atoms facing in opposite directions (Supplementary Figure 42 bottom). Thus, from an energetic perspective, there is no significant energetic preference for any of the two relative orientations of the tin atoms, especially in the case of the divalent dysprosium model systems. In conclusion, the experimentally observed differences between the relative tin atom orientations in **2-Tb/2-Dy/3-Tb** and **4-Dy** do not seem to stem from an intrinsic preference for any of the relative orientations due to reasons of electronic structure but are likely the result of subtle packing effects in the solid state.

In addition to the *meta*-GGA hybrid functional TPSSh, we tested a GGA hybrid functional, PBE0,[31-33] also in its local hybrid variant, LH14t-calPBE,[34] and further the quality of the x2c-TZVPall basis by employing a larger quadruple-zeta basis (x2c-QZVPall)[35] with the original functional TPSSh (refer to Supplementary Supplementary Table ***22***). The impact of the basis on the energy differences is less than 1 kJ·mol^{-1} and thus negligible; for reasons of computational demand, only single point calculations were performed with the x2c-QZVPall basis. Altering the functional leads to somewhat larger changes of the energy differences. However, all calculations indicate that for Dy(II), Dy(II)-opposite and Dy(II)-same are virtually isoenergetic (differences 3-5 kJ·mol^{-1}). For the trivalent model systems, orientations with the tin atoms of the two distinct stannole ligands facing the same direction remain energetically preferred (12-17 kJ·mol^{-1} for Tb(III) and 12-27 kJ·mol^{-1} for Dy(III)). We note that pure DFT functionals were not tested as they give too high energies for the *f* shell, yielding sometimes even wrong *f* shell occupations.

Supplementary Table 22 Energy differences between structures of the model systems (refer to Supplementary Figure 42) with the tin atoms of the two distinct stannole ligands facing the same and opposite directions with different functionals and basis sets. In all cases, orientations with the tin atoms of the two distinct stannole ligands facing the same direction are energetically preferred.

	**TPSSh/ x2c-TZVPall**	**TPSSh/x2c-QZVPall (TPSSh/x2c-TZVPall geometry)**	**PBE0/ x2c-TZVPall**	**LH14t-calPBE/ x2c-TZVPall**
**Tb(III)**	16.9	17.4	12.4	13.4
**Dy(III)**	26.5	25.8	11.8	17.4
**Dy(II)**	4.1	4.5	5.0	3.2

VIII.4 Ct-Ln-Ct' angle within 4-Dy

Another experimental finding concerning the molecular structures of the compounds is that the structure of **2-Dy** changes from a bent sandwich motif (Ct-Ln-Ct': 154°) to a linear structure in **4-Dy** (Ct-Ln-Ct': 180°) upon complexation of the potassium counter ion by 18-crown-6.

Fundamentally, there are two distinct differences between **2-Dy** and **4-Dy**: (*i*) The presence (**2-Dy**) or absence (**4-Dy**) of the potassium counter ion in close spatial proximity to the stannole ligands, and (*ii*) the formal oxidation state of the central dysprosium ion (+III in **2-Dy**, +II in **4-Dy**). As indicated by the fact that no significant change of the Ct-Ln-Ct' bending angle occurs for **2-Tb** (Ct-Ln-Ct': 154°) compared to **3-Tb** (Ct-Ln-Ct': 155°), point (*i*) outlined above seems unplausible as the cause for the difference in the Ct-Ln-Ct' bending angle of **2-Dy** compared to **4-Dy**, leaving point (*ii*) as the dominant determining factor. The fact that the electronic situation of the central dysprosium ion plays a key role for the geometric structure of the complex is also indicated by the strong influence of the electron configuration of the Dy(II) ion on the geometric structure as outlined above (refer to Chapter VIII.2).

Close inspection of the bonding situation between the Dy(II) ion and the stannole ligands in **4-Dy** revealed bonding interactions between the occupied *d* orbital on the Dy(II) ion and orbitals with dominant *p* character at the tin atoms of both stannole ligands as is evident from Mulliken population analyses[36] of the relevant orbitals (refer to 43 and Supplementary Table 23).

Due to the tin atoms of the stannole ligands pointing in different directions, it is intuitive that the overlap between the orbitals relevant for the discussed bonding interaction is maximized in a linear geometry with a Ct-Ln-Ct' bending angle of 180°. We note, however, that other effects, such as subtle packing effects in the solid state, might also contribute to the observed effects on the Ct-Ln-Ct' bending angle.

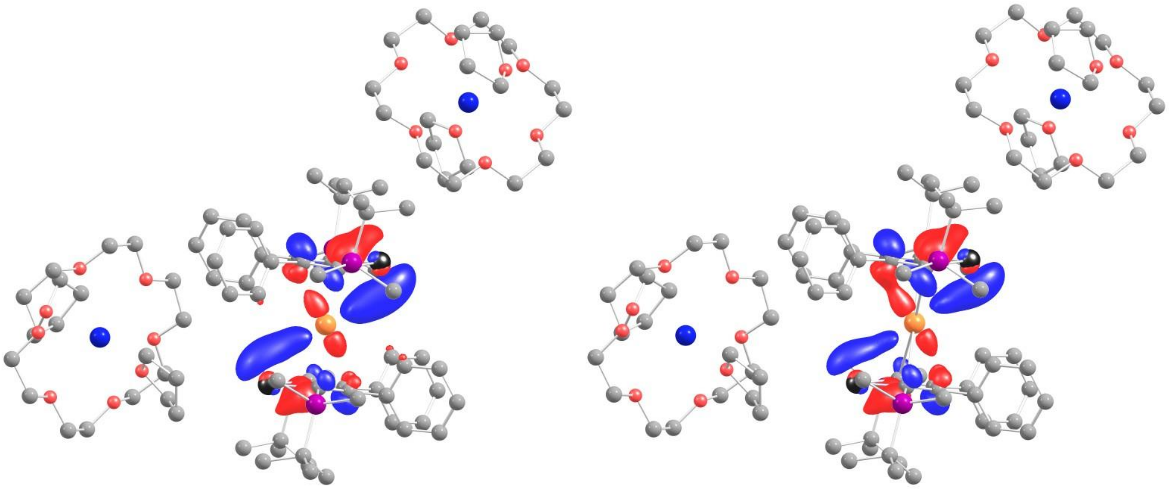

Supplementary Figure 43 α- (left) and β- (right) spin orbitals of compound **4-Dy** relevant for covalent Dy-stannole bonding. Contours are drawn at ±0.04 atomic units. Hydrogen atoms are omitted for clarity.

Supplementary Table 23 Results of Mulliken population analysis of the molecular orbitals depicted in Supplementary Figure 43. Labels refer to the numbering of the atoms within the provided coordinate file (optimized-structures.txt).

atom	total	s	p	d	f
***α*-orbital**					
1dy	0.182	0.084	0.001	0.097	0.000
2sn	0.205	0.008	0.194	0.003	0.001
3sn	0.150	0.001	0.145	0.003	0.001
4c	0.065	-0.001	0.064	0.002	0.000
5c	0.048	0.000	0.046	0.001	0.000
6c	0.032	0.002	0.026	0.003	0.000
7c	0.024	0.001	0.020	0.002	0.000
8c	0.036	0.002	0.031	0.003	0.000
9c	0.023	0.001	0.019	0.003	0.000
10c	0.060	0.000	0.058	0.002	0.000
11c	0.052	0.000	0.050	0.001	0.000
***β*-orbital**					
1dy	0.147	0.058	0.001	0.088	0.000
2sn	0.169	0.004	0.162	0.002	0.001
3sn	0.131	0.000	0.129	0.002	0.001
4c	0.083	-0.002	0.083	0.002	0.000
5c	0.054	0.000	0.052	0.002	0.000
6c	0.039	0.002	0.033	0.004	0.000
7c	0.031	0.002	0.027	0.003	0.000
8c	0.043	0.002	0.038	0.004	0.000
9c	0.030	0.001	0.026	0.003	0.000
10c	0.078	0.000	0.076	0.002	0.000
11c	0.056	0.000	0.055	0.002	0.000

VIII.5 Stannole aromaticity

To probe the aromatic character of the stannole ligands in compounds **2-Tb**, **2-Dy**, **3-Tb** and **4-Dy** based on the magnetic criterion,[37] magnetically induced ring current susceptibilities were calculated using the GIMIC program. The results are summarized in Supplementary Table 24 and show a net diatropic ring current in all cases, indicating an aromatic character of the coordinated stannole ligands. The calculated ring current susceptibilities of **2-Tb** and **3-Tb** are almost identical, indicating that the location of the potassium counter ion only has a minimal effect on the aromatic character of the stannole ligands. The ring current susceptibility calculated for **2-Dy** is comparable to that of **2-Tb** and **3-Tb**. Compound **4-Dy** shows a slightly reduced net diatropic ring current.

Supplementary Table 24 Magnetically induced ring current susceptibilities (in nA/T) of the stannole ligands in **2-Tb**, **2-Dy**, **3-Tb** and **4-Dy**.

	Stannole ligand 1	Stannole ligand 2
**2-Tb**	8.1	8.2
**2-Dy**	7.6	7.6
**3-Tb**	8.5	8.0
**4-Dy**	5.3	4.6

To obtain ring current susceptibilities, the magnetically induced current density of a given stannole ring was integrated in a plane perpendicular to the ring plane as well as the C-C bond opposite the tin atom (see below for graphical illustrations). The magnetic field was applied perpendicular to the plane defined by the stannole ring under consideration. The spatial dimensions of the integration plane need to be chosen in such a way that it contains all relevant contributions of the ring current originating from the stannole ring, while ideally not containing any influences of ring currents of the central lanthanide ions or the stannole ring substituents.

To choose reasonable integration boundaries, 2D contour plots of the magnetically induced current densities within a suitably chosen plane were prepared for all complexes which graphically illustrate the ring currents flowing along the C-C bond opposite the tin atom of the considered stannole ring and around the central lanthanide ion (see below). Based on these plots, integration boundaries covering the main contributions of the ring current flowing along the stannole ring, while minimizing influences originating from the central lanthanide ion and the stannole ring substituents, were determined graphically. The chosen integration boundaries are stated below for the individual complexes (refer to Supplementary Figure 44, 46, 48 and 50). The integration planes are visualized in Supplementary Figure 45, 47, 49 and 51.

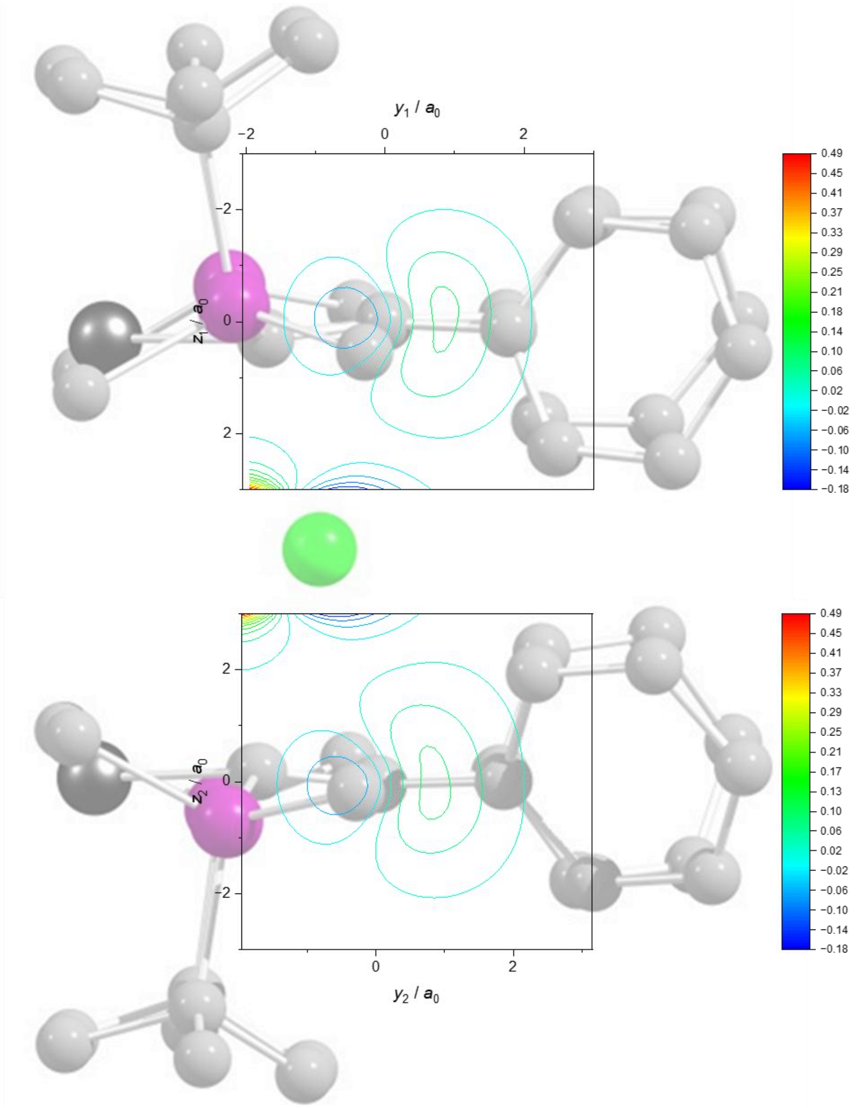

Supplementary Figure 44 Cutout of the molecular structure of **2-Tb** superimposed with 2D contour plots showing the magnetically induced current densities within a plane perpendicular to the stannole ring plane and the C-C bond opposite the tin atom of the stannole ligand under consideration. Color scale refers to magnetically induced current density in atomic units. Based on the 2D contour plots, the following integration intervals were determined graphically: $y_1 \in [-2.05a_0;2.5a_0]$, $z_1 \in [-3a_0;2a_0]$ and $y_2 \in [-2.05a_0;2.5a_0]$, $z_2 \in [-3a_0;2.1a_0]$. The integration planes are visualized in Supplementary Figure 45.

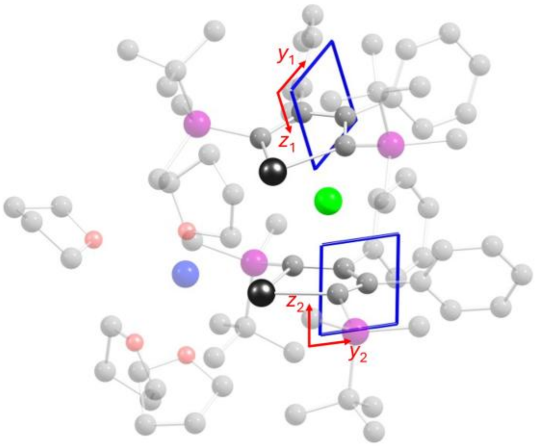

Supplementary Figure 45 Graphical illustration of the integration planes (blue) used for the calculation of the magnetically induced ring current susceptibilities of the stannole ligands in **2-Tb**.

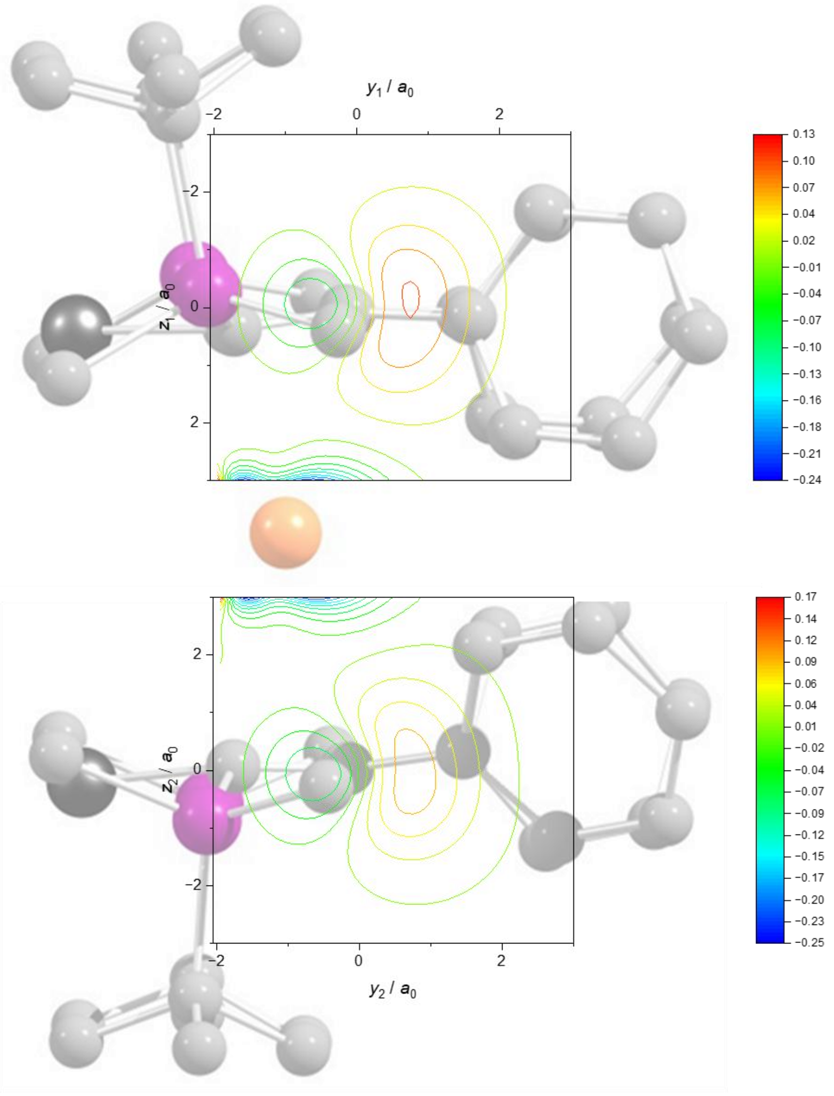

Supplementary Figure 46 Cutout of the molecular structure of **2-Dy** superimposed with 2D contour plots showing the magnetically induced current densities within a plane perpendicular to the stannole ring plane and the C-C bond opposite the tin atom of the stannole ligand under consideration. Color scale refers to magnetically induced current density in atomic units. Based on the 2D contour plots, the following integration intervals were determined graphically: $y_1 \in [-2.05a_0;2.5a_0]$, $z_1 \in [-3a_0;2.2a_0]$ and $y_2 \in [-2.05a_0;2.5a_0]$, $z_2 \in [-3a_0;2.2a_0]$. The integration planes are visualized in Supplementary Figure 47.

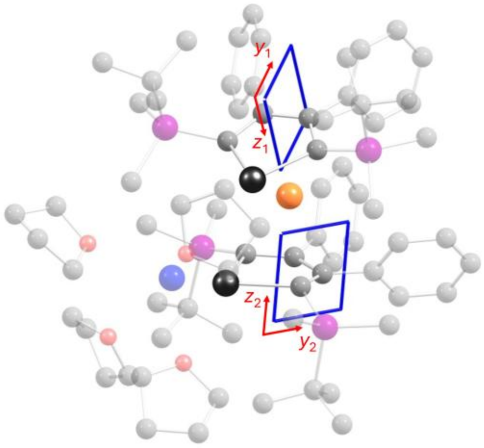

Supplementary Figure 47 Graphical illustration of the integration planes (blue) used for the calculation of the magnetically induced ring current susceptibilities of the stannole ligands in **2-Dy**.

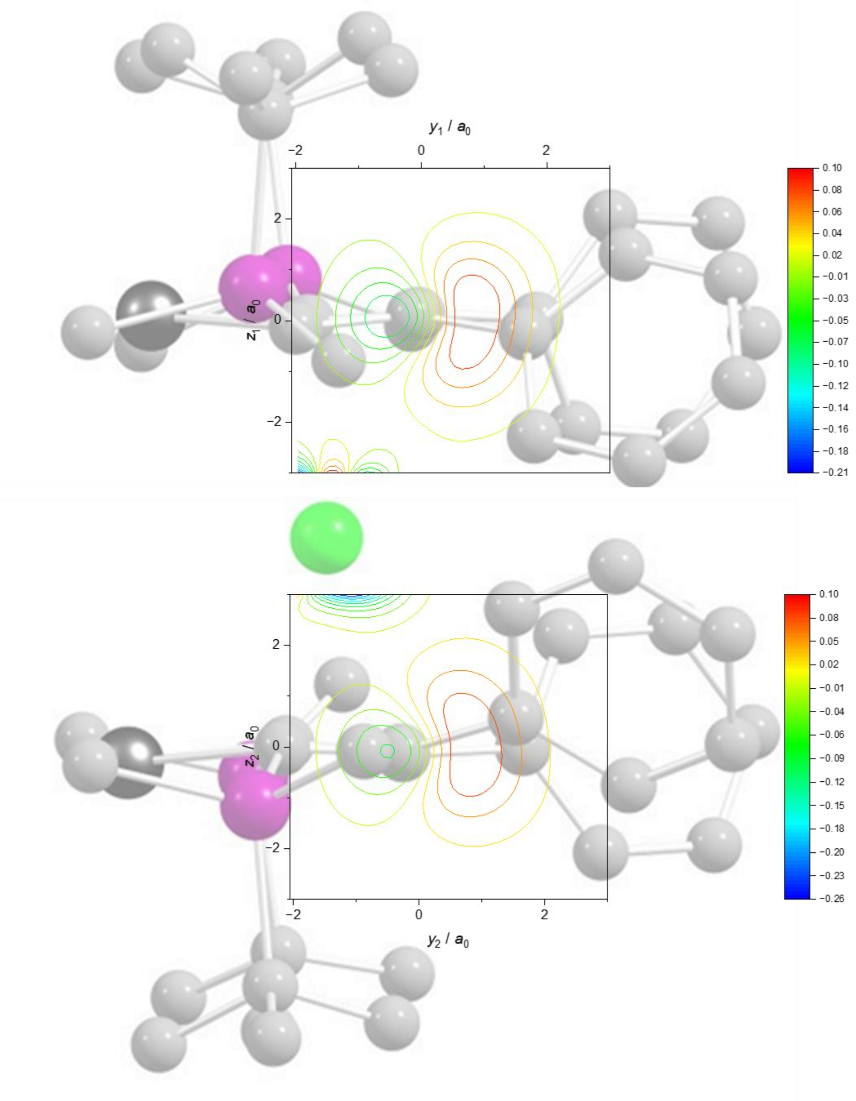

Supplementary Figure 48 Cutout of the molecular structure of **3-Tb** superimposed with 2D contour plots showing the magnetically induced current densities within a plane perpendicular to the stannole ring plane and the C-C bond opposite the tin atom of the stannole ligand under consideration. Color scale refers to magnetically induced current density in atomic units. Based on the 2D contour plots, the following integration intervals were determined graphically: $y_1 \in [-2.06a_0;2.5a_0]$, $z_1 \in [-2.35a_0;3a_0]$ and $y_2 \in [-2.06a_0;2.5a_0]$, $z_2 \in [-3a_0;2.15a_0]$. The integration planes are visualized in Supplementary Figure 49.

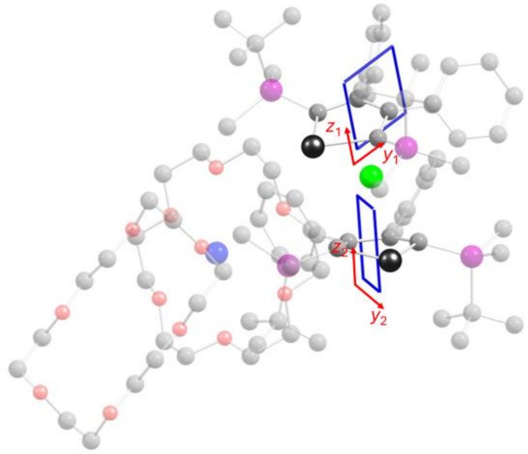

Supplementary Figure 49 Graphical illustration of the integration planes (blue) used for the calculation of the magnetically induced ring current susceptibilities of the stannole ligands in **3-Tb**.

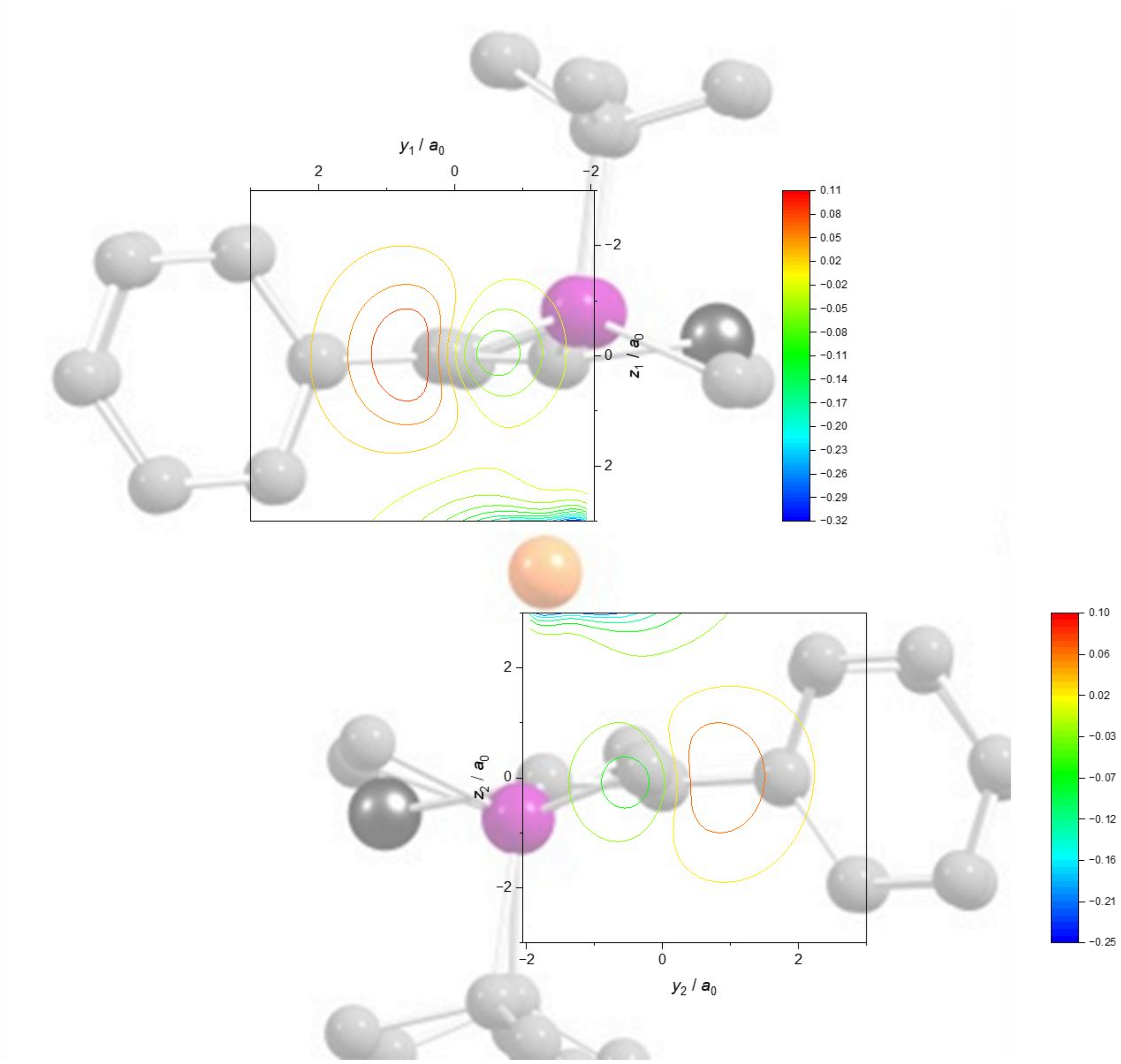

Supplementary Figure 50 Cutout of the molecular structure of **4-Dy** superimposed with 2D contour plots showing the magnetically induced current densities within a plane perpendicular to the stannole ring plane and the C-C bond opposite the tin atom of the stannole ligand under consideration. Color scale refers to magnetically induced current density in atomic units. Based on the 2D contour plots, the following integration intervals were determined graphically: $y_1 \in [-2.05a_0;2.5a_0]$, $z_1 \in [-3a_0;1.9a_0]$ and $y_2 \in [-2.06a_0;2.5a_0]$, $z_2 \in [-3a_0;1.9a_0]$. The integration planes are visualized in Supplementary Figure 51.

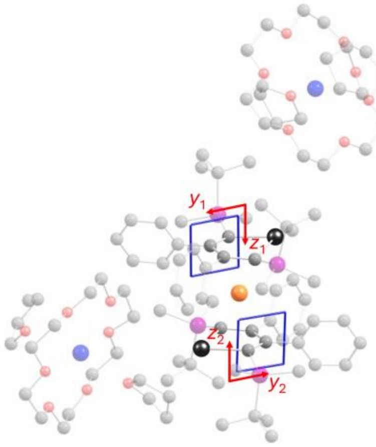

Supplementary Figure 51 Graphical illustration of the integration planes (blue) used for the calculation of the magnetically induced ring current susceptibilities of the stannole ligands in **4-Dy**.

IX. References

[1] Kuwabara, T. *et al.* Enhancement of Stannylene Character in Stannole Dianion Equivalents Evidenced by NMR and Mössbauer Spectroscopy and Theoretical Studies of Newly Synthesized Silyl-Substituted Dilithiostannoles. *Organometallics* **33**, 2910-2913 (2014).

[2] G. Meyer, P. Ax, An analysis of the ammonium chloride route to anhydrous rare-earth metal chlorides, *Mat. Res. Bull.*, **17**, 1447–1455 (1982).

[3] G. Sheldrick, A short history of SHELX, *Acta Cryst. A,* **64**, 112-122 (2008).

[4] G. Sheldrick, Crystal structure refinement with SHELXL, *Acta Cryst. C*, **A71**, 3-8. (2015).

[5] O. V; Dolomanov, L, J, Bourhis, R. J. Gildea, J. A. K. Howard, H. Puschmann, OLEX2: a complete structure solution, refinement and analysis program, *J. Appl. Crystallogr*. **42**, 339-341 (2009).

[6] Zorn, R. Logarithmic moments of relaxation time distributions. *J. Chem. Phys*. **116**, 3204–3209 (2002).

[7] Blackmore, W. J. A. *et al.* Characterisation of magnetic relaxation on extremely long timescales. *Phys. Chem. Chem. Phys.* **25**, 16735-16744 (2023).

[8] Aquilante, F. *et al.* Molcas 8: New capabilities for multiconfigurational quantum chemical calculations across the periodic table. *J. Comput. Chem.* **37**, 506-541 (2016).

[9] TURBOMOLE V. 7.9 2024, a development of University of Karlsruhe and Forschungszentrum Karlsruhe GmbH, 1989-2007, TURBOMOLE GmbH, since 2007; available from https://turbomole.org.

[10] Franzke, Y. J. *et al.* TURBOMOLE: Today and Tomorrow. *J. Chem. Theory Comput.* **19***,* 6859-6890 (2023).

[11] Peng, D., Middendorf, N., Weigend, F. & Reiher, M. An efficient implementation of two-component relativistic exact-decoupling methods for large molecules. *J. Chem. Phys.* **138**, 184105 (2013).

[12] Franzke, Y. J., Middendorf, N. & Weigend, F. Efficient implementation of one- and two-component analytical energy gradients in exact two-component theory. *J. Chem. Phys.* **148**, 104110 (2018).

[13] Peng, D. & Reiher, M. Local relativistic exact decoupling. *J. Chem. Phys.* **136**, 244108 (2012).

[14] Visscher, L. & Dyall, K. G. Dirac-Fock atomic electronic structure calculations using different nuclear charge distributions. *At. Data Nucl. Data Tables* **67**, 207-224 (1997).

[15] Tao, J. & Perdew, J. P. Climbing the Density Functional Ladder: Nonempirical Meta–Generalized Gradient Approximation Designed for Molecules and Solids. *Phys. Rev. Lett.* **91**, 146401 (2003).

[16] Staroverov, V. N., Scuseria, G. E., Tao, J. & Perdew, J. P. Comparative assessment of a new nonempirical density functional: Molecules and hydrogen-bonded complexes. *J. Chem. Phys.* **119**, 12129-12137 (2003).

[17] Pollak, P. & Weigend, F. Segmented Contracted Error-Consistent Basis Sets of Double- and Triple-ζ Valence Quality for One- and Two-Component Relativistic All-Electron Calculations. *J. Chem. Theory Comput.* **13**, 3696-3705 (2017).

[18] Weigend, F. Accurate Coulomb-fitting basis sets for H to Rn. *Phys. Chem. Chem. Phys.* **8**, 1057-1065 (2006).

[19] Sierka, M., Hogekamp, A. & Ahlrichs, R. Fast evaluation of the Coulomb potential for electron densities using multipole accelerated resolution of identity approximation. *J. Chem. Phys.* **118**, 9136-9148 (2003).

[20] Caldeweyher, E., Bannwarth, C. & Grimme, S. Extension of the D3 dispersion coefficient model. *J. Chem. Phys.* **147**, 034112 (2017).

[21] Treutler, O. & Ahlrichs, R. Efficient molecular numerical integration schemes. *J. Chem. Phys*. **102**, 346–354 (1995).

[22] Franzke, Y. J., Treß. R., Pazdera, T. M. & Weigend, F. Error-consistent segmented contracted all-electron relativistic basis sets of double- and triple-zeta quality for NMR shielding constants. *Phys. Chem. Chem. Phys.* **21**, 16658-16664 (2019).

[23] Jusélius, J., Sundholm, D. & Gauss, J. Calculation of current densities using gauge-including atomic orbitals. *J. Chem. Phys.* **121**, 3952-3963 (2004).

[24] Fliegl, H., Taubert, S., Lehtonen, O. & Sundholm, D. The gauge including magnetically induced current method. *Phys. Chem. Chem. Phys.* **13**, 20500-20518 (2011).

[25] Taubert, S., Sundholm, D. & Jusélius, J. Calculation of spin-current densities using gauge-including atomic orbitals. *J. Chem. Phys.* **134**, 054123 (2011).

[26] Zhurko G. A. Chemcraft - graphical program for visualization of quantum chemistry computations. Ivanovo, Russia, 2005. https://chemcraftprog.com.

[27] Rabuck, A. D. & Scuseria, G. E. Improving self-consistent field convergence by varying occupation numbers. *J. Chem. Phys.* **110**, 695-700 (1999).

[28] Reed, A. E., Weinstock, R. B. & Weinhold, F. Natural population analysis. *J. Chem. Phys.* **83**, 735-746 (1985).

[29] Schäfer, A., Klamt, A., Sattel, D., Lohrenz, J. C. W., & Eckert, F. COSMO Implementation in TURBOMOLE: Extension of an efficient quantum chemical code towards liquid systems. *Phys. Chem. Chem. Phys.* **2**, 2187-2193 (2000).

[30] Pausch, A. Consistent Analytical Second Derivatives of the Kohn–Sham DFT Energy in the Framework of the Conductor-Like Screening Model through Gaussian Charge Distributions. *J. Chem. Theory Comput.* **20**, 3169-3183 (2024).

[31] Perdew, J. P. & Wang, Y. Accurate and simple analytic representation of the electron-gas correlation energy. *Phys. Rev. B* **45**, 13244–13249 (1992).

[32] Perdew, J. P., Burke, K. & Ernzerhof, M. Generalized gradient approximation made simple. *Phys. Rev. Lett.* **77**, 3865–3868 (1996).

[33] Perdew, J. P., Ernzerhof, M. & Burke, K. Rationale for mixing exact exchange with density functional approximations. *J. Chem. Phys.* **105**, 9982–9985 (1996).

[34] Arbuznikov, A. V. & Kaupp, M. Towards improved local hybrid functionals by calibration of exchange-energy densities. *J. Chem. Phys.* **141**, 204101 (2014).

[35] Franzke, Y. J., Spiske, L., Pollak, P. & Weigend, F. Segmented contracted error-consistent basis sets of quadruple-ζ valence quality for one- and two-component relativistic all-electron calculations. *J. Chem. Theory Comput.* **16**, 5658–5674 (2020).

[36] Mulliken, R. S. Electronic Population Analysis on LCAO–MO Molecular Wave Functions. I. *J. Chem. Phys.* **23**, 1833-1840 (1955).

[37] Gershoni-Poranne, R. & Stanger, A. Magnetic criteria of aromaticity. *Chem. Soc. Rev.* **44**, 6597-6615 (2015).
